# Effective Population Size Estimation in Large Marine Populations: Considering Current Challenges and Opportunities When Simulating Large Data Sets With High‐Density Genomic Information

**DOI:** 10.1111/eva.70121

**Published:** 2025-07-28

**Authors:** Chrystelle Delord, Sophie Arnaud‐Haond, Agostino Leone, Ekaterina Noskova, Rémi Tournebize, Patrick Jacques, Francis Marsac, Natacha Nikolic

**Affiliations:** ^1^ MARBEC, Univ Montpellier, CNRS, Ifremer, IRD La Réunion France; ^2^ MARBEC, Univ Montpellier, CNRS, Ifremer, IRD Sète France; ^3^ Department of Earth and Marine Sciences (DiSTeM) University of Palermo Palermo Italy; ^4^ National Biodiversity Future Center Palermo Italy; ^5^ Department of Biology University of Fribourg Fribourg Switzerland; ^6^ University of Edinburgh Edinburgh United Kingdom; ^7^ IRD, UMR DIADE Montpellier France; ^8^ Universite de Pau et des Pays de l’Adour, INRAE, AQUA, ECOBIOP Saint‐Pée‐sur‐Nivelle France; ^9^ Centre de Recherche Sur la Biodiversité et l'Environnement (CRBE) Université de Toulouse, CNRS, IRD, Toulouse INP, Université Toulouse 3 – Paul Sabatier (UT3) Toulouse France; ^10^ ARBRE – Agence de Recherche pour la Biodiversité à La Réunion Saint‐Gilles France

**Keywords:** allele frequency spectra, computational genetics, conservation genomics, effective population size, fisheries management, linkage disequilibrium

## Abstract

Next‐generation‐sequencing has broadened perspectives regarding the estimation of the effective population size (*Ne*) by providing high‐density genomic information. These technologies have expanded data collection and analytical tools in population genetics, increasing understanding of populations with high abundance, such as marine species with high commercial or conservation priority. Several common methods for estimating *Ne* are based on allele frequency spectra or linkage disequilibrium between loci. However, their specific constraints make it difficult to apply them to large populations, especially with confounding factors such as migration rates, complex sampling schemes or non‐independence between loci. Computer simulations have long represented invaluable tools to explore the influence of biological or logistical factors on *Ne* estimation and to assess the robustness of dedicated methods. Here, we outline several *Ne* estimation methods and their foundational principles, requirements and likely caveats regarding application to populations of high abundance. Thereafter, we present a simulation framework built upon recent computational genomic tools that combine the possibility to generate biologically realistic data sets with realistic patterns of long‐term neutral genetic diversity. This framework aims at reproducing and tracking the main critical features of data derived from a large natural population when running a simulation‐based population genetics study, for example, evaluating the strengths and limitations of various *Ne* estimation methods. We illustrate this framework by generating genotype data sets with varying sample sizes and locus numbers and analysing them with three software tools (NeEstimator2, GONE and GADMA). Detailed and annotated simulation scripts are provided to ensure reproducibility and to support future research on *Ne* estimation. These resources can support method comparisons and validations, particularly for non‐specialists, such as conservation practitioners and students.

## 1 Introduction

Population genetics has heavily contributed to describing the structure and evolutionary trajectories of marine populations of conservation concern (Bierne et al. 2016; Selkoe et al. 2016), such as abundant and heavily exploited large pelagic species (e.g., tuna and other large top‐predator representing the most important fisheries outcome worldwide, FAO 2022). The application of population genetics to fisheries management allows, for example, better delimiting the geographical contours of the different stocks exploited or bycaught and thus focusing management measures on more relevant geographical scales (Benestan 2020; Nikolic et al. 2023; Leone et al. 2024). The advent of high‐throughput sequencing and genomic approaches has provided additional tools for conventional stock assessment (e.g., based on catch‐per‐unit data), the detection of selection pressures (including those exerted by fishing) and the temporal monitoring of genetic diversity levels within populations (Ovenden et al. 2015; Casey et al. 2016). In particular, the use of population genomics tools to estimate stock abundance (census and effective population size) has led to much debate in the scientific community regarding the relevance and prerequisites of these tools. This is partly due to the potential estimation biases for highly abundant, vagile and fecund marine species (Ovenden et al. 2016; Waples 2016). Nevertheless, the effective population size (*Ne*) is an essential indicator of genetic diversity and the adaptive potential of populations, making it a key variable to estimate in conservation (Leroy et al. 2017; Hoban et al. 2022). The effective size also provides valuable information compared to the population census abundance *Nc* (Waples 2024a), although there lacks a clear relationship between *Ne* and *Nc*, including among marine species (Palstra and Fraser 2012; Delord et al. 2024). Estimates of *Ne* that are close to actual abundance values have been reported for some elasmobranch species, such as the grey shark (Portnoy et al. 2009) and the leopard shark (Dudgeon and Ovenden 2015), whereas many more imbalanced ratios have been reported in similar species, such as the Galapagos shark (Pazmiño et al. 2017), the blue shark (King et al. 2015) and the curl ray (Chevolot et al. 2008) or in less closely related species such as the albacore tuna (Laconcha et al. 2015). This difficulty in establishing a clear relationship can be attributed to both the biological characteristics of the species and the biased application of certain *Ne* and/or *Nc* estimation methods (Palstra and Fraser 2012) when certain underlying assumptions are not met. Consequently, it remains difficult to reliably deduce one parameter (*Nc* or *Ne*) from the other.

Genetic methods for estimating *Ne* and total abundance in natural populations are diverse, and each is associated with specific features and limitations. Systematic reviews of *Ne* estimation methods are numerous and provide extensive information about their ideal conditions of application (Wang and Caballero 1999; Wang 2005; Gilbert and Whitlock 2015; Wang et al. 2016; Waples 2016, 2024b; Waples et al. 2016; Nadachowska‐Brzyska et al. 2022). However, their applicability to large, abundant populations is rarely the main topic (but see Wang 2016). In addition, the potential of high‐throughput‐sequencing data sets for improving *Ne* estimates (Waples 2016) has rarely been addressed with respect to *Ne* estimation by various classes of methods for large populations. Simulation tools in population genetics offer means of comparing the performance of various *Ne* estimation methods (Wang 2016; Marandel et al. 2019; Reid and Pinsky 2022). Applying those tools often requires identifying the potential sources of biases inherent to each method in order to explicitly take them into account while running simulations. The ‘ideal’ simulation software program should be able to incorporate those sources of biases and may not always be easy to apprehend, especially when simulating high‐density genomic data, large sample and population sizes or complex evolutionary scenarios.

In this article, we first focus on two past and contemporary *Ne* estimation classes of methods applicable with high‐density genomic data sets from non‐model species, namely (1) linkage disequilibrium methods and (2) allele frequency spectrum‐based methods (Table 1). For each method, we recall the principles and main documented strengths and limitations of their general applications to estimate diploid *Ne* at various spatial and temporal scales. We further discuss the specific challenges associated with targeting populations of large abundance as well as the potential advantages and issues brought by high‐density genetic data sets. Second, we present an easily reproducible simulation framework, in the form of several annotated scripts, which is based on the computational genomic simulation software programs SLiM (Haller and Messer 2016, 2018) and *msprime* (Kelleher et al. 2016). This framework currently enables the production of high‐density genotypic data sets for moderate sample and population sizes and was developed with the pedagogical purpose of being accessible to non‐specialists. We present this simulation framework along with a preliminary comparative analysis of the results obtained with the two classes of methods mentioned above, with the hope that this framework can be further developed and improved to test the relative performance of *Ne* estimation methods for populations of high abundance.

**TABLE 1:** Summary of the methods and software programs mentioned in this article.

Class	Estimation method and rationale	Software programs	Key features	Examples
Linkage disequilibrium‐based methods	Calculation of a standardised linkage disequilibrium statistic ($r^{2}$) to summarise patterns of LD between unlinked pairs of loci, including a systematic correction for sampling bias $r^{2}$ could be calculated within a maximum‐likelihood framework (Hill 1981) or upon a composite statistic obtained through the Burrows method (Weir 1979) Corrections are necessary to take sources of pseudo‐replication into account with high‐density, genomic data	LDNe NeEstimator2	Provide contemporary *Ne* estimates. Potential sources of biases are very well known and documented Does not require recombination information but assumes independence between loci	Puncher et al. (2018) Waples, Grewe, et al. (2018) Waples (2024b) See also Table A1.1: Appendix S1
		SPEEDNe	Provides contemporary *Ne* estimates Does not require recombination information but assumes independence between loci SPEEDNe proposes various ways of handling rare alleles with highly polymorphic loci and to compute confidence intervals so several estimates could be obtained and compared Requires basic knowledge of MATLAB. Potential memory issues when handling large data sets (e.g., SNP data)	Hamilton et al. (2018) Lorenzana et al. (2020) Maguire et al. (2023)
	Estimation based on patterns of linkage disequilibrium between loci with known recombination distances	SNeP	Allows for the precise estimation of contemporary and historical *Ne* values from more or less distant time periods, based on sets of loci characterised by different genetic distances Requires a large number of loci (typically in the order of 10^4^). May suffer from the same sources of bias that classical LD‐based contemporary *Ne* estimation methods (e.g., population structure) Confidence intervals need to be calculated manually	Barbato et al. (2015) Martinez et al. (2022)
		LinkNe	Provides contemporary and/or recent past *Ne* estimates, for example, enabling to assess *Ne* trends over the last few generations Could be used with a moderate number of loci (e.g., ~10^3^ SNPs). Enables to generate confidence intervals May suffer from the same sources of bias that classical LD‐based contemporary *Ne* estimation methods (e.g., population structure)	Hollenbeck et al. (2016) Lehnert et al. (2019)


moments‐LD		Provides contemporary and historical *Ne* values from more or less distant time periods Enables to perform model selection and demography parameters estimation, thus taking into account potentially complex demography trajectories and estimating local *Ne* for distinct sub‐populations Can be used in association with a genetic algorithm (GADMA or GADMA2) to improve model selection	Ragsdale and Gravel (2019, 2020) Noskova et al. (2020, 2023)
GONE	Provides contemporary and/or recent past *Ne* estimates, for example, enabling to assess *Ne* trends over the last few generations Implements a genetic algorithm so as to detect and assess *Ne* fluctuations across recent timescales	Santiago et al. (2020) Martinez et al. (2022)
SFS‐based methods	Model selection and demography parameter estimation (including *Ne* at various time periods) comparing the observed site frequency spectra from one population or more to their theoretical SFS, obtained using exact calculation through diffusion equations	δaδi	Enables to handle scenarios of complex demographies with up to five populations. Uses exact calculation for theoretical SFS with several recent improvements (e.g., Portik et al. 2017) Can be used in association with a genetic algorithm (GADMA or GADMA2) to improve model selection	Gutenkunst et al. (2009)
		moments	Enables to handle scenarios of complex demographies with up to five populations. Uses exact calculation for theoretical SFS using a mathematical framework faster than δaδi's, but potentially less accurate in some cases Can be used in association with a genetic algorithm (GADMA or GADMA2) to improve model selection	Jouganous et al. (2017)
	Model selection and demography parameter estimation (including *Ne* at various time periods) comparing the observed site frequency spectra from one population or more to their theoretical SFS, obtained under the continuous‐time Moran model	momi2	Enables to handle scenarios of very complex demographies with overlapping generations and up to 10 populations or more but does not handle continuous gene flow Can be used in association with a genetic algorithm (GADMA or GADMA2) to improve model selection	Kamm et al. (2017, 2020)

Model selection and demography parameter estimation (including *Ne* at various time periods) comparing the observed site frequency spectra from one population or more to their theoretical SFS, obtained under a coalescent model	fastSimcoal2	Enables to handle scenarios of very complex demographies with theoretically infinite number of populations Does not use exact calculation to obtain the theoretical SFS hence being much faster, but potentially less accurate in detecting very recent events than other methods relying on exact calculations	Excoffier et al. (2013, 2021) Hoey et al. (2022)

In this work, we focus on methods that do not require haplotype or phasing information. These other classes of methods (such as isolation‐by‐descent and SMC approaches; see Fournier et al. 2023) are challenging to apply to non‐model species, such as large pelagic species, where thorough genomic information is generally scarce (e.g., where there is no reference genome or recombination map).

## 2 Effective Size Estimation Based on Linkage Disequilibrium

Several genetic methods for estimating *Ne* rely on linkage disequilibrium (Palstra and Fraser 2012; Marandel et al. 2019), which occurs when alleles at different loci are found together more often than expected by chance due to physical linkage or population‐level processes. Linkage disequilibrium is fundamentally a statistical measure of alleles association, whether due to physical linkage on the genome or population‐level processes. In the latter case, such as genetic drift or population structure, these associations arise from biases in allele transmission, rather than physical proximity between loci.

**FIGURE 1:**
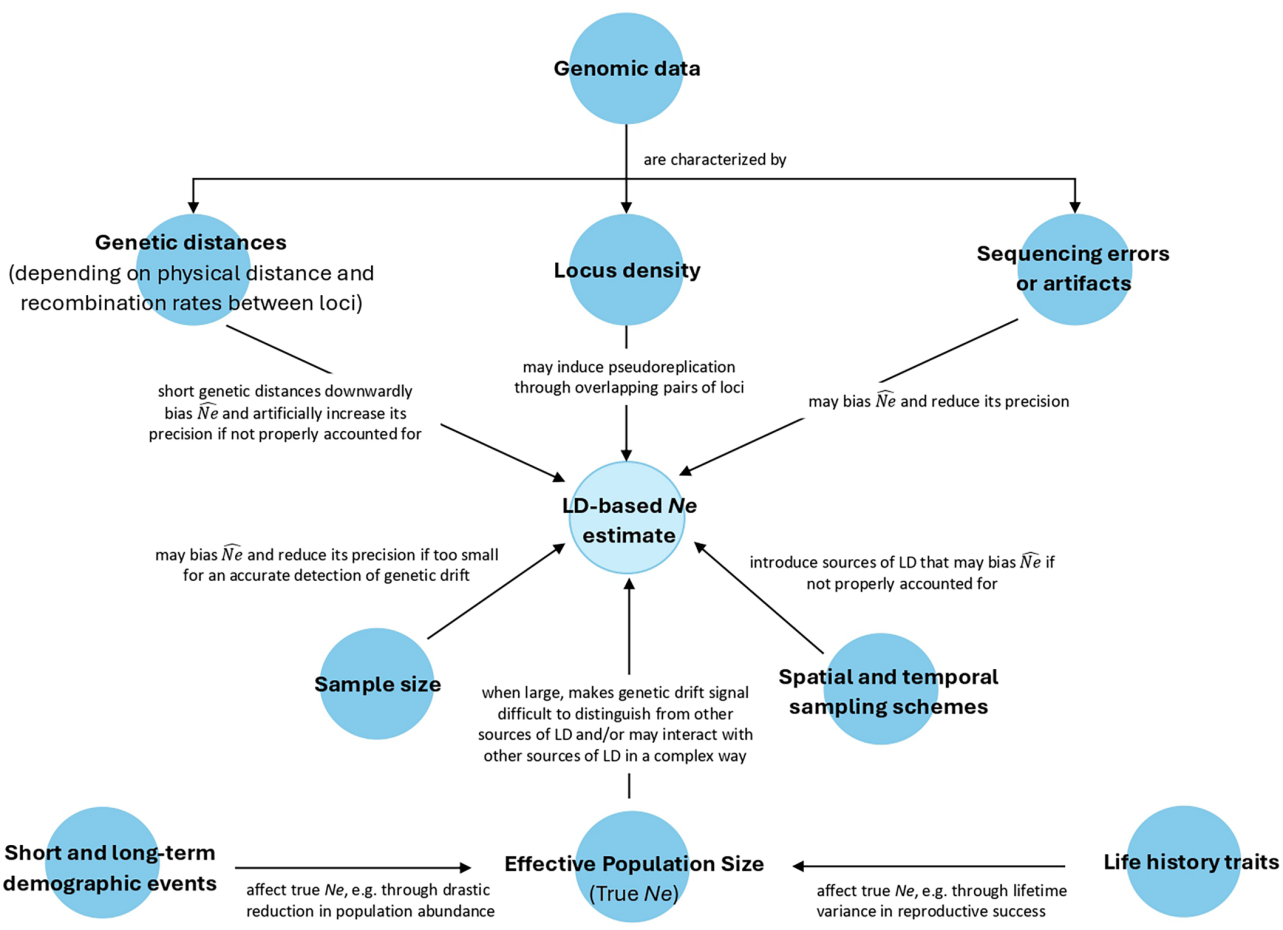
Summarised relationships between the key factors mentioned in this article and their influence on *Ne* estimation using linkage disequilibrium‐based methods and high‐density genomic data.

LD‐based methods for contemporary *Ne* estimation originally rely mainly on decomposing linkage disequilibrium into three potential sources: recombination, systematic sampling bias and genetic drift (see Appendix S1 for details about the general principles and software implementation of those methods). However, numerous additional factors can affect *Ne* estimates (Figure 1), as thoroughly detailed in several reviews (Waples 2024b). These include overlapping generations, gene flow, mating systems (e.g., lifetime monogamy or partially selfing populations) and changes in abundance over time, selection, and technical artefacts such as null alleles, genotyping errors, missing data and user locus selection when building data sets. Recommendations to avoid or mitigate these biases have been proposed in the literature, as summarised in Appendix S1, which reports key concerns raised by the scientific community and provides examples of studies addressing these sources of bias (Table A1.1: Appendix S1).

Importantly, correcting for finite sample sizes and systematic sampling bias (which occurs when close relatives are sampled at higher rates than they occur in the population as a whole, Waples 2024b), which influence the intensity of the genetic drift signal, is crucial for accurate *Ne* inference especially in large populations where the genetic drift is harder to distinguish from the influences of other factors and sample size is generally much lower than *Ne*. Existing corrections (Waples 2006; Sved et al. 2013) seem to be accepted by the scientific community, although further work is needed to adapt them to phased data sets (Saura et al. 2015; Beaumont and Wang 2019); however, for large pelagic species, reference genomes are limited, and phase information is often unavailable. Another important point is that most methods assume full independence of loci (Waples 2006) or at least known recombination parameters (Hill 1981), yet physical linkage can bias *Ne* estimates, particularly in high‐density data sets. Besides, although genetic and genomic methods based on LD are theoretically promising for estimating *Ne* in fisheries management, LD‐based methods remain rarely applied in fisheries and are more often discussed for their limitations than their benefits (Ovenden et al. 2016; Waples, Hoelzel, et al. 2018; Waples 2024b). The low intensity of genetic drift in large populations makes its effect on linkage disequilibrium difficult to detect, often overshadowed by systematic sampling bias, particularly with small sample sizes (Figure 1). As a result, LD‐based *Ne* estimates can be highly uncertain and rarely reflect true values, frequently showing a bimodal distribution with arbitrarily high, infinite, low or even negative—and thus unreliable—estimates (Macbeth et al. 2013; Ovenden et al. 2016; Waples 2016). For instance, Wang et al. (2016) reported that LDNe performance decreased markedly when sample size fell below 1.5% of the true *Ne*, even under ideal conditions (i.e., a Wright–Fisher population with *Ne* > 30,000 and 800 independent biallelic loci). Similarly, Marandel et al. (2019) recommended sampling a minimum of 1% of the total abundance (corresponding, in their study, to 0.87% of the true *Ne*), on the basis of simulations with *Ne* values reaching 1 million individuals and 200 biallelic loci. After reviewing a panel of 26 studies evaluating the effective sizes of marine species, the authors reported that almost all the studies used a sample size smaller than 1% of the assumed total abundance and yielded either infinite or negative *Ne* estimates. Macbeth et al. (2013) estimated that ~5000 individuals would be necessary to detect an *Ne* of ~30,000 and to accurately infer the lower bound of confidence interval for an *Ne* of ~60,000 individuals, implying that precise *Ne* estimation may require sampling ~16.7% of the true *Ne*. However, it is unclear whether this ideal ratio remains stable across different *Ne* values. This uncertainty is critical, as such large sample sizes pose significant technical, financial and computational challenges for conservation management.

The delay (‘time lag’) of detecting temporal fluctuations in *Ne* is a well‐known challenge in demography inference, especially for species of conservation interest (Ovenden et al. 2016). Antão et al. (2011) emphasised the need for sufficient samples to achieve unbiased estimation of contemporary *Ne* following a bottleneck, even in a simulated population with moderate *Ne* values (at most 2400) that decreases to 2% of its initial size. Similarly, Nunziata and Weisrock (2018) presented a simulation study of high‐throughput sequencing data, demonstrating the significant influence of the sample size on the ability of the method implemented in LDNe software to estimate the contemporary *Ne* of a declining population (with simulated *Ne* values of up to 1000). We might expect this time‐lag to be more important for large populations, even if they undergo fluctuations of similar magnitude.

More generally, it remains challenging to account for the influences of other biological factors that generate linkage disequilibrium, such as long‐term *Ne* values (historical averages of the effective population size over multiple generations), introgression and genetic substructure in large populations. For example, it is a complex task to precisely evaluate the bias induced by a small unrecognized genetic structure and to determine how the intensity of this bias changes relative to the true *Ne* of the population. In other words, a better understanding of the interaction between genetic drift and genetic substructure and its effect on the linkage disequilibrium signature is needed, particularly for large populations in cases where the substructure itself is difficult to detect (Bailleul et al. 2018). An exploration of these areas could build upon previous work. For example, Gilbert and Whitlock (2015) tested the influence of variable migration levels on *Ne* estimation via the method implemented in LDNe software, with simulated *Ne* values of up to 500 per sub‐population. Their findings revealed that the method had greater difficulty in estimating the highest local *Ne* values when gene flow was significant (and therefore the substructure was weak) between sub‐populations.

The use of high‐density marker data sets holds promise for improving the estimation of *Ne* in large populations. The significant increase in the number of loci studied favours their informativeness and a better detection of the evolutionary processes occurring within populations (e.g., greater detectability of spatial genetic structure, as discussed in Bailleul et al. 2018; Nikolic et al. 2023; Leone et al. 2024). It has already facilitated the development or deepening of entirely new mathematical methods for estimating *Ne* on the basis of linkage disequilibrium by incorporating recombination information between loci (Santiago et al. 2020). As such, we may wonder to which extent the use of a large number of loci could partially offset the technical limitations associated with collecting and processing many individuals. The use of such high‐density marker data sets, however, has its own challenges (Figure 1).

For example, the classical linkage disequilibrium‐based method for contemporary *Ne* estimation (such as that implemented in LDNe software, Waples and Do 2008) assumes that all loci are independent and consider physical linkage between loci as negligible. While this assumption may hold for data sets with tens to hundreds of markers, it becomes increasingly untenable with thousands or tens of thousands. In such cases, both the number of loci and their genomic arrangement influence the extent of physical linkage. For a given number of uniformly sampled loci, species with smaller genomes and/or fewer chromosomes are more prone to physical linkage than those with larger genomes or more chromosomes (although recombination rates and their variation along the genome also need to be considered). In such case, pseudo‐replication may occur as the number of truly informative loci (‘effective number of loci’) is actually lower than the number of loci in the data set due to their finite genetic distance (Waples et al. 2022). This pseudo‐replication results in an artificial increase in the precision of estimates (i.e., leading to narrower confidence intervals than expected if the markers were completely independent) and also increases the likelihood that these intervals do not contain the true *Ne* value. When genomic data sets are applied to large populations, the risk is that linkage disequilibrium caused by physical linkage between loci will become greater than that caused by genetic drift, resulting in negatively biased estimates of *Ne*. As an example, using linkage disequilibrium information obtained from pairwise comparisons of 78,636 SNPs in a passerine species with high abundance and a wide range, Nadachowska‐Brzyska et al. (2021) obtained a finite *Ne* value exceeding 23,000, an estimate that became negative (with infinite confidence intervals) when based solely on comparing pairs of loci from different chromosomes (i.e., fully independent loci). This suggests that the effective size is too large to evaluate despite the large number of loci used, and it underlines the potential negative bias caused by physical linkage between loci when all SNPs are considered. Waples et al. (2016) proposed a bias correction procedure in the absence of available recombination maps, relying on simulations with varying numbers of independent chromosomes (2–64) of diverse sizes (50, 100 or 200 cM), a number of loci up to 4096, and simulated *Ne* values up to 800. This correction has the form of a fairly simple equation but requires knowledge of the number of chromosomes or the total genome size of the target species. This correction must be applied with caution, ideally to data sets corresponding to the parameter space simulated by the authors. Its effectiveness in the case of *Ne* values much larger than a few hundred remains unknown. Indeed, although the effect of physical linkage between loci may become predominant in large populations compared with the effect of genetic drift, it can also be influenced antagonistically by *Ne* itself. Larger effective sizes accelerate the decline in linkage disequilibrium (LD decay) between loci for a given recombination distance (Waples et al. 2022).

Another source of pseudo‐replication in high‐density data sets results from overlapping pairs of loci: linkage disequilibrium values obtained between pairs of loci are not independent of each other, because each locus is involved in multiple comparisons. As with physical linkage between loci, this leads to an information gain (i.e., through the number of effective locus pairs, Waples et al. 2022) that does not increase as rapidly as the number of loci used for estimating *Ne*. Instead, the number of effective locus pairs as a function of the number of used loci eventually reaches an asymptotic value for a given combination of *Ne* and sample size (i.e., fig. 2 of Waples et al. 2022). For instance, in their simulations, Marandel et al. (2019) found no further information gain beyond 200 SNPs. According to Waples et al. (2022), this type of pseudo‐replication does not bias *Ne* estimates like physical linkage but leads to overestimated precision, with narrower confidence intervals less likely to include the true *Ne*. The jackknife method by Jones et al. (2016) is widely recommended to correct this issue, although its performance decreases when the sample size is small compared to the true *Ne* value. Again, this pseudo‐replication seems to diminish as *Ne* increases, with the proportion of effective locus pairs over the actual number of loci increasing as *Ne* tends towards infinity (see fig. 2 of Waples et al. 2022). Increasing sample size could also compensate for pseudo‐replication resulting from overlapping pairs of loci, providing it is close enough to *Ne* (i.e., fig. 3 of Waples et al. 2022) otherwise the information gain remains limited as well.

Finally, increasing the number of loci to improve contemporary *Ne* estimation is hampered by individual sampling and the sample size per se. This is because the degree of uncertainty related to individual sampling within a population largely outweighs the degree of uncertainty related to locus sampling, especially for large number of loci (i.e., fig. 4 of Waples et al. 2022). In fact, as stated by Waples (2024b), ‘as more SNPs are used, the estimate of [a linkage disequilibrium statistic] will converge on a value that reflects the relationship structure of the sampled individuals and not the population as a whole’.

The considerations above underscore the high importance of sample size and emphasise caution against allocating significant financial and logistical resources to sequencing and genotyping thousands or tens of thousands of loci in hopes of addressing limited sampling. These issues have been covered in the literature (Wang 2016; Waples et al. 2016, 2022) and are likely to be further explored in the future to answer outstanding questions about whether and how locus density compensates for a limited sample size.

Several recently developed methods based on linkage disequilibrium benefit from increasing genomic information. First, the availability of at least a draft reference genome for the species itself or a closely related species can provide information on the relative positioning of some characterized loci, enabling comparisons to be restricted between different chromosomes to avoid physical linkage. Additionally, a recombination map allows information to be gathered from physical linkages between loci to investigate changes in *Ne* over time. Such information may soon become increasingly available for large pelagic species. For example, the European Reference Genome Atlas (ERGA) project (https://www.erga‐biodiversity.eu/) aims to sequence the genomes of several European eukaryotic species, and for its pilot phase, among several aquatic and marine species, the blue shark ( *Prionace glauca* ) and the Atlantic bluefin tuna ( *Thunnus thynnus* ) have been selected. Linkage disequilibrium methods that integrate recombination information are undergoing constant optimisation and application. Some of these (SNEP, Barbato et al. 2015; LINKNE, Hollenbeck et al. 2016; moments‐LD, Ragsdale and Gravel 2019, 2020; Jouganous et al. 2017; GONE, Santiago et al. 2020) are described in Appendix S2. Overall, some methods perform better at obtaining contemporary *Ne* estimates, whereas others better detect the timing or strength of a recent bottleneck. Most applications of these methods seemingly concern vulnerable species of small abundance, but there are examples involving marine species with potentially large populations, as shown in Table A1.1: Appendix S1.

Thus, the limitations due to low genetic drift signals in large populations may persist even with the most recently developed methods. Among the applications of the methods presented here (see Table A1.1: Appendix S1), few cases result in the estimation of effective sizes greater than a few thousand. The performance of methods incorporating recombination information is likely influenced by relative fluctuations in the effective size between the recent past and the present, as mentioned by Lehnert et al. (2019) and suggested by the results of Martinez et al. (2022) (Appendix S1). It is thus unclear whether methods leveraging recombination information enable (i) the correct and precise evaluation of contemporary *Ne* even when it is high, (ii) the correct and precise evaluation of both contemporary and past fluctuations in *Ne* or (iii) a simply qualitative detection of recent change of *Ne* (i.e., identifying the occurrence of a decline or an expansion).

Following this synthesis, in light of recent reviews (e.g., Waples 2024b), we outline a few questions that appear to be compulsory when running conservation genetic programs focusing on large marine populations:

- Which correction procedure would most effectively eliminate systematic sampling bias when estimating *Ne* from a large population using linkage disequilibrium methods implemented in different software programs?
- In which specific ways do pseudo‐replication and sampling issues arise in populations with very large *Ne* when using high‐density SNP data, and which consequences are there in the minimal sample size necessary to obtain a reliable *Ne* estimation using linkage disequilibrium‐based methods (assuming the sample pedigree is representative of the full population pedigree)?
- Does the correction for systematic bias caused by physical linkage, as proposed by Waples et al. (2016, equations 1a and 1b) for contemporary *Ne*, remain valid for large *Ne* values and/or a very large number of genetic markers, such as those obtained through whole‐genome sequencing?
- How does the influence of ‘pseudo‐replication’ on the accuracy and precision of contemporary *Ne* estimates, using classical methods (Waples 2006) or more recently developed linkage disequilibrium‐based methods, vary with the true *Ne* value?
- How do the nature (constant vs. pulsed, symmetric vs. asymmetric, etc.) and intensity of gene flow affect the bias and precision of *Ne* estimates in large populations? What are the implications for methods that integrate multipopulation information, such as the moments‐LD method developed by Ragsdale et al. (2020)? How do the nature (e.g., gradual vs. sudden, expansion vs. decline), timing (time lag) and intensity of fluctuating *Ne* affect the bias and precision of contemporary *Ne* estimation in large populations?

## 3 Effective Size Estimation Based on Allele Frequency Spectra

The allele frequency spectrum (or site‐frequency spectrum, SFS) describes allele distributions in a population. The distribution of allele frequencies, and thus the characteristics of the SFS, reflects demographic factors such as changes in *Ne*, genetic differentiation between populations, population speciation processes and gene flow that have shaped populations throughout their history. Using mathematical models, it is possible to predict the expected characteristics of the SFS (or joint SFS when more than one population is considered) that would theoretically be observed under different demographic models. Demographic inference can then be performed by comparing the observed SFS (or JSFS for joint site frequency spectrum if more than one population are involved) to one or more theoretical SFS via statistical tools for maximum likelihood estimation or approximate Bayesian computation (Beaumont et al. 2002). Salmona et al. (2017) and Bourgeois and Warren (2021) provided a detailed review of the various existing demographic inference methods, particularly those based on the SFS (see also Appendix S2 for details about the general principles and software implementation of several SFS‐based methods).

The estimation of the demographic parameters of one or more populations primarily depends on (i) the informativeness of the SFS derived from empirical data, (ii) how realistic the demographic scenarios and the range of parameters tested are and (iii) the ability to correctly model the theoretical SFS for these different scenarios.

Demographic inference based on the SFS is generally most effective for past or moderately ancient events; recent changes in population size often leave weaker signatures, as recombination information cannot be considered (Hayes et al. 2003; Salmona et al. 2017) and the signatures of recent events on the SFS are diluted by the cumulative footprints of older events (Gattepaille et al. 2013; Nunziata and Weisrock 2018; Momigliano et al. 2021; Reid and Pinsky 2022). In fact, Reid and Pinsky (2022) reported a higher efficiency of momi2 (based on a continuous‐time Moran model) compared with that of the stairway plot (based on the coalescent) and two linkage disequilibrium‐based methods (NeEstimator2 and GONE), provided that the demographic decline was fast and older than 30 generations. For more recent events, the linkage disequilibrium method, which integrates recombination information across the genome (GONE) performed better. The authors also suggested that SFS‐based methods are more precise and less dependent on sample size for estimating long‐term *Ne* values, whereas GONE software is more effective at assessing *Ne* values from recent timescales with quality and precision, although it is more affected by sample size. Beichman et al. (2018) recommend a minimum of 100 individuals for studying events occurring within the last 100 generations using RADseq‐type sequencing data. Similarly, Robinson et al. (2014) suggested, through simulations, that while a few individuals and several thousand SNPs may suffice to detect demographic trends since the Upper Pleistocene with the δaδi software, several tens or even thousands of individuals (as in the case of global human demographic expansion; Keinan and Clark 2012) are needed to date more recent abundance expansion or declining events. Large populations, which are characterised by slower changes in allele frequencies (Hare et al. 2011; Hoey et al. 2022), present additional challenges, particularly when attempting to detect recent impacts. This issue is especially relevant for species of management or conservation concerns due to anthropogenic pressures, which often occur at recent timescales, such as exploited marine species (Puncher et al. 2018; Waples, Hoelzel, et al. 2018; Waples, Grewe, et al. 2018; Nikolic et al. 2023; Leone et al. 2024). Consistent with these observations, skyline plot tools have successfully detected Pleistocene events in the large pelagic blue shark *P. glauca* using mitochondrial DNA (Leone et al. 2017). However, despite relying on a thousand SNPs, the DarTSeq data derived from several tens of individuals failed to identify demographic events suspected to have occurred on very recent timescales (< 20 generations) in both *P. glauca* (Nikolic et al. 2023) and in albacore tuna (Nikolic et al. unpublished data). A more recently developed method, designed specifically to detect recent demographic events with relatively limited sample sizes, can provide valuable insights when applied to abundant pelagic species, provided that a recombination map is available (Tournebize et al. 2022). In contrast, for populations of small size (*Ne* up to 1000), Nunziata and Weisrock (2018) suggested prioritising the number of SNP markers (up to 25,000–50,000) over the number of samples in detecting a recent drastic (90%) decline using fastSimcoal2 software. However, the authors highlighted a systematic overestimation of *Ne*, which may be inherent to situations where the sample size is comparable to the effective population size under study (Bhaskar et al. 2014). This overestimation may be due to the underestimation of singletons when using the coalescent relative to estimation by simulation tools based on the Wright–Fisher model.

Stringent filtration, even at the expense of the number of usable SNPs, is required to increase the reliability and precision of inferences, as it has been shown that the most informative loci in the context of demographic inferences (i.e., those bearing rare alleles) are more strongly affected by allele dropout (Nunziata and Weisrock 2018). Notably, null alleles are very difficult to avoid (Hoey et al. 2022) and tend to be more prevalent in large populations (Gautier et al. 2013). Some methods have been developed to reconstruct the observed SFS while accounting for quality variation and low sequencing coverage (Korneliussen et al. 2014).

Temporal sampling mitigates some biases, allowing better estimation of *Ne* over recent generations. This was exemplified in Hoey et al. (2022) through the use of fastSimcoal2 on 1068 SNP markers and several tens of samples (26–150 genotyped larvae) per time step for three distinct cohorts (1994, 1997 and 2008) of the highly abundant demersal Summer flounder ( *Paralichthys dentatus* ). The results obtained were consistent with stock assessments reporting a drastic decline (~98%) in the population less than 20 generations earlier, followed by recovery. Their work highlights the interest of temporal sampling to infer recent demographic events in highly abundant populations using a few tens of individuals with relatively few but very high‐quality SNP markers (filtered through very strict data sequencing protocols) and a relatively simple model. Similarly, the work of Reid and Pinsky (2022) mentioned earlier underscores the value of temporal sampling in limiting the influence of allele dropout and the artefactual introduction of singletons when using momi2 and a stairway plot.

It is thus clear that increasing sample sizes, ideally at different time steps, and marker density is necessary for constructing empirical SFS for large pelagic populations to infer recent *Ne* fluctuations. Even with these optimal designs, logistical constraints remain significant, particularly owing to the increasing computational demands of the methods discussed here as model complexity and parameter ranges increase. Prior knowledge of the biology and recent history of target populations is therefore essential for constraining the parameters and testing optimal scenarios.

A major issue, known as ‘model identifiability’, arises from distinct evolutionary trajectories that may result in highly similar SFS (Myers et al. 2008; Momigliano et al. 2021). The amount of information contained in the SFS affects both (i) the ability to confidently identify the best theoretical scenarios corresponding to the observed SFS and (ii) the uncertainties (confidence intervals) associated with the estimation of the demographic parameters themselves.

Above, we focused on the difficulty of identifying recent changes in *Ne* of an isolated population, particularly when it is highly abundant. Estimating other demographic parameters (e.g., the intensity and direction of gene flow and the divergence time between multiple sub‐populations) can also be challenging, especially for complex evolutionary trajectories involving multiple populations and multiple demographic events over time. Accurate inference requires the testing of realistic demographic models that reflect the biological reality of these populations (Loog 2021). Limited prior knowledge can result in underfitting or the neglect of key scenarios. In such cases, it is necessary to report all selected theoretical scenarios identified through inference, noting that they are all equally likely to reflect the actual evolutionary trajectory of the population, which remains impossible to determine.

Biological data from the literature or the study of genetic variability within the populations of interest can help identify the broad categories of demographic scenarios to be tested for inference, for example, by identifying geographically distinct sub‐populations potentially resulting from a divergence event in their history (Nikolic et al. 2023). In the case of large pelagic species, a limited number of sub‐populations originating from a single ancestral population are generally considered and are strongly connected to each other by gene flow (Nikolic et al. 2023). Although this may constrain the number of demographic models to consider, the typically wide distribution range of these species usually leads to other challenges. Elucidating the phylogeographic patterns of these species may still require the integration of different modalities of divergence, secondary contact, admixture, gene flow directions and variations in effective population sizes. In addition, sampling may be biased because of unsampled (‘ghost’) populations. For example, in the case of blue shark *P. glauca* , Nikolic et al. (2023) suggested that the availability of individuals collected in the Pacific Ocean would have allowed for a better understanding of the precise gene flow patterns on a global scale. Along with the large number of theoretical scenarios to be tested, there may be an increase in their complexity and in the number of demographic parameters to be estimated within each scenario. If one is primarily interested in estimating contemporary *Ne*, it remains useful—and often important—to elucidate the past effective size variations and date them to limit parameter estimation biases. For example, Momigliano et al. (2021) showed how, during an inference procedure, not accounting for ancient fluctuations in *Ne* can lead to underestimating the times and modes of divergence between the studied populations. Demographic parameters (such as divergence times) of populations that are strongly connected to each other or show continuous genetic structuring can also be more difficult to estimate independently (Loog 2021). Lesturgie et al. (2022) demonstrated, using coalescent‐based inference procedures on four shark species with varying degrees of vagility, that neglecting spatial genetic structuring, particularly when it is strong, can make it difficult to distinguish the signature of a recent decline in *Ne* from that of spatial genetic structuring.

Demographic inference software based on coalescent methods, such as fastSimcoal2, enables the development of many distinct demographic models with diverse parameters that can be compared using statistical tools such as the Akaike information criterion (AIC) on independent molecular markers. However, a very large number of simulations is required to cover all possible combinations of the ‘parameter space’, and if the empirical SFS is too uninformative, the range of uncertainty around these parameters remains large. Moreover, the classic coalescent model assumes that multiple coalescent events are impossible (only one coalescent event, between two gene copies only, can occur each generation). This assumption may not hold in several cases: (i) when the variance in reproductive success within the studied population is so large that multiple coalescent events can occur simultaneously (Montano 2016), (ii) when the population has undergone an extreme decline over a short and recent period, concentrating many coalescent events over a small number of generations (Lauterbur 2019) and (iii) when the sample size is comparable to the true *Ne* (Bhaskar et al. 2014). If any of these situations is likely, the use of dedicated coalescent models, known as ‘multiple mergers’, is recommended (Tellier and Lemaire 2014). These models are implemented in simulation software such as *msprime* (Kelleher et al. 2016) and MetaGeneTree (Birkner et al. 2011), but not in fastSimcoal2, for example, which can handle such situations only in the specific case of simulating an instantaneous bottleneck. In these cases, there is a risk that the simulated genetic variation patterns within the framework of inference will be biased, especially for recent generations (Bhaskar et al. 2014). Rare alleles are generally the most affected, leading to an underestimation of the number of singletons and an overestimation of the number of doubletons by classic coalescence models. Linkage disequilibrium patterns along the genome can also be biased when long genomic regions are simulated (Nelson et al. 2020), potentially influencing demographic parameter estimates based on this information.

For populations of conservation interest, the occurrence of strong bottlenecks and variance in reproductive success cannot clearly be ruled out, and multiple merging events must be considered. Using a classic Wright–Fisher model to obtain a theoretical SFS, as implemented in software such as δaδi and moments, circumvents this issue. However, this approach is more restrictive in terms of the diversity of models and the range of parameters that can be tested. GADMA and GADMA2 software (Noskova et al. 2020, 2023) yields interesting prospects for combining the precision and accuracy of methods on the basis of the exact calculation of SFS by comparing distinct demographic models to explore a larger parameter space. Like GONE software, GADMA implements a genetic algorithm that allows the automatic generation and gradual refinement of different scenarios over several successive ‘generations’. GADMA is related to some of the existing software mentioned above (δaδi, moments, moments.LD and momi2) and enables the pruning of the number and range of demographic parameters to be tested and optimised. Such an approach may help limit biases and avoid underfitting demographic scenarios relative to the biological reality of the target populations (Momigliano et al. 2021).

To summarise, SFS‐based methods can help estimate the effective population size. However, to improve their use and reliability, particularly for highly abundant large pelagic populations, key questions need to be addressed, including the following:

- Can demographic inference methods based on SFS that integrate a genetic algorithm (e.g., GADMA) optimise the selection of demographic models and the estimation of parameters such as effective size (and its temporal variations) for abundant, interconnected populations?
- How many samples and loci are needed for SFS to be used to detect recent *Ne* declines in large populations with constant, asymmetric and/or pulsed gene flow?
- What are the relative performance levels of different algorithms (e.g., diffusion equations, continuous‐time Moran models, coalescents) in estimating *Ne* (and its temporal variations)?
- How does *Ne* influence the ability to independently estimate demographic parameters such as migration, divergence time and past variations in effective size?

## 4 A Simulation Framework for High‐Density Genomic Data and Large Sample Sizes

### 4.1 Preliminary Considerations for Several Simulation Software Programs

Based on the previous sections, we outline here some key requirements a simulation framework should meet to reliably compare *Ne* estimation methods in large populations with high‐density SNP data:

- Simulate realistic populations, which requires accounting for individual‐level variation in vital rates, particularly survival and fertility, which can significantly influence *Ne* and its estimation. It is also essential to model populations over sufficiently long time periods to capture both short‐term demographic features (such as lifetime‐variance in reproductive success across individuals) and long‐term evolutionary trajectories (such as progressive growth or decline or past divergence events within and between populations). This calls for the use of individual‐based simulators, which can incorporate these complex dynamics, as opposed to traditional coalescent‐based simulators that rely on simplifying assumptions, such as non‐overlapping generations and equal reproductive probabilities across individuals.
- Simulate genomic data with at least a few thousand independent loci, and when possible, generate complementary information such as recombination distances between loci.
- Preserve pedigree information between individuals to determine their relatedness within a simulated sample and to determine how the number of related pairs of individuals evolves under different conditions or sampling schema.
- Establish a diagnosis of the simulated data to ensure that the simulated genetic and demographic features align with theoretical expectations. For example, it is important to confirm that the effective size of the simulated population matches the value predicted by the set of chosen demographic parameters. If discrepancies arise, it is necessary to determine the actual simulated effective size to compare it with the estimates from the various methods being tested.
- Integrate both contemporary time (present and recent past) and more ancient time to simulate populations with realistic evolutionary trajectories. This is particularly important for evaluating effective size estimation methods on the basis of allele frequency spectra as well as for simulating realistic patterns of genetic diversity.
- Enable flexibility in varying multiple parameters, such as the total abundance and effective size, on the basis of different vital rates (survival and fertility rates), the number of sub‐populations, the intensity of gene flow between sub‐populations, sampling strategies (including serial sampling across multiple time steps), and genomic characteristics (e.g., recombination rates and genome sizes).

Multiple simulation software programs and tools are available, each with specific advantages. Table 2 lists some of the most widely used and maintained software that can both simulate demographic and genomic data for populations of potentially large abundance.

**TABLE 2:** Commonly used demo‐genetic simulation software programs listed with their main strengths and limitations in simulating genomic data for populations with very large effective population sizes.

Software	Resources	Strengths	Challenges
fastSimcoal	http://cmpg.unibe.ch/software/fastsimcoal26 https://groups.google.com/g/fastsimcoal?pli=1	Incorporates a highly flexible demographic inference tool based on site frequency spectra Provides extensive community support and bibliographies	Requires scripting for genotype format conversion (no genepop or .vcf format is available for direct output) Generates unexpected genetic diversity patterns beyond a certain population and sample size
msprime	https://tskit.dev/msprime/docs/stable/intro.html https://github.com/tskit‐dev/msprime	Speed Leverages the flexibility of the Python language Implements genealogy storage in tree sequences Includes numerous tools for analysing simulated data (*tskit* library) Extremely active community support, abundant online resources	Complex software requires significant investment for proficiency Does not integrate direct demographic inference tools (must be coupled with other tools for inference via ABC or likelihood maximisation)
SimuPOP	https://simupop.sourceforge.net/ https://github.com/BoPeng/simuPOP	Biologically realistic (individual‐centred), forward‐time simulation Benefits from the flexibility of the Python language Widely used, examples available back to the 2000s	Difficult to combine with coalescent‐based simulations for hybrid simulations Long simulation times
SLiM	https://messerlab.org/slim/ https://groups.google.com/g/slim‐discuss https://tskit.dev/pyslim/docs/stable/introduction.html	Biologically realistic (individual‐centred), forward‐time simulation Implements genealogy storage in tree sequences Allows coupling with coalescent for hybrid simulations (*pyslim* library) Flexibility	Complex software requires significant investment for proficiency Eidos programming, dedicated programming language Long simulation times in some cases
Spip/CKMRpop	Anderson (2022) https://eriqande.github.io/CKMRpop/index.html	Biologically realistic (individual‐centred), forward‐time simulation User‐friendly R interface and tutorials Users can easily set up various life cycles and sampling strategies Includes tools for describing pairs of related individuals within simulated data (e.g., classification by type of relatedness, triad detection, etc.) A valuable pedagogical tool for understanding the principles of the close‐kin mark recapture method (CKMR, Bravington et al. 2016) and for simple study design	Performance is limited beyond a few dozen loci; thus, simulation of genomic data is not possible Memory allocation issues when simulating multiple populations with migration and for certain population sizes Does not implement CKMR demographic model tools and equations: solely aims to visualise the number of related pairs present (R) based on the life cycle and sampling strategy Does not allow simulation of past evolutionary trajectories (contemporary time only)

Here, we present a simulation framework based on the individual‐based, forward‐time SLiM (Haller and Messer 2016, 2018) and coalescent (or discrete‐time Wright–Fisher)‐based *msprime* (Kelleher et al. 2016) software programs, along with the *pyslim* and *tskit* Python libraries. These population genetic simulators enable high flexibility for simulating biologically realistic populations and demography over recent time periods using individual‐based simulations, while also generating realistic patterns of genetic variation based on different scenarios of past evolutionary trajectories using coalescent models. Additionally, these tools implement a data encoding process that preserves the genealogical and genetic characteristics of individuals and populations in an optimized manner: tree sequence recording, improving simulation performance, which is particularly beneficial when simulating large populations and sample sizes.

### 4.2 Simulation of Demographic and Genomic Data via SLiM 3.7, *pyslim* 0.700 and *msprime* 1.2.0, Focusing on Parameters Characterising Large Pelagic Fish Populations (Such as Tuna)

**FIGURE 2:**
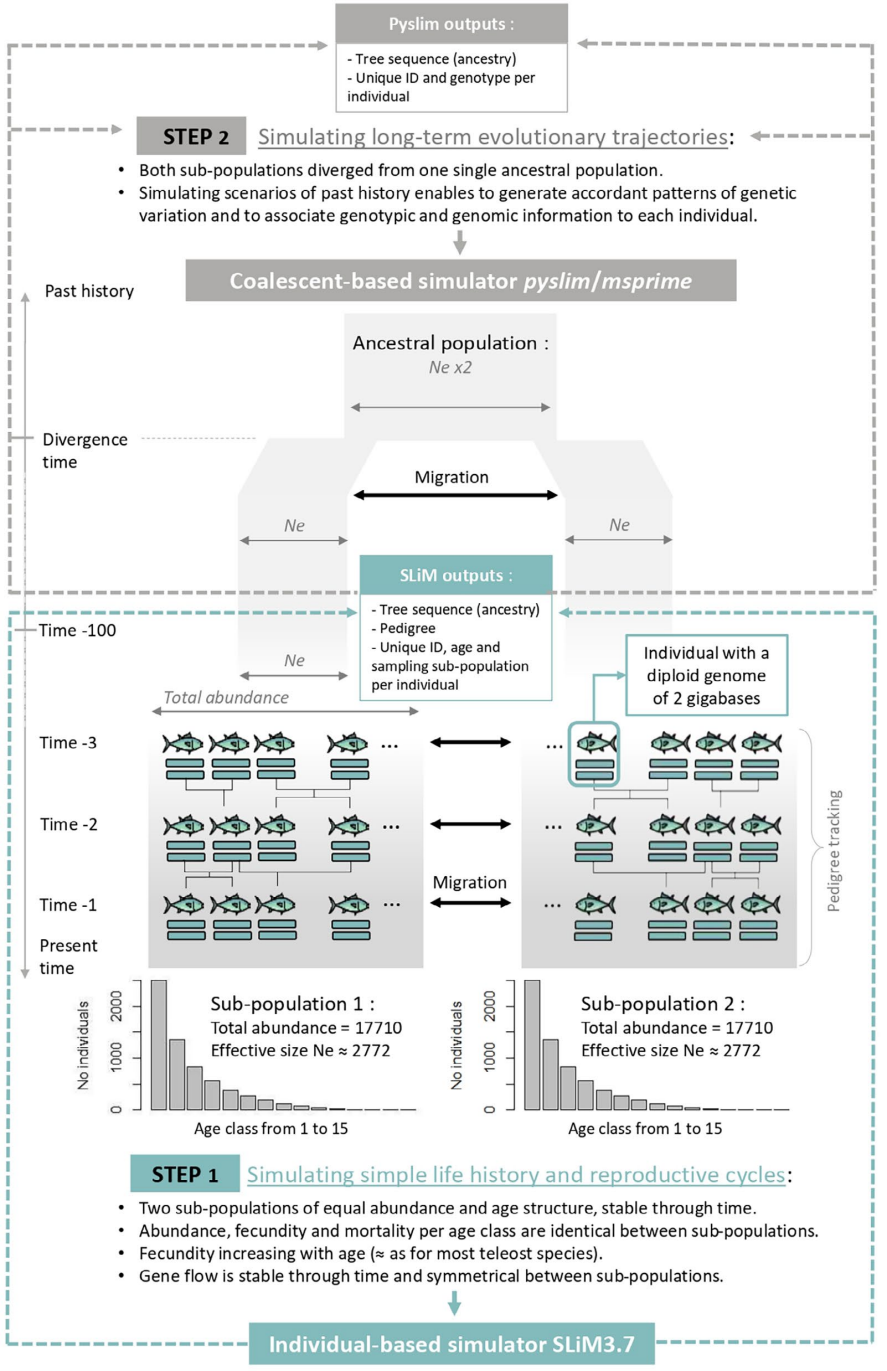
Overview of the procedure for simulating demographic and genomic data. This procedure is divided into two interrelated steps.

Our simulation procedure is summarised in Figure 2, with detailed information provided in Appendix S3 and on GitHub (https://github.com/[author]/POPSIZE‐Project‐SLiM_Scripts). Its objective is to propose a framework to simulate biologically realistic populations of moderate (a few thousands) to large (ideally up to millions of individuals) census size, and with vital rates that are to be set by the user so as to mimic any particular species (e.g., a tuna species). Here, as an example, we used simplified vital rates inspired by Nishida and Dhurmeea (2019) who present a review of demographic parameters for yellowfin tuna in the Indian Ocean stock.

Using SLiM version 3.7, we simulated two connected sub‐populations with identical total abundances ‘K’ (equal to 17,710 individuals in the example provided on GitHub), each generating the same number of newborns, ‘final_cohort_size’ (which was arbitrarily chosen to be of 5000 individuals and conditions the total number of individuals K), in each reproductive cycle. The simulated populations are age‐structured, with mortality rates L′ and fertility rates ‘B’ for each age class. The age of sexual maturity was set to 4 years, beyond which all individuals were capable of reproduction. Longevity was set to 15 years, beyond which all individuals perished. In this configuration, applying AGENE software (Waples et al. 2011), and assuming constant total abundance and age structure over time, the population size per age class decreased from 5000 individuals at age 0 (newborns) to 2 individuals at age 15. It was assumed that all newborns survived until age 1. This age structure parameter is integrated into the SLiM simulator as the parameter ‘W’. In this configuration, the theoretical demographic effective size, which was calculated by estimating the variance in reproductive success on the basis of fixed demographic parameters (Waples et al. 2011), was 3314 per generation for each sub‐population. In our situation, the observed demographic effective size, which was calculated on the basis of the variance in actual reproductive success among the simulated individuals in each cohort from one time step to another, averaged 2772 per generation. We hypothesise that the difference between the theoretical and observed values is due to stochastic processes associated with the SLiM simulator. It would be useful to explore this question further in the future. At this stage, however, we consider the value of 2772 to be the ‘true’ effective size for each sub‐population, the value that indirect estimation methods based on genetic information should be able to detect. The mortality parameters of each age class ‘L’ were inspired by Nishida and Dhurmeea (2019), who presented a review of demographic parameters for yellowfin tuna in the Indian Ocean stock, whereas the fertility parameter ‘B’ simply reflected a linear increase in the fertility rate with age, as is often expected in teleost fish. All the demographic parameters considered were identical between males and females, and we used a fixed, balanced sex ratio. Additionally, the demographic parameters were identical between the sub‐populations. The vital rates of each simulated sub‐population are summarised in Table A3.1: Appendix S3.

At each time step, breeding individuals generate a total of 5000 descendants on average and face a mortality risk depending on their age and the parameter ‘L’. Moreover, at each time step, each individual has a probability ‘m’ (fixed at 5% by default) of migrating from one of the two sub‐populations to the other. This migration parameter is constant over time and symmetrical, meaning that the two identically abundant populations exchange migrants in stable and equivalent proportions from one time step to the next. The simulation ran for 100 time steps. For each time step from 90 to 100, sampling was performed for each age class from 1 to 15 at a rate of 10% of the total abundance of individuals aged 1–15 in each class. Information regarding the age and location of sampling, pedigree and genetic genealogy of these individuals (using the tree sequence recording procedure) was exported for subsequent steps. Table A3.2: Appendix S3 summarises the key parameters set in the SLiM simulation phase.

At the end of the simulation phase conducted via SLiM software, we had a tree sequence file containing genealogical information throughout the genome of the sampled individuals between time steps 90 and 100. This was our starting point for the beginning of the simulation phase conducted via the *pyslim* and *msprime* libraries and starting with a process called recapitation (Appendix S3). During this coalescent phase of our framework, we simulated a maximally simplified evolutionary trajectory by simply merging the two sub‐populations into a single ancestral population (with an effective size equivalent to the sum of the effective sizes of the two sub‐populations). Table A3.3: Appendix S3 summarises the key parameters set in the coalescent phase of our simulation framework.

### 4.3 Examples of High‐Density Simulated Genotype Data

This section presents a small‐scale, illustrative simulation‐based comparison of methods for *Ne* estimation using the software NeEstimator2 (Do et al. 2014), GONE (Santiago et al. 2020) and GADMA with the library moments (Noskova et al. 2020). These methods were applied to 12 data sets generated through simulation and post‐processing procedures (details in Appendix S2 and GitHub). The simulations were conducted with a total sub‐population abundance (‘*K’* parameter) of 17,710 individuals. All parameters in the SLiM software were identical except for the gene flow ‘*m*’, which was set to 0.01, 0.05 or 0.10. For each ‘*m*’ value, three replicates were performed, resulting in nine independent simulations. Twelve subsets of data were generated per simulation, yielding 108 subsets of data, as summarised in Table A3.4: Appendix S3. Only samples from present time (time step 100) were kept in those subsets. Methods based on LD were applied to all 108 data sets, whereas GADMA software was applied only to the 36 subsets of data containing 30,000 loci. The parameters set for each of the three software programs are presented in Appendix S4.

The simulated sub‐population *Ne* ranged from 2740 to 2842 due to demographic stochasticity, with an average of 2772 considered the target value for comparison. The sampling size was set to 14 individuals (0.5% of the target *Ne*), 56 (2.0%), 140 (5.0%) and a ‘typical’ number of 50 individuals as a realistic field sampling scenario.

The estimates varied greatly between the sub‐populations and among the three replicates, regardless of the method used, the number of loci considered or the gene flow. Here, we present only the estimates and not their confidence intervals for better readability. The aim is not to draw definitive conclusions regarding the relative performance of the different methods; such a discussion requires a larger number of simulation replicates (ideally, replicates should be performed within each simulation, i.e., for each of the 12 subsets of data generated during the post‐processing phase of a given simulation) and larger‐scale simulations, including variations in the ‘*K*’ parameter indicating the abundance of each sub‐population.

#### 4.3.1 Influence of the Sample Size of Each Sub‐Population

**FIGURE 3:**
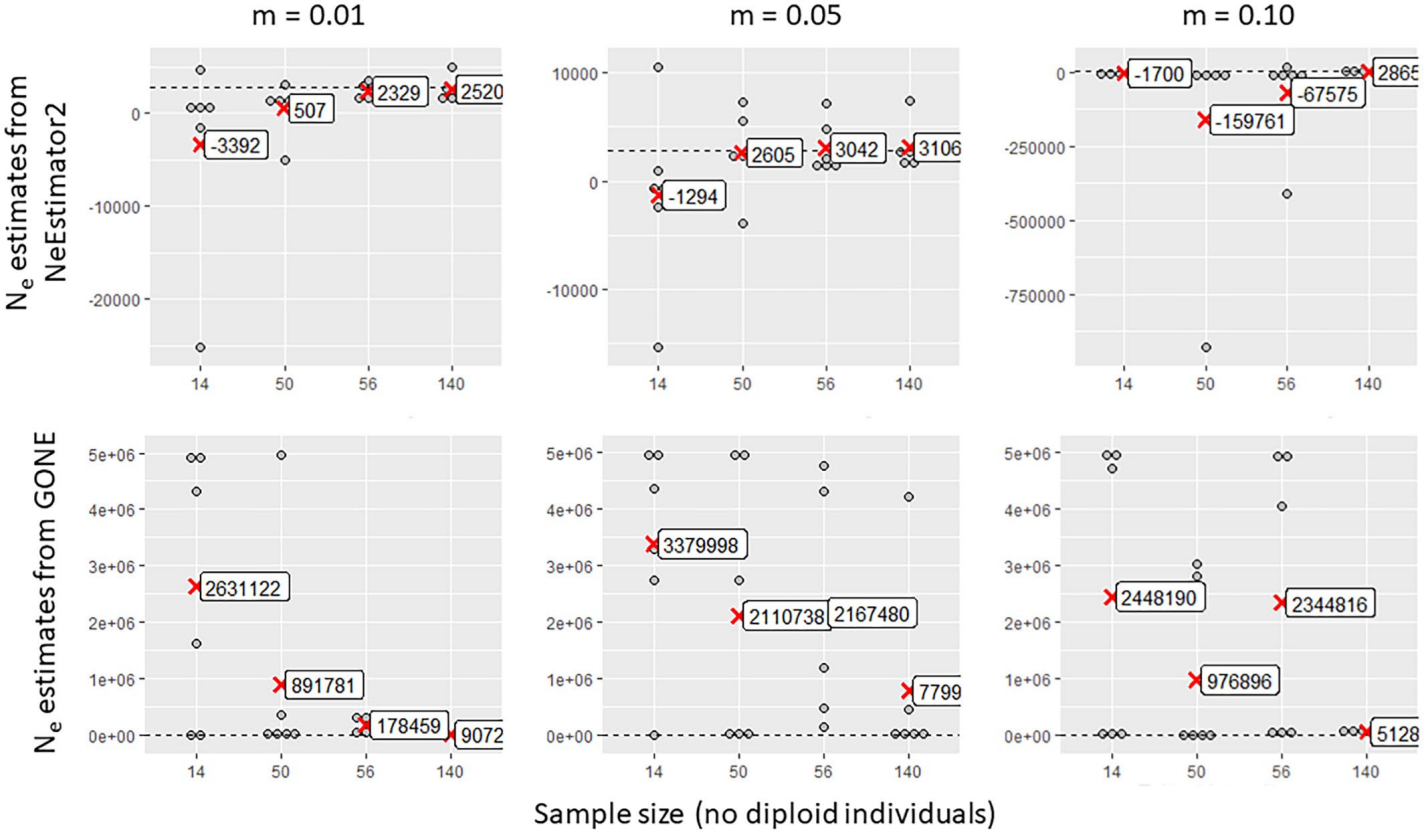
Estimated values of contemporary effective population size as a function of the number of samples collected per sub‐population (14, 50, 56 or 140), obtained by two linkage disequilibrium methods implemented in the software programs NeEstimator2 (top) and GONE (bottom); these methods are applied to simulated data sets with gene flow between sub‐populations of *m* = 0.01, 0.05 or 0.10 and with 1000 loci. Each point represents an estimate of the effective size of a sub‐population in one of the three simulation replicates. The mean effective population sizes within each sample size class are indicated by red crosses. The dashed horizontal line in each graph represents the target effective size (2772).

**FIGURE 4:**
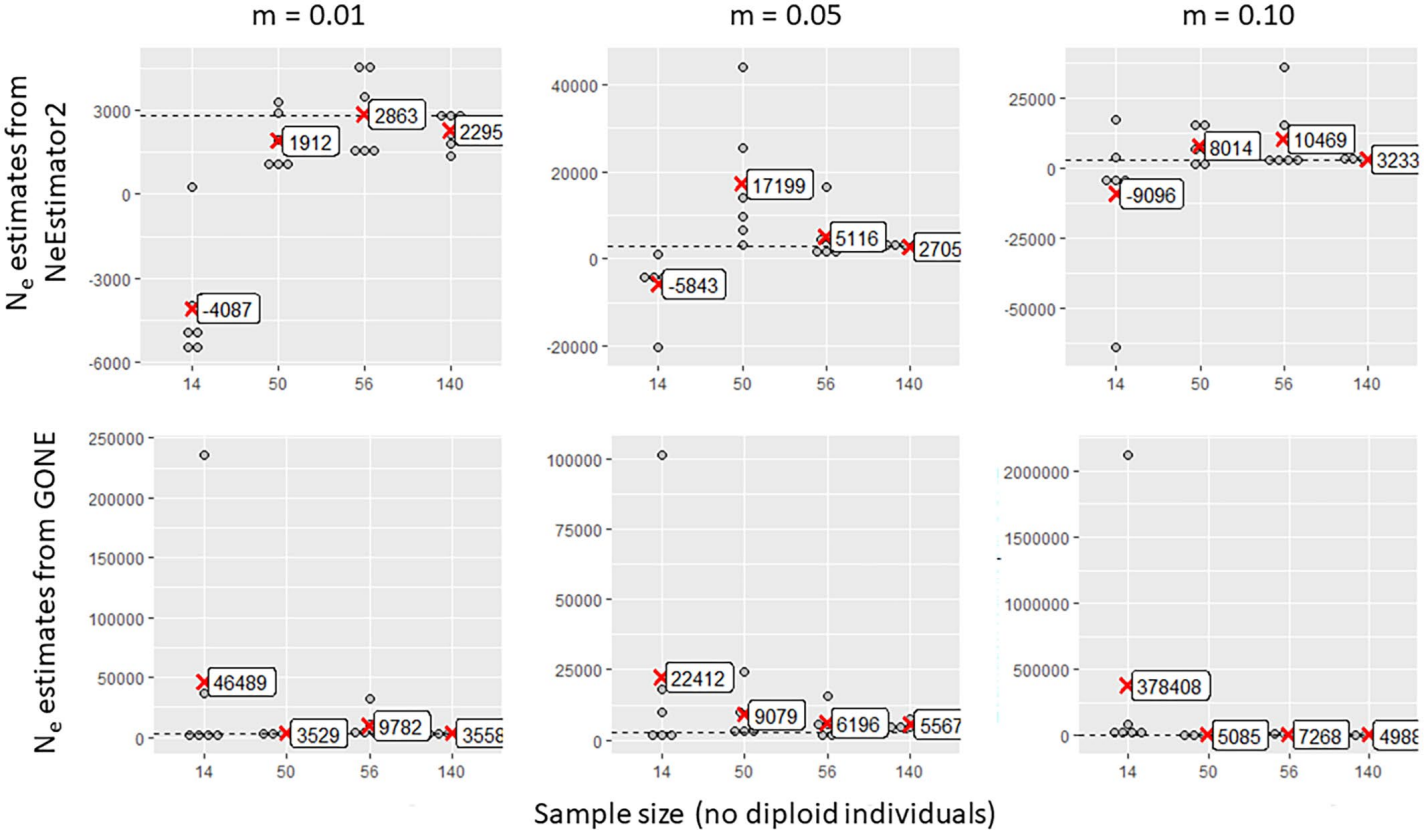
Estimated values of contemporary effective population size as a function of the number of samples collected per sub‐population (14, 50, 56 or 140), obtained by two linkage disequilibrium methods implemented in the software programs NeEstimator2 (top) and GONE (bottom); these methods are applied to simulated data sets with gene flow between sub‐populations of *m* = 0.01, 0.05 or 0.10 and with 10,000 loci. Each point represents an estimate of the effective size of a sub‐population in one of the three simulation replicates. The mean effective size values within each sample size class are indicated by red crosses. The dashed horizontal line in each graph represents the target effective size value (2772).

**FIGURE 5:**
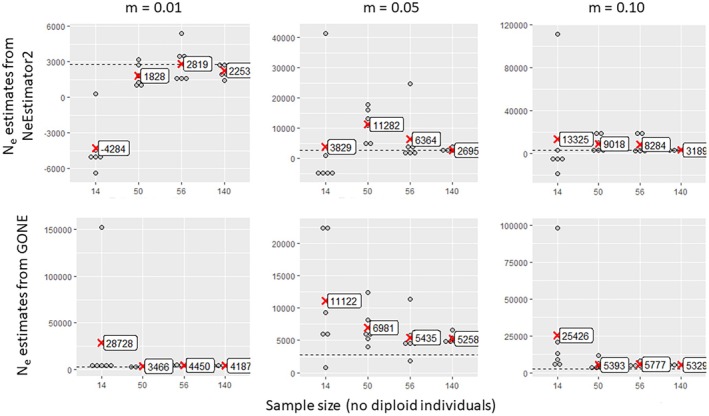
Estimated values of contemporary effective population size as a function of the number of samples collected per sub‐population (14, 50, 56 or 140), obtained by two linkage disequilibrium methods implemented in the software programs NeEstimator2 (top) and GONE (bottom); these methods are applied to simulated data sets with gene flow between sub‐populations of m = 0.01, 0.05 or 0.10 and with 30,000 loci. Each point represents an estimate of the effective size of a sub‐population in one of the three simulation replicates. The mean effective size values within each sample size class are indicated by red crosses. The dashed horizontal line in each graph represents the target effective size value (2772).

**FIGURE 6:**
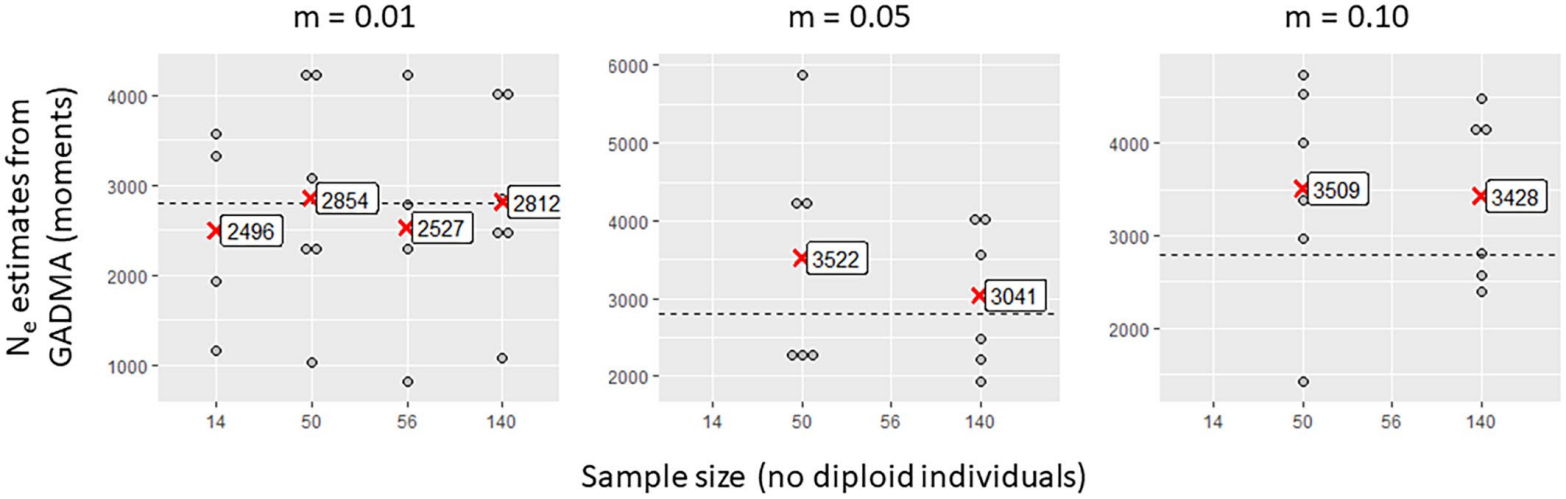
Estimated values of contemporary effective population size as a function of the number of samples collected per sub‐population (14, 50, 56 or 140), obtained by the allele frequency spectrum analysis method (method of moments) implemented in GADMA software, which was applied to simulated data sets with gene flow between sub‐populations of *m* = 0.01, 0.05 or 0.10 and with 30,000 loci. Each point represents an estimate of the effective size of a sub‐population in one of the three simulation replicates. The mean effective size values within each sample size class are indicated by red crosses. The dashed horizontal line in each graph represents the target effective size value (2772). For *m* values of 0.05 and 0.10, estimates were calculated only for sample sizes of 50 and 140 individuals per sub‐population owing to computational time constraints.

As expected, small sample sizes (14) produced unreliable and highly variable estimates across all methods (Figures 3, 4, 5, 6). Only GADMA software yielded a small range of variation, with a mean value relatively close to the target value (Figure 6). Larger sample sizes of 50, 56 or 140 individuals increased accuracy but showed variability in the estimates depending on migration and method. NEESTIMATOR2 was more accurate at low migration rates (0.01) and inconsistent at higher rates. GONE software showed less variation between estimates beyond 50 individuals and 10,000 loci but consistently overestimated *Ne* owing to sensitivity to gene flow even for the lowest gene flow value (0.01), for which NEESTIMATOR2 was generally less affected. This sensitivity to gene flow was previously reported by Santiago et al. (2020), who indicated that beyond a certain rate, local *Ne* estimates tend towards the overall *Ne* of their meta‐population. GADMA provided the closest estimates to the target *Ne*, particularly for 50+ individuals and 30,000 loci, although variability remained, with estimated values ranging from 1000 to 6000 and slight overestimation of the target *Ne* for higher gene flow (0.05 and 0.10).

#### 4.3.2 Influence of the Number of Loci

**FIGURE 7:**
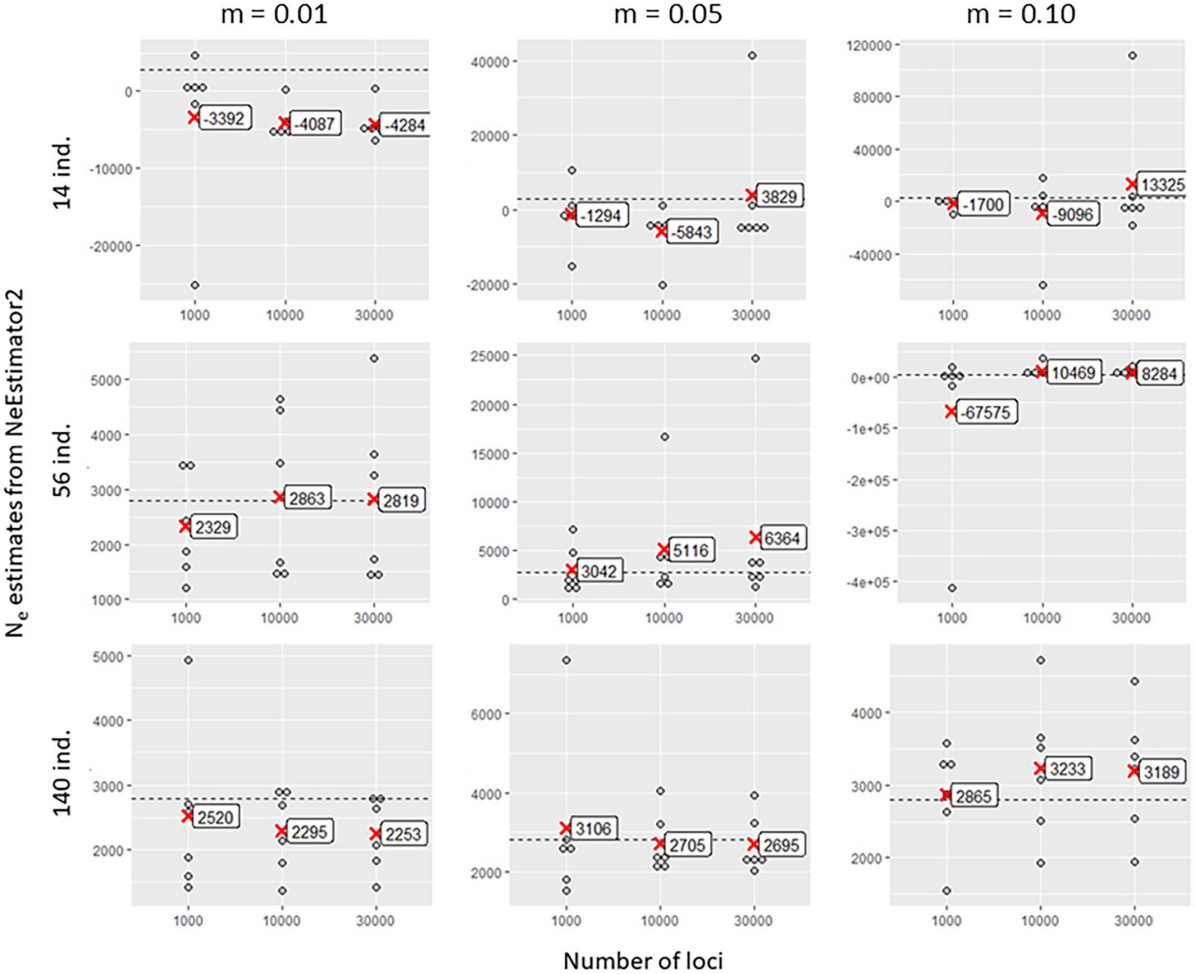
Estimated values of contemporary effective population size as a function of the number of loci involved in the analysis (1000, 10,000 or 30,000), obtained by the linkage disequilibrium method implemented in NeEstimator2 software, which was applied to simulated data sets with gene flow between sub‐populations of *m* = 0.01, 0.05 or 0.10 and with 14, 56 or 140 individuals per sub‐population. Each point represents an estimate of the effective size of a sub‐population in one of the three simulation replicates. The mean effective size values for each locus number class are indicated by red crosses. The dashed horizontal line in each graph represents the target effective size value (2772).

**FIGURE 8:**
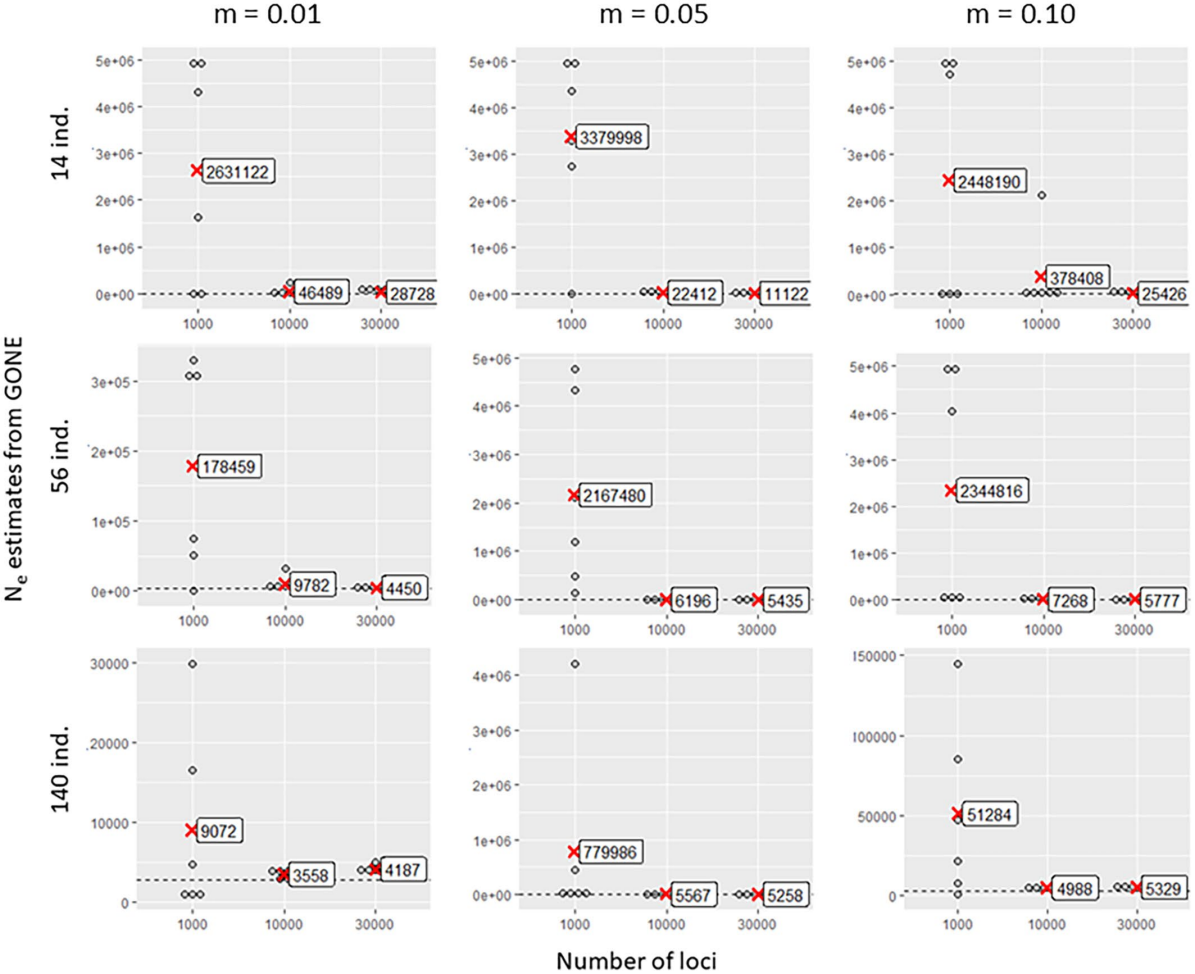
Estimated values of contemporary effective population size as a function of the number of loci involved in the analysis (1000, 10,000 or 30,000), obtained by the linkage disequilibrium method implemented in GONE software, which was applied to simulated data sets with gene flow between sub‐populations of *m* = 0.01, 0.05 or 0.10 and with 14, 56 or 140 individuals per sub‐population. Each point represents an estimate of the effective size of a sub‐population in one of the three simulation replicates. The mean effective size values within each locus number class are indicated by red cross markers. The dashed horizontal line in each graph represents the target effective size value (2772).

NeEstimator2 estimates seemed to be weakly influenced by the number of loci (Figure 7). However, higher counts (10,000 and 30,000) slightly reduced the variability and overestimation at high migration rates (0.05 and 0.10). This improvement was observed only for the largest sample size of 140 individuals (5% of the target *Ne*). GONE required more loci for reliable estimates, with 1000 loci yielding extreme values (Figure 8), in agreement with the recommendations of the authors (Santiago et al. 2020). Indeed, the use of too few loci does not provide sufficient information since loci need to be grouped according to their physical proximity on the genome (which is known in the context of our simulated data). A small number of loci per physical linkage class thus does not provide sufficient resolution for the estimates. Furthermore, consistent overestimation persisted, regardless of the locus count, sample size and gene flow.

#### 4.3.3 Influence of Gene Flow Between Sub‐Populations

**FIGURE 9:**
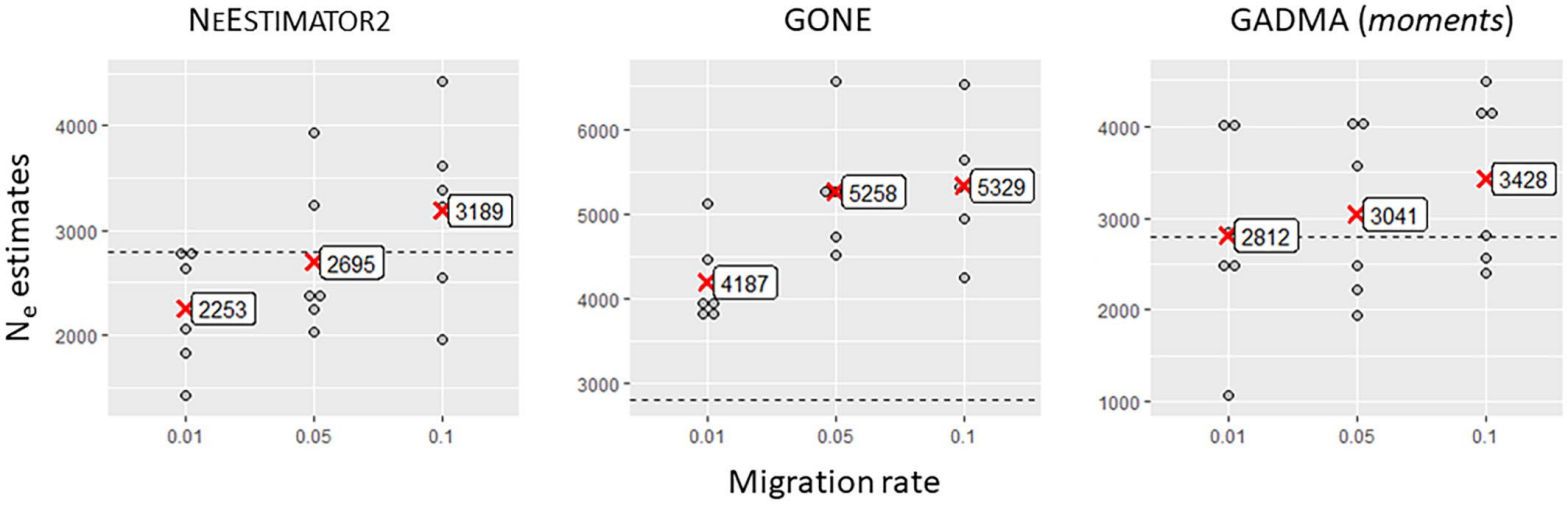
Estimated values of contemporary effective population size as a function of gene flow (0.01, 0.05 or 0.10), obtained by two linkage disequilibrium methods implemented in NeEstimator2 and GONE software and an allele frequency spectrum analysis method (moment‐based method) implemented in GADMA software; these methods were applied to simulated data sets with 30,000 loci and 140 individuals per sub‐population. Each point represents an estimate of the effective size of a sub‐population in one of the 3 simulation replicates. The mean effective size values within each gene flow class are indicated by red crosses. The dashed horizontal line in each graph represents the target effective size value (2772).

Under ‘ideal’ conditions of 140 individuals per sub‐population and 30,000 loci (Figure 9), higher gene flow (0.05, 0.10) systematically increased the mean *Ne* estimates. NeEstimator2 and GADMA performed well at low migration (0.01), but both slightly overestimated for higher gene flow (0.05 and 0.10). GONE consistently estimated the meta‐population *Ne* (approx. 5592) rather than the local *Ne*, which was consistent with expectations for symmetrical, constant gene flow.

The simulations demonstrate how data can be used to benchmark *Ne* estimation methods, emphasising the importance of sample size, locus count and gene flow considerations. Larger‐scale simulations and additional methods will further refine contemporary and historical *Ne* estimates (the latter are also accessible through GADMA and GONE). The integration of coalescent and forward‐time simulation tools enables the comparison of simulated and estimated historical *Ne* values, broadening applications in conservation and phylogeography.

## 5 Conclusion

### 5.1 *Ne* Estimation on the Basis of Linkage Disequilibrium

This work highlights significant advancements in the estimation of effective population sizes via high‐density genomic data sets. These data sets, however, face challenges such as pseudo‐replication caused by physical linkage or non‐independent LD values, which lead to biased *Ne* estimates and overestimated precision. Correction methods exist, but they strongly depend on factors such as sample size and marker availability.

LD‐based *Ne* estimation offers insights into temporal demographic trends. Methods have been developed for estimating *Ne* values at different temporal scales and for incorporating recombination data to refine analyses. However, these approaches require many genetic markers and are sensitive to sample size and population structure. While high‐density data sets are promising for understanding population dynamics, they must be cautiously applied to address methodological challenges. The recent advancements in this field, however, pave the way for improved application conservation and genetic resource management.

### 5.2 *Ne* Estimation on the Basis of Allele Frequency Spectrum Analysis

Inference methods based on the study of allele frequency spectra (SFS) effectively trace the evolutionary trajectories of populations but are less informative regarding recent events, especially in high‐abundance populations such as large pelagic species. Recombination between markers is often unaccounted for, complicating analyses of species that have faced recent anthropogenic pressures. Successful inference depends on data quantity and quality, including sufficient SNP markers and strategic temporal and/or geographic sampling to capture genetic diversity. In the case of large pelagic populations, where sampling can be challenging because of their wide distribution range, temporal and geographic sampling strategies may be necessary to capture genetic diversity and detect recent demographic events.

Furthermore, interpreting SFS data requires the development of realistic demographic scenarios and parameters informed by biological, phylogeographic and genetic knowledge. Comparing theoretical scenarios helps identify the best fit for empirical data, although the choice of inference algorithm and demographic model can also impact the results obtained. Recent developments in this field include genetic algorithms, which can help optimise scenario selection and can increase the duration and reliability of SFS‐based analyses. In conclusion, despite the challenges and precautions needed, SFS methods yield valuable insights into the evolution and conservation of large pelagic populations and could significantly contribute to the conservation and sustainable management of these populations.

### 5.3 Simulation Framework

The simulation framework, which is based on recently developed tools, provides important perspectives for the generation of high‐density demographic, individual‐based and genomic data for model testing and comparison. The use of NeEstimator2, GONE and GADMA to estimate *Ne* from 108 simulated data sets with varying numbers of loci, diploid samples and symmetrical migration rates, as expected, revealed that higher sample sizes (e.g., 50+ individuals) and locus densities (up to 30,000) improved the *Ne* estimates. However, these estimates remained variable across replicates and sub‐populations, even when using the highest values for both samples and loci. Importantly, however, the simulations also highlighted the risks of slightly overestimating *Ne* with higher levels of locus density and increased migration rates. Although this limited simulation exercise requires further improvement, it provides a pedagogical example of how currently developed simulation tools could help address questions related to *Ne* estimation in populations with different vital rates, demographic parameters, genome properties and effective and census sizes. Improvements to this simulation framework will include the ability to scale up to much larger populations sizes, for example, simulating biologically realistic populations with *Ne* of up to millions. In particular, this requires the ability to computationally handle both very large number of samples (1% of *Ne* of the order 10^6^ representing tens of thousands of individuals) and many recombination events within a given tree sequence, while avoiding time‐prohibitive simulation process and memory crash. In our framework, we covered several key capabilities offered by the software programs SLiM and *pyslim* (e.g., serial sampling and pedigree recording) which increases computation time. A less generic simulation can help to reduce simulation time, for example, by focusing on generating simulated genotypes only from present time and without keeping track of related individuals.

## Conflicts of Interest

The authors declare no conflicts of interest.

## Supporting information


Data S1.

Data S2.

Appendix S1.–S4.

## Data Availability

The simulation data presented in this article are publicly available in genepop format on Dryad (DOI: 10.5061/dryad.6wwpzgn9w). The Eidos, Python and R scripts used to simulate and process those genotype data with SLiM, pyslim and msprime are available from the online version of this article (Data S1 and Data S2) and on GitHub following the link: https://github.com/ChrystelleDelord/POPSIZE‐Project‐SLiM_Scripts.
