# Supplementary material for: Effective Population Size Estimation in Large Marine Populations: Considering Current Challenges and Opportunities When Simulating Large Data Sets With High‐Density Genomic Information
Source: Evol Appl. 2025 Jul 28;18(8):e70121. doi: 10.1111/eva.70121 (PMC12304085; doi:10.1111/eva.70121)
Supplement: Supplementary file 1 — Data S1. [file EVA-18-e70121-s002.html]

Post-processing of the demographic and genotypic data simulated via SLiM and pyslim/msprime.


# Post-processing of the demographic and genotypic data simulated via SLiM and pyslim/msprime.

#### Delord, C.

#### June 2023

## 1. Objectives:

This working document has been written as part of the POPSIZE project (IRD UMR Marbec, CRPMEM de La Réunion, FEAMP Measure 28 project).

It details the commands used in R software to post-process data from simulations generated using SLiM software and the *pyslim*/*msprime* Python libraries as part of the POPSIZE project. The scripts used to generate the simulated data are available at https://github.com/ChrystelleDelord/POPSIZE-Project-SLiM\_Scripts.) For further information or questions, please feel free to send an e-mail to chrys.delord@gmail.com.

The commands listed below perform the following actions:

- Import into R the global reference .vcf file, containing the genetic information of all individuals at all loci simulated and then “sampled” using SLiM software and the *pyslim*/*msprime* Python libraries.
- Import information from other simulation output files, in particular `SLiM_samples.tsv`, which contains the metadata of all individuals included in the global .vcf file.
- Perform subsampling amongst individuals and loci from the dataset saved at the end of the simulation. In this case, three datasets are generated, containing all individuals and, respectively, 30000 (i.e., all loci), 10000 and 1000 randomly sub-sampled loci. Then, from each of these 3 datasets, we generate 4 sub-datasets containing an increasing number of individuals previously randomly sub-sampled from the `SLiM_samples.tsv` file.
- Convert the format of the resulting 12 datasets in total. These datasets will be converted into genepop format (files with extension .gen) to be readable by *NeEstimator v2* software *Do et al. 2014*, and into PLINK format (files with extension .bed and .map) to be readable by *GONE* software *Santiago et al. 2020*. A file defining the population to which each individual belongs is also generated for each of the 4 sub-samples of individuals to be read, in parallel with the global .vcf file, by the *GADMA* software *Noskova et al. 2020*.
- Preliminary visualization of the information related to the various sub-samples of individuals, and in particular the number of related pairs that can be found (in connection with *close-kin mark recapture* applications).

## 2. Post-processing and conversion of genotypic data in a perspective of evaluating various effective size estimation softwares:

### Loading libraries and data:

```
library(dartR)
library(CKMRpop)
library(psych)
library(tidyverse)

str1 = "5000"
str2 = "m005"
migrate = 0.05
```

```
# ---------------------------------------------------------------
# DATA LOADING:
# ---------------------------------------------------------------
# -- Upload genotypic data from the global .vcf file (output from SLiM and pyslim/msprime simulations):
start_time = Sys.time()
dat <- gl.read.vcf(paste0("POPSIZE_pyslim_output_Cohort", str1, "_", str2, ".vcf"))
end_time = Sys.time()
# end_time - start_time
```

```
# -- Metadata from the individuals sampled during the simulation process:
sample_file <- file.path(getwd(), "SLiM_samples.tsv")
samples <- vroom::vroom(file = sample_file, delim = "\t", col_types = "ccccccc") %>% 
  mutate(samp_years_list_post = str_split(syears_post, "  *"), samp_years_list_post = map(.x = samp_years_list_post,
  .f = function(x) as.integer(x)), sampling_pop = str_split(pop_post, "  *"), sampling_pop = map(.x = sampling_pop, 
  .f = function(x) as.integer(x))) %>% select(-syears_pre, -syears_post, -syears_dur, -pop_pre, -pop_post, -pop_dur) %>% 
  extract(ID, into = c("sex", "born_year", "born_pop"), regex = "^([MF])([0-9]+)_([0-9]+)", remove = FALSE, convert = TRUE)
samples <- samples %>% unnest(samp_years_list_post, sampling_pop)
```

```
## Warning: unnest() has a new interface. See ?unnest for details.
## Try `df %>% unnest(c(samp_years_list_post, sampling_pop))`, with `mutate()` if needed
```

```
samples <- samples %>% mutate(samples, age_at_sampling = samp_years_list_post - born_year)

print("Our full genotypic dataset in genlight format:")
```

```
## [1] "Our full genotypic dataset in genlight format:"
```

```
show(dat)
```

```
##  /// GENLIGHT OBJECT /////////
## 
##  // 14,856 genotypes,  30,000 binary SNPs, size: 134.4 Mb
##  0 (0 %) missing data
## 
##  // Basic content
##    @gen: list of 14856 SNPbin
##    @ploidy: ploidy of each individual  (range: 2-2)
## 
##  // Optional content
##    @ind.names:  14856 individual labels
##    @loc.names:  30000 locus labels
##    @loc.all:  30000 alleles
##    @chromosome: factor storing chromosomes of the SNPs
##    @position: integer storing positions of the SNPs
##    @pop: population of each individual (group size range: 14856-14856)
##    @other: a list containing: loc.metrics  loc.metrics.flags  verbose  history  ind.metrics
```

```
print("Number of individuals collected in SLiM for each sampled time step, i.e. reproductive cycle:")
```

```
## [1] "Number of individuals collected in SLiM for each sampled time step, i.e. reproductive cycle:"
```

```
table(samples$samp_years_list_post)
```

```
## 
##   90   91   92   93   94   95   96   97   98   99  100 
## 1534 1591 1546 1500 1480 1580 1560 1506 1540 1520 1591
```

```
print("Number of individuals collected in SLiM for each sampled time step, i.e. reproductive cycle and per sub-population:")
```

```
## [1] "Number of individuals collected in SLiM for each sampled time step, i.e. reproductive cycle and per sub-population:"
```

```
table(samples$sampling_pop, samples$samp_years_list_post)
```

```
##    
##      90  91  92  93  94  95  96  97  98  99 100
##   1 768 767 780 754 711 802 761 775 774 753 806
##   2 766 824 766 746 769 778 799 731 766 767 785
```

```
print("Number of individuals collected in SLiM for each sampled time step, i.e. reproductive cycle and per age class:")
```

```
## [1] "Number of individuals collected in SLiM for each sampled time step, i.e. reproductive cycle and per age class:"
```

```
table(samples$age_at_sampling, samples$samp_years_list_post)
```

```
##     
##       90  91  92  93  94  95  96  97  98  99 100
##   1  548 546 560 509 473 580 547 525 530 521 579
##   2  333 320 322 324 340 328 326 312 340 330 333
##   3  218 230 224 220 211 224 236 211 220 227 235
##   4  160 171 147 166 159 156 155 136 165 162 161
##   5   83 118 112  97 122 106 116 120  88  98 103
##   6   81  87  74  88  72  73  64  84  74  55  56
##   7   48  53  45  44  48  54  55  54  58  51  51
##   8   28  42  40  21  29  31  29  32  33  36  34
##   9   22  16  17  19  12  11  17  17  17  24  21
##   10   9   7   3   7   9  10  11   8   9   7   9
##   11   3   1   1   2   2   4   3   6   3   3   6
##   12   1   0   1   2   2   2   1   0   1   3   3
##   13   0   0   0   1   1   0   0   1   2   3   0
##   14   0   0   0   0   0   1   0   0   0   0   0
```

### Successive (hierarchical) sub-sampling of loci:

Our `dat` object contains 14856 individuals and 30000 loci from SLiM and *pyslim*/*msprime*. From `dat`, we create `dat_L10000` which contains 14856 individuals and 10000 loci. From `dat_L10000`, we create `dat_L1000`, which contains 14856 individuals and 1000 loci.

This procedure can be used, for example, to test the influence of the number of loci on the quality of effective size estimation, using the same individuals.

```
# ---------------------------------------------------------------
# SUB-SAMPLING OF LOCI:
# ---------------------------------------------------------------
dat_L10000 <- gl.keep.loc(dat, loc.list = sample(dat$loc.names, 10000, replace = FALSE, prob = NULL), first = NULL, last = NULL, verbose = NULL)
dat_L1000 <- gl.keep.loc(dat_L10000, loc.list = sample(dat_L10000$loc.names, 1000, replace = FALSE, prob = NULL), first = NULL, last = NULL, verbose = NULL)
```

### Sub-sampling of individuals:

Our reference .vcf file contains 14856 individuals sampled between time steps 90 and 100, in each of the age classes 1 to 15 and in each of the sub-populations. In order to test the influence of realistic sampling strategies on the quality of effective size estimates, we will need to subsample to retain only genotypic information from individuals reflecting a real field sampling campaign. For example, it’s unlikely that we’ll be able to sample individuals over 11 consecutive time steps in the real world. We can, however, focus on a given time step to reflect a localized sampling over time.

As an example, we will therefore carry out several sub-samples of increasing size in number of individuals, but focused on the last time step simulated in SLiM: time step 100, which corresponds to the most recent (=present time). On the other hand, we will sub-sample the same number of individuals in each of the two sub-populations.

In addition, we want the same individuals to be sub-sampled in each dataset at 30000, 10000 and 1000 loci. This will enable us to compare the influence of the number of loci on the quality of effective size estimates, without incorporating “noise” linked to the use of different individuals. Conversely, all individuals will be genotyped on the same 30000, 10000 or 1000 loci.

Using the `samples` object from the `SLiM_samples.tsv` file, we collect a number of individuals varying between 0.5%, 2.0% and 5.0% of the local effective size simulated in the SLiM software, in each of the sub-populations. Here, these sub-sample sizes correspond to 14, 55 and 139 individuals per sub-population, respectively. A “typical” sub-sample of 50 individuals is also collected, again in each sub-population.

```
# ---------------------------------------------------------------
# SUB-SAMPLING OF INDIVIDUALS:
# ---------------------------------------------------------------

# Loading of demographic effective size values calculated during the simulation phase using SLiM software.
if(file.exists("SLiM_demo_table.tsv")) {
  SLiM_demo <- read.table("SLiM_demo_table.tsv", sep ='\t', header = T)
  Ne_demo = (harmonic.mean(SLiM_demo$ne_demo))/2
} else {
  Ne_demo = round(0.559*strtoi(str1))
}

# Four sub-samples are taken at the present time (time-step 100), with a variable number of individuals per sub-population:
Ssize_005percent <- round(0.005*Ne_demo) # A sample size corresponding to 0.5% of the local effective size: 14 individuals.
Ssize_02percent <- round(0.02*Ne_demo) # A sample size corresponding to 2.0% of the local effective size: 55 individuals.
Ssize_05percent <- round(0.05*Ne_demo) # A sample size corresponding to  5.0% of the local effective size: 139 individuals.
Ssize_typical <- 50 # A "typical" sample size of 50 individuals.

# Sub-sampling of individuals (using their unique ID) with a sample size per sub-population corresponding to 0.5% of local effective size.
subsamp_ID_005 <- samples %>% filter(samp_years_list_post %in% c(100), sampling_pop %in% c(1,2)) %>% 
  group_by(samp_years_list_post, sampling_pop) %>% sample_n(if(n() < Ssize_005percent) n() else Ssize_005percent)
table(subsamp_ID_005$age_at_sampling, subsamp_ID_005$samp_years_list_post) # Number of samples per age class.
table(subsamp_ID_005$sampling_pop, subsamp_ID_005$samp_years_list_post) # Number of samples per sub-population.
subsamp_ID_005 %>% group_by(ID) %>% filter(n() > 1) %>% print(n = 20)

# Sub-sampling of individuals (using their unique ID) with a sample size per sub-population corresponding to 2.0% of local effective size.
subsamp_ID_02 <- samples %>% filter(samp_years_list_post %in% c(100), sampling_pop %in% c(1,2)) %>% 
  group_by(samp_years_list_post, sampling_pop) %>% sample_n(if(n() < Ssize_02percent) n() else Ssize_02percent)
#table(subsamp_ID_02$age_at_sampling, subsamp_ID_02$samp_years_list_post)
subsamp_ID_02 %>% group_by(ID) %>% filter(n() > 1) %>% print(n = 20)

# Sub-sampling of individuals (using their unique ID) with a sample size per sub-population corresponding to 5.0% of local effective size.
subsamp_ID_05 <- samples %>% filter(samp_years_list_post %in% c(100), sampling_pop %in% c(1,2)) %>% 
  group_by(samp_years_list_post, sampling_pop) %>% sample_n(if(n() < Ssize_05percent) n() else Ssize_05percent)
#table(subsamp_ID_05$age_at_sampling, subsamp_ID_05$samp_years_list_post)
subsamp_ID_05 %>% group_by(ID) %>% filter(n() > 1) %>% print(n = 20)

# Sub-sampling of individuals (using their unique ID) with a sample size of 50.
subsamp_ID_typical <- samples %>% filter(samp_years_list_post %in% c(100), sampling_pop %in% c(1,2)) %>% 
  group_by(samp_years_list_post, sampling_pop) %>% sample_n(if(n() < Ssize_typical) n() else Ssize_typical)
#table(subsamp_ID_typical$age_at_sampling, subsamp_ID_typical$samp_years_list_post)
subsamp_ID_typical %>% group_by(ID) %>% filter(n() > 1) %>% print(n = 20)
```

### Extracting genotypes and information from sub-sampled individuals:

We have at our disposal, on the one hand, our genotypic datasets of 30000, 10000 or 1000 loci for all 14856 individuals, and on the other hand, lists (e.g., `subsamp_ID_005`) containing the identifiers of the individuals we wish to extract from each of these datasets. The command lines below perform several manipulations to ensure correct extraction of these samples and their information, including the population from which they were sampled.

```
# ---------------------------------------------------------------
# EXPORT OF A FILE (strata file) CONTAINING THE POPULATION FROM WHICH EACH INDIVIDUAL HAS BEEN SUB-SAMPLED
# (from the unique identifier of each individual and the information contained in the 'samples' object)
# ---------------------------------------------------------------

stratat_005 <- as.data.frame(subsamp_ID_005 %>% ungroup() %>% select(ID, sampling_pop))
names(stratat_005) <- c("sample", "pop")
write.table(stratat_005, paste0("pop_file_", str1, "_", str2,".txt") ,sep="\t", row.names=FALSE, quote=FALSE)

stratat_02 <- as.data.frame(subsamp_ID_02 %>% ungroup() %>% select(ID, sampling_pop))
names(stratat_02) <- c("sample", "pop")
write.table(stratat_02, paste0("pop_file_", str1, "_", str2, "_s02.txt") ,sep="\t", row.names=FALSE, quote=FALSE)

stratat_05 <- as.data.frame(subsamp_ID_05 %>% ungroup() %>% select(ID, sampling_pop))
names(stratat_05) <- c("sample", "pop")
write.table(stratat_05, paste0("pop_file_", str1, "_", str2, "_s05.txt") ,sep="\t", row.names=FALSE, quote=FALSE)

stratat_typical <- as.data.frame(subsamp_ID_typical %>% ungroup() %>% select(ID, sampling_pop))
names(stratat_typical) <- c("sample", "pop")
write.table(stratat_typical, paste0("pop_file_", str1, "_", str2, "_stypical.txt") ,sep="\t", row.names=FALSE, quote=FALSE)
```

Groups of sub-sampled individuals are now extracted from each of the datasets `dat` (30000 loci), `dat_L10000` (10000 loci) and `dat_L1000` (1000 loci). For each of these, 4 new datasets are generated.

Then, for each of the 12 final datasets, the original population information is reassigned to each of the individuals it contains, in preparation for future data format conversions.

```
# ---------------------------------------------------------------
# EXTRACTION FROM THE DATASET WITH 30000 LOCI:
# ---------------------------------------------------------------

ind.list_005 <- dat$ind.names[dat$ind.names %in% subsamp_ID_005$ID] # Listing the IDs of sub-sampled individuals.
dat_s005 <- gl.keep.ind(dat, ind.list=ind.list_005, recalc = FALSE, mono.rm = FALSE, verbose = NULL)
dat_s005@other$ind.metrics$pop <- pop(dat_s005)
dat_s005$other$ind.metrics$pop <- ifelse(stratat_005$sample %in% dat_s005$other$ind.metrics$id, stratat_005$pop, dat_s005$other$ind.metrics$pop)
# Updating the sub-population information:
dat_s005 <- gl.reassign.pop(dat_s005, as.pop='pop',verbose=3)
popNames(dat_s005)

# ... and so on for each sample size.
ind.list_02 <- dat$ind.names[dat$ind.names %in% subsamp_ID_02$ID]
dat_s02 <- gl.keep.ind(dat, ind.list=ind.list_02, recalc = FALSE, mono.rm = FALSE, verbose = NULL)
dat_s02@other$ind.metrics$pop <- pop(dat_s02)
dat_s02$other$ind.metrics$pop <- ifelse(stratat_02$sample %in% dat_s02$other$ind.metrics$id, stratat_02$pop, dat_s02$other$ind.metrics$pop)
dat_s02 <- gl.reassign.pop(dat_s02, as.pop='pop',verbose=3)
popNames(dat_s02)

ind.list_05 <- dat$ind.names[dat$ind.names %in% subsamp_ID_05$ID]
dat_s05 <- gl.keep.ind(dat, ind.list=ind.list_05, recalc = FALSE, mono.rm = FALSE, verbose = NULL)
dat_s05@other$ind.metrics$pop <- pop(dat_s05)
dat_s05$other$ind.metrics$pop <- ifelse(stratat_05$sample %in% dat_s05$other$ind.metrics$id, stratat_05$pop, dat_s05$other$ind.metrics$pop)
dat_s05 <- gl.reassign.pop(dat_s05, as.pop='pop',verbose=3)
popNames(dat_s05)

ind.list_typ <- dat$ind.names[dat$ind.names %in% subsamp_ID_typical$ID]
dat_styp <- gl.keep.ind(dat, ind.list=ind.list_typ, recalc = FALSE, mono.rm = FALSE, verbose = NULL)
dat_styp@other$ind.metrics$pop <- pop(dat_styp)
dat_styp$other$ind.metrics$pop <- ifelse(stratat_typical$sample %in% dat_styp$other$ind.metrics$id, stratat_typical$pop, dat_styp$other$ind.metrics$pop)
dat_styp <- gl.reassign.pop(dat_styp, as.pop='pop',verbose=3)
popNames(dat_styp)

# ---------------------------------------------------------------
# EXTRACTION FROM THE DATASET WITH 10000 LOCI:
# ---------------------------------------------------------------

ind.list_005 <- dat_L10000$ind.names[dat_L10000$ind.names %in% subsamp_ID_005$ID]
dat_L10000_s005 <- gl.keep.ind(dat_L10000, ind.list=ind.list_005, recalc = FALSE, mono.rm = FALSE, verbose = NULL)
dat_L10000_s005@other$ind.metrics$pop <- pop(dat_L10000_s005)
dat_L10000_s005$other$ind.metrics$pop <- ifelse(stratat_005$sample %in% dat_L10000_s005$other$ind.metrics$id, stratat_005$pop, dat_L10000_s005$other$ind.metrics$pop)
dat_L10000_s005 <- gl.reassign.pop(dat_L10000_s005, as.pop='pop',verbose=3)
popNames(dat_L10000_s005)

ind.list_02 <- dat_L10000$ind.names[dat_L10000$ind.names %in% subsamp_ID_02$ID]
dat_L10000_s02 <- gl.keep.ind(dat_L10000, ind.list=ind.list_02, recalc = FALSE, mono.rm = FALSE, verbose = NULL)
dat_L10000_s02@other$ind.metrics$pop <- pop(dat_L10000_s02)
dat_L10000_s02$other$ind.metrics$pop <- ifelse(stratat_02$sample %in% dat_L10000_s02$other$ind.metrics$id, stratat_02$pop, dat_L10000_s02$other$ind.metrics$pop)
dat_L10000_s02 <- gl.reassign.pop(dat_L10000_s02, as.pop='pop',verbose=3)
popNames(dat_L10000_s02)

ind.list_05 <- dat_L10000$ind.names[dat_L10000$ind.names %in% subsamp_ID_05$ID]
dat_L10000_s05 <- gl.keep.ind(dat_L10000, ind.list=ind.list_05, recalc = FALSE, mono.rm = FALSE, verbose = NULL)
dat_L10000_s05@other$ind.metrics$pop <- pop(dat_L10000_s05)
dat_L10000_s05$other$ind.metrics$pop <- ifelse(stratat_05$sample %in% dat_L10000_s05$other$ind.metrics$id, stratat_05$pop, dat_L10000_s05$other$ind.metrics$pop)
dat_L10000_s05 <- gl.reassign.pop(dat_L10000_s05, as.pop='pop',verbose=3)
popNames(dat_L10000_s05)

ind.list_typ <- dat_L10000$ind.names[dat_L10000$ind.names %in% subsamp_ID_typical$ID] # lists individuals to keep.
dat_L10000_styp <- gl.keep.ind(dat_L10000, ind.list=ind.list_typ, recalc = FALSE, mono.rm = FALSE, verbose = NULL)
dat_L10000_styp@other$ind.metrics$pop <- pop(dat_L10000_styp)
dat_L10000_styp$other$ind.metrics$pop <- ifelse(stratat_typical$sample %in% dat_L10000_styp$other$ind.metrics$id, stratat_typical$pop, dat_L10000_styp$other$ind.metrics$pop)
dat_L10000_styp <- gl.reassign.pop(dat_L10000_styp, as.pop='pop',verbose=3)
popNames(dat_L10000_styp)

# ---------------------------------------------------------------
# EXTRACTION FROM THE DATASET WITH 30000 LOCI:
# ---------------------------------------------------------------

ind.list_005 <- dat_L1000$ind.names[dat_L1000$ind.names %in% subsamp_ID_005$ID] 
dat_L1000_s005 <- gl.keep.ind(dat_L1000, ind.list=ind.list_005, recalc = FALSE, mono.rm = FALSE, verbose = NULL)
dat_L1000_s005@other$ind.metrics$pop <- pop(dat_L1000_s005)
dat_L1000_s005$other$ind.metrics$pop <- ifelse(stratat_005$sample %in% dat_L1000_s005$other$ind.metrics$id, stratat_005$pop, dat_L1000_s005$other$ind.metrics$pop)
dat_L1000_s005 <- gl.reassign.pop(dat_L1000_s005, as.pop='pop',verbose=3)
popNames(dat_L1000_s005)

ind.list_02 <- dat_L1000$ind.names[dat_L1000$ind.names %in% subsamp_ID_02$ID]
dat_L1000_s02 <- gl.keep.ind(dat_L1000, ind.list=ind.list_02, recalc = FALSE, mono.rm = FALSE, verbose = NULL)
dat_L1000_s02@other$ind.metrics$pop <- pop(dat_L1000_s02)
dat_L1000_s02$other$ind.metrics$pop <- ifelse(stratat_02$sample %in% dat_L1000_s02$other$ind.metrics$id, stratat_02$pop, dat_L1000_s02$other$ind.metrics$pop)
dat_L1000_s02 <- gl.reassign.pop(dat_L1000_s02, as.pop='pop',verbose=3)
popNames(dat_L1000_s02)

ind.list_05 <- dat_L1000$ind.names[dat_L1000$ind.names %in% subsamp_ID_05$ID]
dat_L1000_s05 <- gl.keep.ind(dat_L1000, ind.list=ind.list_05, recalc = FALSE, mono.rm = FALSE, verbose = NULL)
dat_L1000_s05@other$ind.metrics$pop <- pop(dat_L1000_s05)
dat_L1000_s05$other$ind.metrics$pop <- ifelse(stratat_05$sample %in% dat_L1000_s05$other$ind.metrics$id, stratat_05$pop, dat_L1000_s05$other$ind.metrics$pop)
dat_L1000_s05 <- gl.reassign.pop(dat_L1000_s05, as.pop='pop',verbose=3)
popNames(dat_L1000_s05)

ind.list_typ <- dat_L1000$ind.names[dat_L1000$ind.names %in% subsamp_ID_typical$ID]
dat_L1000_styp <- gl.keep.ind(dat_L1000, ind.list=ind.list_typ, recalc = FALSE, mono.rm = FALSE, verbose = NULL)
dat_L1000_styp@other$ind.metrics$pop <- pop(dat_L1000_styp)
dat_L1000_styp$other$ind.metrics$pop <- ifelse(stratat_typical$sample %in% dat_L1000_styp$other$ind.metrics$id, stratat_typical$pop, dat_L1000_styp$other$ind.metrics$pop)
dat_L1000_styp <- gl.reassign.pop(dat_L1000_styp, as.pop='pop',verbose=3)
popNames(dat_L1000_styp)
```

### Converting and exporting genotypes for all sub-sampled individuals:

Genotype data for each of the 12 datasets are exported in different formats:

```
# ---------------------------------------------------------------
# CONVERSION IN GENEPOP FORMAT (.GEN):
# ---------------------------------------------------------------

gl2genepop(dat_s005, outfile = paste0("gen_output_", str1, "_", str2, "_L30000_s005.gen"), outpath = getwd(), verbose = NULL)
gl2genepop(dat_s02, outfile = paste0("gen_output_", str1, "_", str2, "_L30000_s02.gen"), outpath = getwd(), verbose = NULL)
gl2genepop(dat_s05, outfile = paste0("gen_output_", str1, "_", str2, "_L30000_s05.gen"), outpath = getwd(), verbose = NULL)
gl2genepop(dat_styp, outfile = paste0("gen_output_", str1, "_", str2, "_L30000_styp.gen"), outpath = getwd(), verbose = NULL)
gl2genepop(dat_L10000_s005, outfile = paste0("gen_output_", str1, "_", str2, "_L10000_s005.gen"), outpath = getwd(), verbose = NULL)
gl2genepop(dat_L10000_s02, outfile = paste0("gen_output_", str1, "_", str2, "_L10000_s02.gen"), outpath = getwd(), verbose = NULL)
gl2genepop(dat_L10000_s05, outfile = paste0("gen_output_", str1, "_", str2, "_L10000_s05.gen"), outpath = getwd(), verbose = NULL)
gl2genepop(dat_L10000_styp, outfile = paste0("gen_output_", str1, "_", str2, "_L10000_styp.gen"), outpath = getwd(), verbose = NULL)
gl2genepop(dat_L1000_s005, outfile = paste0("gen_output_", str1, "_", str2, "_L1000_s005.gen"), outpath = getwd(), verbose = NULL)
gl2genepop(dat_L1000_s02, outfile = paste0("gen_output_", str1, "_", str2, "_L1000_s02.gen"), outpath = getwd(), verbose = NULL)
gl2genepop(dat_L1000_s05, outfile = paste0("gen_output_", str1, "_", str2, "_L1000_s05.gen"), outpath = getwd(), verbose = NULL)
gl2genepop(dat_L1000_styp, outfile = paste0("gen_output_", str1, "_", str2, "_L1000_styp.gen"), outpath = getwd(), verbose = NULL)

# ---------------------------------------------------------------
# CONVERSION IN PLINK FORMAT (.PED et .MAP):
# ---------------------------------------------------------------

# Here, we are going to reintegrate the chromosome structure information into our files. This is the subdivision into 5 recombinant chromosomes that had been programmed in the SLiM software.
gen_list <- c(dat_L1000_s005, dat_L1000_s02, dat_L1000_s05, dat_L1000_styp, dat_L10000_s005, dat_L10000_s02, dat_L10000_s05, dat_L10000_styp, dat_s005, dat_s02, dat_s05, dat_styp)
names(gen_list) <- c("dat_L1000_s005", "dat_L1000_s02", "dat_L1000_s05", "dat_L1000_styp", "dat_L10000_s005", "dat_L10000_s02", "dat_L10000_s05", "dat_L10000_styp", "dat_s005", "dat_s02", "dat_s05", "dat_styp")

for (dat0 in names(gen_list)) {
  dat <- gen_list[[dat0]]
  dat$other$loc.metrics$position <- dat$position
  dat$other$loc.metrics$chromosome <- dat$chromosome
  dat$other$loc.metrics$chromosome <- ifelse(dat$other$loc.metrics$position %in% c(1:400000000), 1, dat$other$loc.metrics$chromosome)
  dat$other$loc.metrics$chromosome <- ifelse(dat$other$loc.metrics$position %in% c(400000001:800000000), 2, dat$other$loc.metrics$chromosome)
  dat$other$loc.metrics$chromosome <- ifelse(dat$other$loc.metrics$position %in% c(800000001:1200000000), 3, dat$other$loc.metrics$chromosome)
  dat$other$loc.metrics$chromosome <- ifelse(dat$other$loc.metrics$position %in% c(1200000001:1600000000), 4, dat$other$loc.metrics$chromosome)
  dat$other$loc.metrics$chromosome <- ifelse(dat$other$loc.metrics$position %in% c(1600000001:2000000000), 5, dat$other$loc.metrics$chromosome)
  dat$chromosome <- as.factor(dat$other$loc.metrics$chromosome)
  assign(paste0(dat0, "_p1"), gl.keep.pop(dat, "1"))
  assign(paste0(dat0, "_p2"), gl.keep.pop(dat, "2"))
}

# We can convert and export files for each sub-sample of individuals and each sub-population (p1 and p2):
gl2plink(dat_s005_p1, plink_path = "../PLINK_linux", bed_file=FALSE, outfile = paste0("ped_output_", str1, "_", str2, "_L30000_s005_p1"), outpath = getwd(), chr_format = "character", pos_cM = "0", ID_dad = "0", ID_mom = "0", sex_code = "unknown", phen_value = "-9", verbose = NULL)
gl2plink(dat_s02_p1, plink_path = "../PLINK_linux", bed_file=FALSE, outfile = paste0("ped_output_", str1, "_", str2, "_L30000_s02_p1"), outpath = getwd(), chr_format = "character", pos_cM = "0", ID_dad = "0", ID_mom = "0", sex_code = "unknown", phen_value = "-9", verbose = NULL)
gl2plink(dat_s05_p1, plink_path = "../PLINK_linux", bed_file=FALSE, outfile = paste0("ped_output_", str1, "_", str2, "_L30000_s05_p1"), outpath = getwd(), chr_format = "character", pos_cM = "0", ID_dad = "0", ID_mom = "0", sex_code = "unknown", phen_value = "-9", verbose = NULL)
gl2plink(dat_styp_p1, plink_path = "../PLINK_linux", bed_file=FALSE, outfile = paste0("ped_output_", str1, "_", str2, "_L30000_styp_p1"), outpath = getwd(), chr_format = "character", pos_cM = "0", ID_dad = "0", ID_mom = "0", sex_code = "unknown", phen_value = "-9", verbose = NULL)
gl2plink(dat_L10000_s005_p1, plink_path = "../PLINK_linux", bed_file=FALSE, outfile = paste0("ped_output_", str1, "_", str2, "_L10000_s005_p1"), outpath = getwd(), chr_format = "character", pos_cM = "0", ID_dad = "0", ID_mom = "0", sex_code = "unknown", phen_value = "-9", verbose = NULL)
gl2plink(dat_L10000_s02_p1, plink_path = "../PLINK_linux", bed_file=FALSE, outfile = paste0("ped_output_", str1, "_", str2, "_L10000_s02_p1"), outpath = getwd(), chr_format = "character", pos_cM = "0", ID_dad = "0", ID_mom = "0", sex_code = "unknown", phen_value = "-9", verbose = NULL)
gl2plink(dat_L10000_s05_p1, plink_path = "../PLINK_linux", bed_file=FALSE, outfile = paste0("ped_output_", str1, "_", str2, "_L10000_s05_p1"), outpath = getwd(), chr_format = "character", pos_cM = "0", ID_dad = "0", ID_mom = "0", sex_code = "unknown", phen_value = "-9", verbose = NULL)
gl2plink(dat_L10000_styp_p1, plink_path = "../PLINK_linux", bed_file=FALSE, outfile = paste0("ped_output_", str1, "_", str2, "_L10000_styp_p1"), outpath = getwd(), chr_format = "character", pos_cM = "0", ID_dad = "0", ID_mom = "0", sex_code = "unknown", phen_value = "-9", verbose = NULL)
gl2plink(dat_L1000_s005_p1, plink_path = "../PLINK_linux", bed_file=FALSE, outfile = paste0("ped_output_", str1, "_", str2, "_L1000_s005_p1"), outpath = getwd(), chr_format = "character", pos_cM = "0", ID_dad = "0", ID_mom = "0", sex_code = "unknown", phen_value = "-9", verbose = NULL)
gl2plink(dat_L1000_s02_p1, plink_path = "../PLINK_linux", bed_file=FALSE, outfile = paste0("ped_output_", str1, "_", str2, "_L1000_s02_p1"), outpath = getwd(), chr_format = "character", pos_cM = "0", ID_dad = "0", ID_mom = "0", sex_code = "unknown", phen_value = "-9", verbose = NULL)
gl2plink(dat_L1000_s05_p1, plink_path = "../PLINK_linux", bed_file=FALSE, outfile = paste0("ped_output_", str1, "_", str2, "_L1000_s05_p1"), outpath = getwd(), chr_format = "character", pos_cM = "0", ID_dad = "0", ID_mom = "0", sex_code = "unknown", phen_value = "-9", verbose = NULL)
gl2plink(dat_L1000_styp_p1, plink_path = "../PLINK_linux", bed_file=FALSE, outfile = paste0("ped_output_", str1, "_", str2, "_L1000_styp_p1"), outpath = getwd(), chr_format = "character", pos_cM = "0", ID_dad = "0", ID_mom = "0", sex_code = "unknown", phen_value = "-9", verbose = NULL)

gl2plink(dat_s005_p2, plink_path = "../PLINK_linux", bed_file=FALSE, outfile = paste0("ped_output_", str1, "_", str2, "_L30000_s005_p2"), outpath = getwd(), chr_format = "character", pos_cM = "0", ID_dad = "0", ID_mom = "0", sex_code = "unknown", phen_value = "-9", verbose = NULL)
gl2plink(dat_s02_p2, plink_path = "../PLINK_linux", bed_file=FALSE, outfile = paste0("ped_output_", str1, "_", str2, "_L30000_s02_p2"), outpath = getwd(), chr_format = "character", pos_cM = "0", ID_dad = "0", ID_mom = "0", sex_code = "unknown", phen_value = "-9", verbose = NULL)
gl2plink(dat_s05_p2, plink_path = "../PLINK_linux", bed_file=FALSE, outfile = paste0("ped_output_", str1, "_", str2, "_L30000_s05_p2"), outpath = getwd(), chr_format = "character", pos_cM = "0", ID_dad = "0", ID_mom = "0", sex_code = "unknown", phen_value = "-9", verbose = NULL)
gl2plink(dat_styp_p2, plink_path = "../PLINK_linux", bed_file=FALSE, outfile = paste0("ped_output_", str1, "_", str2, "_L30000_styp_p2"), outpath = getwd(), chr_format = "character", pos_cM = "0", ID_dad = "0", ID_mom = "0", sex_code = "unknown", phen_value = "-9", verbose = NULL)
gl2plink(dat_L10000_s005_p2, plink_path = "../PLINK_linux", bed_file=FALSE, outfile = paste0("ped_output_", str1, "_", str2, "_L10000_s005_p2"), outpath = getwd(), chr_format = "character", pos_cM = "0", ID_dad = "0", ID_mom = "0", sex_code = "unknown", phen_value = "-9", verbose = NULL)
gl2plink(dat_L10000_s02_p2, plink_path = "../PLINK_linux", bed_file=FALSE, outfile = paste0("ped_output_", str1, "_", str2, "_L10000_s02_p2"), outpath = getwd(), chr_format = "character", pos_cM = "0", ID_dad = "0", ID_mom = "0", sex_code = "unknown", phen_value = "-9", verbose = NULL)
gl2plink(dat_L10000_s05_p2, plink_path = "../PLINK_linux", bed_file=FALSE, outfile = paste0("ped_output_", str1, "_", str2, "_L10000_s05_p2"), outpath = getwd(), chr_format = "character", pos_cM = "0", ID_dad = "0", ID_mom = "0", sex_code = "unknown", phen_value = "-9", verbose = NULL)
gl2plink(dat_L10000_styp_p2, plink_path = "../PLINK_linux", bed_file=FALSE, outfile = paste0("ped_output_", str1, "_", str2, "_L10000_styp_p2"), outpath = getwd(), chr_format = "character", pos_cM = "0", ID_dad = "0", ID_mom = "0", sex_code = "unknown", phen_value = "-9", verbose = NULL)
gl2plink(dat_L1000_s005_p2, plink_path = "../PLINK_linux", bed_file=FALSE, outfile = paste0("ped_output_", str1, "_", str2, "_L1000_s005_p2"), outpath = getwd(), chr_format = "character", pos_cM = "0", ID_dad = "0", ID_mom = "0", sex_code = "unknown", phen_value = "-9", verbose = NULL)
gl2plink(dat_L1000_s02_p2, plink_path = "../PLINK_linux", bed_file=FALSE, outfile = paste0("ped_output_", str1, "_", str2, "_L1000_s02_p2"), outpath = getwd(), chr_format = "character", pos_cM = "0", ID_dad = "0", ID_mom = "0", sex_code = "unknown", phen_value = "-9", verbose = NULL)
gl2plink(dat_L1000_s05_p2, plink_path = "../PLINK_linux", bed_file=FALSE, outfile = paste0("ped_output_", str1, "_", str2, "_L1000_s05_p2"), outpath = getwd(), chr_format = "character", pos_cM = "0", ID_dad = "0", ID_mom = "0", sex_code = "unknown", phen_value = "-9", verbose = NULL)
gl2plink(dat_L1000_styp_p2, plink_path = "../PLINK_linux", bed_file=FALSE, outfile = paste0("ped_output_", str1, "_", str2, "_L1000_styp_p2"), outpath = getwd(), chr_format = "character", pos_cM = "0", ID_dad = "0", ID_mom = "0", sex_code = "unknown", phen_value = "-9", verbose = NULL)
```

These exported files can now be used as inputs for the software packages NeEstimator, GONE and GADMA.

End of R Markdown document.

## R Configuration:

```
options(width = 100)
devtools::session_info()
```

```
## - Session info -----------------------------------------------------------------------------------
##  setting  value
##  version  R version 4.0.4 (2021-02-15)
##  os       Windows 10 x64 (build 19045)
##  system   x86_64, mingw32
##  ui       RTerm
##  language (EN)
##  collate  French_France.1252
##  ctype    French_France.1252
##  tz       Europe/Paris
##  date     2024-03-30
##  pandoc   2.7.2 @ C:/Program Files/RStudio/bin/pandoc/ (via rmarkdown)
## 
## - Packages ---------------------------------------------------------------------------------------
##  package      * version   date (UTC) lib source
##  ade4         * 1.7-19    2022-04-19 [1] CRAN (R 4.0.5)
##  adegenet     * 2.1.3     2020-05-10 [1] CRAN (R 4.0.4)
##  ape            5.6-2     2022-03-02 [1] CRAN (R 4.0.5)
##  assertthat     0.2.1     2019-03-21 [1] CRAN (R 4.0.4)
##  backports      1.4.1     2021-12-13 [1] CRAN (R 4.0.5)
##  bit            4.0.4     2020-08-04 [1] CRAN (R 4.0.4)
##  bit64          4.0.5     2020-08-30 [1] CRAN (R 4.0.4)
##  boot           1.3-28    2021-05-03 [1] CRAN (R 4.0.5)
##  broom          0.8.0     2022-04-13 [1] CRAN (R 4.0.5)
##  bslib          0.3.1     2021-10-06 [1] CRAN (R 4.0.5)
##  cachem         1.0.6     2021-08-19 [1] CRAN (R 4.0.5)
##  calibrate      1.7.7     2020-06-19 [1] CRAN (R 4.0.4)
##  callr          3.7.0     2021-04-20 [1] CRAN (R 4.0.5)
##  cellranger     1.1.0     2016-07-27 [1] CRAN (R 4.0.4)
##  CKMRpop      * 0.1.3     2021-07-17 [1] CRAN (R 4.0.5)
##  class          7.3-18    2021-01-24 [1] CRAN (R 4.0.4)
##  classInt       0.4-3     2020-04-07 [1] CRAN (R 4.0.4)
##  cli            3.3.0     2022-04-25 [1] CRAN (R 4.0.4)
##  cluster        2.1.1     2021-02-14 [1] CRAN (R 4.0.4)
##  coda           0.19-4    2020-09-30 [1] CRAN (R 4.0.4)
##  codetools      0.2-18    2020-11-04 [2] CRAN (R 4.0.4)
##  colorspace     2.0-3     2022-02-21 [1] CRAN (R 4.0.5)
##  combinat       0.0-8     2012-10-29 [1] CRAN (R 4.0.3)
##  crayon         1.5.1     2022-03-26 [1] CRAN (R 4.0.5)
##  dartR        * 2.0.4     2022-06-05 [1] CRAN (R 4.0.4)
##  data.table     1.14.2    2021-09-27 [1] CRAN (R 4.0.5)
##  DBI            1.1.2     2021-12-20 [1] CRAN (R 4.0.5)
##  dbplyr         2.1.1     2021-04-06 [1] CRAN (R 4.0.5)
##  deldir         0.2-10    2021-02-16 [1] CRAN (R 4.0.4)
##  desc           1.4.1     2022-03-06 [1] CRAN (R 4.0.5)
##  devtools       2.3.2     2020-09-18 [1] CRAN (R 4.0.4)
##  digest         0.6.29    2021-12-01 [1] CRAN (R 4.0.5)
##  dismo          1.3-5     2021-10-11 [1] CRAN (R 4.0.5)
##  doParallel     1.0.17    2022-02-07 [1] CRAN (R 4.0.5)
##  dotCall64      1.0-1     2021-02-11 [1] CRAN (R 4.0.5)
##  dplyr        * 1.0.9     2022-04-28 [1] CRAN (R 4.0.4)
##  e1071          1.7-4     2020-10-14 [1] CRAN (R 4.0.4)
##  ellipsis       0.3.2     2021-04-29 [1] CRAN (R 4.0.5)
##  evaluate       0.15      2022-02-18 [1] CRAN (R 4.0.5)
##  expm           0.999-6   2021-01-13 [1] CRAN (R 4.0.4)
##  fansi          1.0.3     2022-03-24 [1] CRAN (R 4.0.5)
##  fastmap        1.1.0     2021-01-25 [1] CRAN (R 4.0.4)
##  fields         13.3      2021-10-30 [1] CRAN (R 4.0.5)
##  forcats      * 0.5.1     2021-01-27 [1] CRAN (R 4.0.4)
##  foreach        1.5.2     2022-02-02 [1] CRAN (R 4.0.5)
##  fs             1.5.2     2021-12-08 [1] CRAN (R 4.0.5)
##  gap            1.2.3-6   2022-05-13 [1] CRAN (R 4.0.4)
##  gap.datasets   0.0.5     2022-05-09 [1] CRAN (R 4.0.4)
##  gdata          2.18.0.1  2022-05-10 [1] CRAN (R 4.0.4)
##  gdistance      1.3-6     2020-06-29 [1] CRAN (R 4.0.4)
##  gdsfmt         1.26.1    2020-12-22 [1] Bioconductor
##  generics       0.1.2     2022-01-31 [1] CRAN (R 4.0.5)
##  genetics       1.3.8.1.3 2021-03-01 [1] CRAN (R 4.0.4)
##  GGally         2.1.2     2021-06-21 [1] CRAN (R 4.0.5)
##  ggplot2      * 3.3.6     2022-05-03 [1] CRAN (R 4.0.4)
##  glue           1.6.2     2022-02-24 [1] CRAN (R 4.0.5)
##  gmodels        2.18.1.1  2022-05-17 [1] CRAN (R 4.0.4)
##  gridExtra      2.3       2017-09-09 [1] CRAN (R 4.0.4)
##  gtable         0.3.0     2019-03-25 [1] CRAN (R 4.0.4)
##  gtools         3.8.2     2020-03-31 [1] CRAN (R 4.0.3)
##  haven          2.5.0     2022-04-15 [1] CRAN (R 4.0.5)
##  hms            1.1.1     2021-09-26 [1] CRAN (R 4.0.5)
##  htmltools      0.5.2     2021-08-25 [1] CRAN (R 4.0.5)
##  httpuv         1.6.5     2022-01-05 [1] CRAN (R 4.0.5)
##  httr           1.4.3     2022-05-04 [1] CRAN (R 4.0.4)
##  igraph         1.2.11    2022-01-04 [1] CRAN (R 4.0.5)
##  iterators      1.0.14    2022-02-05 [1] CRAN (R 4.0.5)
##  jquerylib      0.1.4     2021-04-26 [1] CRAN (R 4.0.5)
##  jsonlite       1.8.0     2022-02-22 [1] CRAN (R 4.0.5)
##  KernSmooth     2.23-18   2020-10-29 [1] CRAN (R 4.0.4)
##  knitr          1.39      2022-04-26 [1] CRAN (R 4.0.4)
##  later          1.3.0     2021-08-18 [1] CRAN (R 4.0.5)
##  lattice        0.20-41   2020-04-02 [1] CRAN (R 4.0.4)
##  LearnBayes     2.15.1    2018-03-18 [1] CRAN (R 4.0.3)
##  lifecycle      1.0.1     2021-09-24 [1] CRAN (R 4.0.5)
##  lubridate      1.8.0     2021-10-07 [1] CRAN (R 4.0.5)
##  magrittr       2.0.3     2022-03-30 [1] CRAN (R 4.0.5)
##  maps           3.4.0     2021-09-25 [1] CRAN (R 4.0.5)
##  MASS           7.3-53.1  2021-02-12 [1] CRAN (R 4.0.4)
##  Matrix         1.2-18    2019-11-27 [1] CRAN (R 4.0.3)
##  memoise        2.0.1     2021-11-26 [1] CRAN (R 4.0.5)
##  mgcv           1.8-34    2021-02-16 [1] CRAN (R 4.0.4)
##  mime           0.12      2021-09-28 [1] CRAN (R 4.0.5)
##  mmod           1.3.3     2017-04-06 [1] CRAN (R 4.0.4)
##  mnormt         2.0.2     2020-09-01 [1] CRAN (R 4.0.3)
##  modelr         0.1.8     2020-05-19 [1] CRAN (R 4.0.4)
##  munsell        0.5.0     2018-06-12 [1] CRAN (R 4.0.4)
##  mvtnorm        1.1-3     2021-10-08 [1] CRAN (R 4.0.5)
##  nlme           3.1-152   2021-02-04 [1] CRAN (R 4.0.4)
##  patchwork      1.1.1     2020-12-17 [1] CRAN (R 4.0.5)
##  pegas          1.1       2021-12-16 [1] CRAN (R 4.0.5)
##  permute        0.9-7     2022-01-27 [1] CRAN (R 4.0.5)
##  pillar         1.7.0     2022-02-01 [1] CRAN (R 4.0.5)
##  pkgbuild       1.3.1     2021-12-20 [1] CRAN (R 4.0.5)
##  pkgconfig      2.0.3     2019-09-22 [1] CRAN (R 4.0.4)
##  pkgload        1.2.4     2021-11-30 [1] CRAN (R 4.0.5)
##  plyr           1.8.7     2022-03-24 [1] CRAN (R 4.0.5)
##  png            0.1-7     2013-12-03 [1] CRAN (R 4.0.3)
##  PopGenReport   3.0.7     2022-05-27 [1] CRAN (R 4.0.4)
##  prettyunits    1.1.1     2020-01-24 [1] CRAN (R 4.0.4)
##  processx       3.5.0     2021-03-23 [1] CRAN (R 4.0.4)
##  promises       1.2.0.1   2021-02-11 [1] CRAN (R 4.0.4)
##  ps             1.6.0     2021-02-28 [1] CRAN (R 4.0.4)
##  psych        * 2.2.5     2022-05-10 [1] CRAN (R 4.0.4)
##  purrr        * 0.3.4     2020-04-17 [1] CRAN (R 4.0.4)
##  R.methodsS3    1.8.1     2020-08-26 [1] CRAN (R 4.0.3)
##  R.oo           1.24.0    2020-08-26 [1] CRAN (R 4.0.3)
##  R.utils        2.11.0    2021-09-26 [1] CRAN (R 4.0.5)
##  R6             2.5.1     2021-08-19 [1] CRAN (R 4.0.5)
##  raster         3.5-15    2022-01-22 [1] CRAN (R 4.0.5)
##  RColorBrewer   1.1-3     2022-04-03 [1] CRAN (R 4.0.5)
##  Rcpp           1.0.8.3   2022-03-17 [1] CRAN (R 4.0.5)
##  readr        * 2.1.2     2022-01-30 [1] CRAN (R 4.0.5)
##  readxl         1.4.0     2022-03-28 [1] CRAN (R 4.0.5)
##  remotes        2.4.2     2021-11-30 [1] CRAN (R 4.0.5)
##  reprex         2.0.1     2021-08-05 [1] CRAN (R 4.0.5)
##  reshape        0.8.9     2022-04-12 [1] CRAN (R 4.0.5)
##  reshape2       1.4.4     2020-04-09 [1] CRAN (R 4.0.4)
##  rgdal          1.5-32    2022-05-09 [1] CRAN (R 4.0.4)
##  RgoogleMaps    1.4.5.3   2020-02-12 [1] CRAN (R 4.0.4)
##  rlang          1.0.2     2022-03-04 [1] CRAN (R 4.0.5)
##  rmarkdown      2.14      2022-04-25 [1] CRAN (R 4.0.4)
##  rprojroot      2.0.3     2022-04-02 [1] CRAN (R 4.0.5)
##  rstudioapi     0.13      2020-11-12 [1] CRAN (R 4.0.4)
##  rvest          1.0.2     2021-10-16 [1] CRAN (R 4.0.5)
##  sass           0.4.1     2022-03-23 [1] CRAN (R 4.0.5)
##  scales         1.2.0     2022-04-13 [1] CRAN (R 4.0.5)
##  seqinr         4.2-16    2022-05-19 [1] CRAN (R 4.0.4)
##  sessioninfo    1.2.2     2021-12-06 [1] CRAN (R 4.0.5)
##  sf             0.9-7     2021-01-06 [1] CRAN (R 4.0.4)
##  shiny          1.7.1     2021-10-02 [1] CRAN (R 4.0.5)
##  SNPRelate      1.24.0    2020-10-28 [1] Bioconductor
##  sp             1.4-7     2022-04-20 [1] CRAN (R 4.0.5)
##  spam           2.8-0     2022-01-06 [1] CRAN (R 4.0.5)
##  spData         2.0.1     2021-10-14 [1] CRAN (R 4.0.5)
##  spdep          1.1-5     2020-06-29 [1] CRAN (R 4.0.4)
##  StAMPP         1.6.3     2021-08-08 [1] CRAN (R 4.0.5)
##  stringi        1.7.6     2021-11-29 [1] CRAN (R 4.0.5)
##  stringr      * 1.4.0     2019-02-10 [1] CRAN (R 4.0.4)
##  terra          1.5-21    2022-02-17 [1] CRAN (R 4.0.5)
##  testthat       3.0.2     2021-02-14 [1] CRAN (R 4.0.4)
##  tibble       * 3.1.7     2022-05-03 [1] CRAN (R 4.0.4)
##  tidyr        * 1.2.0     2022-02-01 [1] CRAN (R 4.0.5)
##  tidyselect     1.1.2     2022-02-21 [1] CRAN (R 4.0.5)
##  tidyverse    * 1.3.1     2021-04-15 [1] CRAN (R 4.0.5)
##  tmvnsim        1.0-2     2016-12-15 [1] CRAN (R 4.0.3)
##  tzdb           0.3.0     2022-03-28 [1] CRAN (R 4.0.5)
##  units          0.7-0     2021-02-25 [1] CRAN (R 4.0.4)
##  usethis        2.0.1     2021-02-10 [1] CRAN (R 4.0.4)
##  utf8           1.2.2     2021-07-24 [1] CRAN (R 4.0.5)
##  vctrs          0.4.1     2022-04-13 [1] CRAN (R 4.0.5)
##  vegan          2.6-2     2022-04-17 [1] CRAN (R 4.0.5)
##  viridis        0.6.2     2021-10-13 [1] CRAN (R 4.0.5)
##  viridisLite    0.4.0     2021-04-13 [1] CRAN (R 4.0.5)
##  vroom          1.5.7     2021-11-30 [1] CRAN (R 4.0.5)
##  withr          2.5.0     2022-03-03 [1] CRAN (R 4.0.5)
##  xfun           0.30      2022-03-02 [1] CRAN (R 4.0.5)
##  xml2           1.3.3     2021-11-30 [1] CRAN (R 4.0.5)
##  xtable         1.8-4     2019-04-21 [1] CRAN (R 4.0.4)
##  yaml           2.3.5     2022-02-21 [1] CRAN (R 4.0.5)
## 
##  [1] C:/Users/Sidon/OneDrive/Documents/R/win-library/4.0
##  [2] C:/Program Files/R/R-4.0.4/library
## 
## --------------------------------------------------------------------------------------------------
```
