# Supplementary material for: Effective Population Size Estimation in Large Marine Populations: Considering Current Challenges and Opportunities When Simulating Large Data Sets With High‐Density Genomic Information
Source: Evol Appl. 2025 Jul 28;18(8):e70121. doi: 10.1111/eva.70121 (PMC12304085; doi:10.1111/eva.70121)
Supplement: Supplementary file 2 — Data S2. [file EVA-18-e70121-s003.html]

POPSIZE\_Script\_Simulation\_exempleEN


In [1]:

```
from IPython.core.display import display, HTML
display(HTML("<style>.rendered_html { font-size: 12px; }</style>"))
```

## Simulation of demographic and genomic data from two age-structured subpopulations interconnected by gene flow¶

POPSIZE Project (IRD UMR Marbec, CRPMEM de La Réunion, projet FEAMP Mesure 28)

Author: Delord, C. - June 2023

### **Objective** :¶

The POPSIZE project focuses on populations of large marine pelagics. We are working within a relatively simple framework in which we consider two theoretical age-structured sub-populations, where each age class has its own, identical survival and fecundity rate between males and females. The two subpopulations are connected by constant and symmetrical gene flow. Their abundance, in terms of total number of individuals, and their age structure are stable over time and identical between sub-populations.

We aim to simulate the demographic and genetic data specific to these two sub-populations as a simplified representation of a teleost species with high abundance and migratory capacity, like the albacore tuna (*Thunnus alalunga*) for instance.

These simulated data, after post-processing, could be used to assess the ability of several methods to estimate the total abundance (census size Nc) and effective size (Ne) of such populations, despite their high abundance and the potential presence of low but significant spatial genetic substructuring. The methods tested are diverse and can be based on (i) allelic frequency spectra (AFS), such as the tools implemented in moments (Jouganous et al. 2018) or dadi (Gutenkunst et al. 2011]), (ii) on linkage disequilibrium such as the tools implemented in moments-LD (Ragsdale & Gravel 2020), NeEstimator2 (Do et al. 2014) or GONE (Santiago et al. 2020), or (iii) or kinship analyses like the method of *close-kin mark-recapture* (CKMR) (Bravington et al. 2016)).

This document aims at providing a detailed description of the simulation procedure developed to obtain demographic and genetic data on which we can then test these different methods. Whenever possible, additional information is provided via URL links to publications or scientific exchange forums. For further information or for any questions, please feel free to to contact us by email (*chrys.delord@gmail.com*).

### **Caracteristics of the simulated sub-populations** :¶

Before getting into the theoretical concepts and technical aspects of programming, let's start by specifying the demographic and genetic characteristics of the populations we are about to simulate.

Our two sub-populations have the following characteristics:

- The 2 sub-populations each consist of ~17710 individuals in total (parameter ***K***), including 5000 newborns (parameter ***final\_cohort\_size***) generated at each breeding event. These numbers are stable over time, from one breeding event to the next. The age structure is fixed at the start of the simulation (parameter vector ***W***) and each age class is characterized by a value for mortality (parameter vector ***L***) and relative fertility (parameter vector ***B***). The maximum age is set at 15 years (any individual over the age of 15 dies) and the age at maturity is set at 4 years (any individual of age 4 or older can reproduce).
- The 2 subpopulations exchange a continuous and symmetrical proportion of individuals at each time step, set at 5% (parameter ***m***). We therefore consider stable and homogeneous gene flows (abundance ***K*** being equal between the 2 sub-populations) from one sub-population to the other.
- The genetic information of individuals is carried by 5 independent chromosomes of 4e08 base pairs (400 Mbp) each, with a classical recombination rate set at 1e-08 per meiosis and per base pair. This corresponds to a total genome size of 2.0 gigabases. Each of the 5 chromosomes is 400 centimorgans long in genetic distance. Thus, two random loci will be totally independent if they come from two different chromosomes, but will be partially linked if they come from the same chromosome, as is the case for a real dataset with a high density of markers.

### **Choice of a simulation program** :¶

We aim at simulating age-structured sub-populations in order to approximate the biological reality of most large pelagic species, which have overlapping generations. To simulate this biological reality, individual-based simulation tools are necessary (as opposed to sample- or population-based simulation tools such as those based on coalescent model). We have chosen to use the individual-based simulation software *SLiM*. When initializing the simulation, we specify that the simulated populations will not be based on an approximation of the Wright-Fisher population model (as opposed to coalescent-based simulation tools), by activating the ***nonWF*** feature. Individual-based simulation tools have the advantage of enabling biological realism of simulated populations, but have the disadvantage of requiring very long computation times and using a lot of RAM and storage memory, since each individual is modeled separately from the others.

The *SLiM* software can be used to extract various demographic and genealogical information on all or some of the simulated individuals from each simulation. For example, some of these individuals can be sampled according to chosen criteria (e.g., 50 individuals are randomly sampled from each age class and sub-population), and their age and sex recorded, as well as their relationship to other individuals from the same simulation, for example. In addition, activation of the **tree-sequence recording** feature, a key element to be discussed later, also enables gene genealogies within the sample to be recorded.

Beyond realistic demographic information, we want to simulate genomic information. SLiM can generate all or part of this information by simulating chromosomes with or without genetic variability, but this procedure increases computing time considerably. The **tree-sequence recording** feature then comes into play. In cases where only neutral genetic variability is of interest (and therefore depends solely on neutral evolutionary forces and not, a priori, on the fitness of the simulated individuals), it is possible to dispense with the need to simulate this neutral genetic variability with SLiM, and thus save time. At the end of the simulation, only a genealogy, resulting from reproduction and recombination events over time, is exported in the form of a **tree sequence**. This **tree sequence** can then be used as the starting point for a new, more conventional simulation step, based on coalescence, which will integrate neutral genetic variability according to its topology.

The *tskit* and *pyslim* Python libraries link the information contained in a **tree sequence** file from the *SLiM* software to the *msprime* Python library, which enables simulations based on several coalescent models to be carried out, rapidly simulating the past and present evolutionary trajectories of Wright-Fisher populations and the associated genetic diversity. The combination of *SLiM* and *msprime* makes it possible to combine the respective advantages of the individual-centric and coalescent approaches: biological realism and the reconstitution of complex evolutionary trajectories, all in an optimized timeframe (which can nevertheless remain very long in the context of the simulation of genomic information for large populations).

\**The present document will therefore contain sections of code written in the Eidos language specific to* SLiM\*, but also (and mainly) sections written in the Python language.

### **Pre-requisites** :¶

In order to run these simulations, it is essential to install the following libraries and software:

- SLiM version 3.7
- Python3 with *demesdraw*, *math*, *matplotlib.pyplot*, *msprime*, *numpy*, *pandas*, *pyslim* and *tskit* libraries.
- R version >= 4.0.5 with libraries *CKMRpop*, *dartR*, *psych* and *tidyverse* and all their dependencies.

### **Disclaimer**:¶

The code sections below are not intended as a single way of simulating expected data. They may also prove sub-optimal in terms of speed and memory management. Furthermore, as the functionalities of *SLiM*, *pyslim* and *msprime* evolve very rapidly, it is possible that the syntax of certain lines may become obsolete. Finally, all the code sections presented below are inspired by examples found in various places in the scientific and technical literature. We have endeavored to cite as many references and sources as possible, and hope not to have missed any.

## Step 1.¶

## Simulating the contemporary history of sub-populations using an individual-based approach :¶

### SLiM ver. 3.7¶

We start by running the Eidos script in our console (Anaconda in our case) using the command `slim POPSIZE_SLiM_Cohort5000_m005.txt > output_POPSIZE_SLiM_Cohort5000_m005.txt`.

The `POPSIZE_SLiM_Cohort5000_m005.txt` script is written as follows. We will divide this file into several parts to make it easier to read and understand.

Our script begins with a few commented lines (//) providing information and references. We then define some of the simulation functions. Finally, we initialize output text files in which various demographic information will be stored at each simulated time step.

Script `POPSIZE_SLiM_Cohort5000_m005.txt` **(Part 1/4)**

```
// This Eidos script aims to simulate 2 age-structured non-WF subpopulations exchanging gene flows during 100 time steps (= reproductive events) using SLiM37 with the 'pedigree tracking' and 'tree sequence recording' functions.
// It also aims to generate output files containing various information on the simulated individuals. These files are based on the same model as those generated by the Spip simulator and the CKMRpop R library (Anderson et al. 2021).
// This script performs several sampling events at various time-steps ('serial sampling') along with the function 'treeSeqRememberIndividuals()'.
// This script was built based on various resources as listed below :
//     - the CKMRpop vignette : https://eriqande.github.io/CKMRpop/articles/using-other-simulation-programs.html associate to the paper from Anderson et al. (2021) (https://doi.org/10.1111/1755-0998.13513).
//     - the 'SLiM recipe' 16.2 (SLiM User Manuel with last update from February 2022)
//     - the 'SLiM recipe' 16.5 (SLiM User Manuel with last update from February 2022)
//     - section 4.1.6 from the SLiM User Manuel with last update from February 2022 
//     - the following discussion: https://groups.google.com/g/slim-discuss/c/PaCARghZy9Q
//     - the msprime vignette: https://tskit.dev/msprime/docs/stable/ancestry.html?highlight=multiple#multiple-chromosomes

initialize() {

    setSeed(getSeed());    
    initializeSLiMModelType("nonWF"); // the simulation lies outside the Wright-Fisher assumptions.
    initializeSLiMOptions(keepPedigrees = T); // we want to record the pedigree of individuals.
    initializeTreeSeq(); // activation of tree-sequence recording.
    initializeSex("A"); // separate sexes.

    initializeMutationType("m1", 0.5, "f", 0.0);
    initializeGenomicElementType("g1", m1, 1.0); // one unique and homogeneous type of genomic element and mutation feature.
    initializeGenomicElement(g1, 0, 1999999999); // one "chromosome" of moderate size of 2 Gb.
    initializeMutationRate(0.0); // no genetic variation will be simulated at this stage. Neutral mutations will be added during the pyslim/msprime simulation phase.
    initializeRecombinationRate(c(1.0e-08, 0.5, 1.0e-08, 0.5, 1.0e-08, 0.5, 1.0e-08, 0.5, 1.0e-08), c(400000000, 400000001, 800000000, 800000001, 1200000000, 1200000001, 1600000000, 1600000001, 1999999999)); 
    // we cut our "chromosome" in 5 freely-recombinating sections of equal size (c.f 'SLiM recipe' 6.1.4)
    // recombinaison rate within each section is 1.0e-08.
    m1.convertToSubstitution = T;

    defineConstant("K", 17710); // total abondance (census size) of each sub-population directly from generation 1 (including newly generated individuals -newborns), all age classes included. This value will stay constant (give or take a few individuals) through time.
    // we define mortality rates L and relative fecundity B for each age class from 0 to 15. All individuals of age zero (newborns) survive to age 1:
    defineConstant("L", c(0.00, 0.46, 0.38, 0.34, 0.31, 0.29, 0.31, 0.34, 0.38, 0.44, 0.55, 0.55, 0.55, 0.55, 0.60, 1.00));
    defineConstant("B", c(0.00, 0.00, 0.00, 0.00, 0.10, 0.20, 0.30, 0.40, 0.50, 0.60, 0.70, 0.80, 0.90, 1.00, 1.10, 1.20));
    defineConstant("W", c(25000.00, 25000.00, 13500.00, 8370.00, 5524.00, 3811.70, 2706.31, 1867.35, 1232.45, 764.12, 427.91, 192.56, 86.65, 38.99, 17.55, 7.02)); // we define the relative proportions W of the different age classes (the raw values are of little importance, only their relative proportions).
    defineConstant("m", 0.05); // symmetrical migration rate m between sub-populations.

    // initialization of some output files to store information from simulated individuals:
    // those files are homologous to those generated by the Spip software used by the R librairy CKMRpop.
    writeFile("SLiM_prekill_census.tsv", "year\tpop\tage\tmale\tfemale");
    writeFile("SLiM_postkill_census.tsv", "year\tpop\tage\tmale\tfemale");
    writeFile("SLiM_demo_table.tsv", "generation\tgen_time\tkbar\tvk\tne_demo");
    writeFile("SLiM_samples.tsv", "ID\tsyears_pre\tpop_pre\tsyears_post\tpop_post\tsyears_dur\tpop_dur");
    writeFile("SLiM_ancestries.tsv", "ID\tancestors");  

    // initialization of an empty dictionary object to store the information needed to calculate the average generation time and variance in lifetime reproductive output between individuals. This information will be used to obtain the effective demographic size, per generation, of the two simulated sub-populations.
    defineGlobal("n_offspring", Dictionary());
}
```

After this initialization phrase, we will need to define the modalities of the reproduction events (which ensure the transition from one time step to the next). This is done in a callback section *reproduction()*. A male and female breeder are sampled with a probability that depends on their age and the vector parameter ***B***. The pair generates a non-zero number of offspring according to a Poisson distribution with parameter 2.3. The parents sampling process continues until 5000 newborns have been generated. At this stage, stable subpopulation size is not an emergent property of the model. Although it would be more elegant if it were, this does not affect the validity of the demographic and genomic information generated.

In this same "callback" *reproduction()*, the "n\_offspring" dictionary gradually stores information on the total number of offspring generated, over the course of their existence, by 11 successive cohorts. A function deduces the effective demographic size per generation and the average generation time. This information is exported in the file `SLiM_demo_table.tsv`.

Script `POPSIZE_SLiM_Cohort5000_m005.txt` **(Part 2/4)**

```
reproduction() { 

    for (subpop0 in sim.subpopulations) {
        fecundity = B[subpop0.individuals.age];
        subpop0.individuals.tagF = fecundity;
        potential_dads = subpop0.individuals[subpop0.individuals.age > 0 & subpop0.individuals.sex == "M"];
        potential_moms = subpop0.individuals[subpop0.individuals.age > 0 & subpop0.individuals.sex == "F"];
        current_cohort_size = c(0);
        final_cohort_size = 5000;

        while (current_cohort_size < final_cohort_size) {
            dad = sample(potential_dads, size = 1, replace = F, weights = potential_dads.tagF);
            mom = sample(potential_moms, size = 1, replace = F, weights = potential_moms.tagF);
            litterSize = 0;
            do { 
                litterSize = rpois(1, 2.3);
            }
            while (litterSize == 0);

            if (sim.generation < 28) {
                vec_mom = n_offspring.getValue(format('%d', mom.pedigreeID));
                vec_mom[1] = asFloat(((vec_mom[0] * vec_mom[1]) + (asFloat(litterSize) * asFloat(mom.age))) / (vec_mom[0] + asFloat(litterSize)));
                vec_mom[0] = asFloat(vec_mom[0] + asFloat(litterSize));
                n_offspring.setValue(format('%d', mom.pedigreeID), vec_mom);
                vec_dad = n_offspring.getValue(format('%d', dad.pedigreeID));
                vec_dad[1] = asFloat(((vec_dad[0] * vec_dad[1]) + (asFloat(litterSize) * asFloat(dad.age))) / (vec_dad[0] + asFloat(litterSize)));
                vec_dad[0] = asFloat(vec_dad[0] + asFloat(litterSize));
                n_offspring.setValue(format('%d', dad.pedigreeID), vec_dad);
            }

            for (j in seqLen(litterSize)) {
                offspring = subpop0.addCrossed(mom, dad);
                offspring.tag = subpop0.id;
                if (sim.generation < 28) {                
                    n_offspring.setValue(format('%d', offspring.pedigreeID), c(0.0, 0.0, asFloat(sim.generation)));         
                }
            }
            current_cohort_size = current_cohort_size + litterSize;
        }               
    }       

    // Calculation of the demographic effective size and generation time for the cohort born 15 years ago (al sub-populations confounded):
    if (sim.generation > 16 & sim.generation < 28) { // this calculation is performed on 11 successive cohorts, born from time-step 2 to time-step 12, and only when all the individuals from the same cohort have died and can no longer reproduce (i.e. 15 time steps after cohort birth).
        test0 = c(0.0, 0.0, 0.0);
        for (i in unique(n_offspring.allKeys)) {
            if (n_offspring.getValue(i)[2] == asFloat(sim.generation - 15)) {
                test0 = rbind(test0, n_offspring.getValue(i));
                n_offspring.setValue(i, NULL);
            }
        }
        lifetime_kbar = mean(test0[1:nrow(test0)-1, 0]);
        lifetime_vk = var(c(test0[1:nrow(test0)-1, 0]));
        gen_time = sum(test0[, 0]*test0[, 1])/sum(test0[, 0]); // generation time
        ne_demo = (4*nrow(test0)*gen_time)/(lifetime_vk+2); // demographic effective size, calculated based on lifetime variance in reproductive success
        line = paste(c(sim.generation - 15, gen_time, lifetime_kbar, lifetime_vk, ne_demo), sep = "\t");
        writeFile("SLiM_demo_table.tsv", line, append=T);            
    }

    if (sim.generation == 28) { // deactivation of the 'n_offspring' dictionary after time-step 28.
        // This enable to release some memory and increase simulation speed.
        rm(variableNames="n_offspring");    
    }

    self.active = 0; // the reproduction() callback deactivates itself the number of newborns reaches 5000.
}
```

In the *1 early()* callback section (dedicated to time step 1, that is, the beginning of the simulation), we directly initialize both subpopulations at total abundance **K**, "filling" each age class according to the set age structure. Besides, the *early()* sections allow symmetrical migration between sub-populations and the recording of precise numbers per age class before mortality occurs.

Script `POPSIZE_SLiM_Cohort5000_m005.txt` **(Part 3/4)**

```
1 early() { // for time-step 1 (beginning of the simulation).

    sim.addSubpop("p1", K);
    sim.addSubpop("p2", K);
    p1.individuals.age = sample(seq(0, 15), size = K, replace = T, weights = W); // filling of sub-population p1.
    p2.individuals.age = sample(seq(0, 15), size = K, replace = T, weights = W); // filling of sub-population p2.
    p1.individuals.tag = 1;
    p2.individuals.tag = 2;

    for (i in c(p1.individuals.pedigreeID, p2.individuals.pedigreeID)) {
        n_offspring.setValue(format('%d', i), c(0.0,0.0,1.0));
    }
}

early() { // for each time-step, before mortality occurs.

    // a number of migrants will be picked according to the m parameter:
    nIndividuals = sum(sim.subpopulations.individualCount);
    nMigrants = rpois(1, nIndividuals * m);
    migrants = sample(sim.subpopulations.individuals, nMigrants);

    for (migrant in migrants) {
        do dest = sample(sim.subpopulations, 1);
        while (dest == migrant.subpopulation);
        dest.takeMigrants(migrant);
    }    

    // records the abundance within each age class, before mortality occurs, in the output file "SLiM_prekill_census.tsv":
    for (subpop in sim.subpopulations) {    
        print(tabulate(subpop.individuals.age, maxbin=15));
        inds = subpop.individuals;
        ages = inds.age;
        age_bins = 0:15;  // age categories, 0 to 15
        male_ages = ages[inds.sex == "M"];
        female_ages = ages[inds.sex == "F"];
        m_census = tabulate(male_ages, maxbin = 15);
        f_census = tabulate(female_ages, maxbin = 15);      

        for(a in age_bins) {
            line = paste(sim.generation, subpop.id, a, m_census[a], f_census[a], sep = "\t");
            writeFile("SLiM_prekill_census.tsv", line, append=T); // effectifs par classe d'âge avant mortalité.
        }        

        mortality = L[ages];
        survival = 1 - mortality;
        inds.fitnessScaling = survival;
    }
}
```

The *late()* callbacks allow us to record the precise number of individuals per age class after mortality has occurred.

More importantly, they also allow us to perform "serial sampling" (i.e. sampling individuals over several successive time steps) of individuals during the last 11 time steps (90 to 100) of the simulation. More precisely, this involves storing genetic and demographic information on a proportion of individuals (which will constitute the sample) within each simulated sub-population. Here, we sample a proportion of 10% of each age class (`ns = rbinom(1, num_1_to_15, 0.10);`) (which, it should be noted, would be much more laborious for population sizes larger than 17710, for which we would then have to reduce this proportion). It should also be noted that this sampling is discounted: i.e., a sampled individual does not disappear from the population, and could be resampled at a later time step by chance, as in a capture-mark-recapture protocol. If necessary, this can be taken into account during post-processing of simulated data, as each individual has a unique identifier, which is retained when exporting demographic information in the output files.

Each sampled individual will have its demographic information (e.g. birth time step, age(s)/place(s)/time step of sampling) saved within the `SLiM_samples.tsv` file. By enabling pedigree recording, we can also save the identifiers of the parents and grandparents of each sampled individual, within the `SLiM_ancestries.tsv` file. In addition, the genealogies corresponding to the sampled individuals are preserved by activating the function `sim.treeSeqRememberIndividuals();`. This will enable genetic information to be assigned to these individuals in the next coalescent-based simulation phase. Without this option, only individuals present at the very last simulated time step are guaranteed to be exported with a complete genealogy. In serial sampling, as performed here, the `sim.treeSeqRememberIndividuals();` function is therefore very important.

Script `POPSIZE_SLiM_Cohort5000_m005.txt` **(Part 4/4)**

```
late() {
    // records the abundance within each age class, after mortality occurs, in the output file "SLiM_postkill_census.tsv":
    for (subpop in sim.subpopulations) {
        inds = subpop.individuals;
        ages = inds.age;
        age_bins = 0:15;  // age categories, 0 to 15
        male_ages = ages[inds.sex == "M"];
        female_ages = ages[inds.sex == "F"];
        m_census = tabulate(male_ages, maxbin = 15);
        f_census = tabulate(female_ages, maxbin = 15);

        for(a in age_bins) {
            line = paste(sim.generation, subpop.id, a, m_census[a], f_census[a], sep = "\t");
            writeFile("SLiM_postkill_census.tsv", line, append=T);  // abundance per age class after mortality.
        }
    }
}

90:100 late() {
    for (subpop in sim.subpopulations) {
        num_1_to_15 = sum(tabulate(subpop.individuals.age, maxbin = 15)[1:15]);

        ns = rbinom(1, num_1_to_15, 0.10); // we collect the demograpic and genetic information of some of the simulated individuals (10% per age-class -this will constitute our sample). 
        samps = subpop.sampleIndividuals(ns, minAge = 1, maxAge = 15);
        sim.treeSeqRememberIndividuals(samps); // SLiM will save the gene genealogy of the sampled individuals.

        for(s in samps) {
            s_name = paste0(s.sex, sim.generation - s.age, "_", s.tag, "_", s.pedigreeID);
            line = paste(s_name, "", "", sim.generation, subpop.id, "", "", sep = "\t");
            writeFile("SLiM_samples.tsv", line, append = T);        
            s_anc = c(s.pedigreeID, s.pedigreeParentIDs, s.pedigreeGrandparentIDs);
            s_anc = s_anc[c(0, 2, 1, 6, 5, 4, 3)];
            s_anc_commas = paste(s_anc, sep = ",");
            line = paste(s_name, s_anc_commas, sep = "\t");
            writeFile("SLiM_ancestries.tsv", line, append = T); // stores some pedigree information from the sampled individuals (i.e., IDs of their parents and grands-parents).
        }
    }
}

100 late() { // finally, for the ast simulated time-step, we export the "tree sequence" containing the gene genealogy of all individuals that are currenty alive, but also of all individuals that were sampled across time-steps 90 to 100 thanks to the function 'sim.treeSeqRememberIndividuals();'.
    sim.treeSeqOutput("POPSIZE_SLiM_Cohort5000_m005.trees");
}

// End of Eidos script.
```

The final file `POPSIZE_SLiM_Cohort5000_m005.trees` is our main output for the moment. Together with the `SLiM_demo_table.tsv` file, it will enable us to move on to step 2, i.e. the reconstruction of the past evolutionary trajectory (i.e. prior to the 100 time steps simulated using SLiM software) of our two sub-populations. This reconstitution is based on the topology of the **tree sequence** exported via SLiM and using a coalescent approach implemented in the Python libraries *pyslim* and *msprime*, a process called **recapitation**. We will now leave the Eidos programming language and use the Python console.

## Step 2.¶

## Simulation of past evolutionary trajectory of subpopulations using a coalescent-based approach:¶

### pyslim ver. 0.700 et msprime ver. 1.2.0¶

Now we launch the `POPSIZE_pyslim_Cohort5000_m005.py` script using the command `python POPSIZE_pyslim_Cohort5000_m005.py > output_POPSIZE_pyslim_Cohort5000_m005.txt`.

This script `POPSIZE_pyslim_Cohort5000_m005.py` writes as follows. We described this file into several parts to make it easier to read and understand.

Script `POPSIZE_pyslim_Cohort5000_m005.py` **(Part 1/7)**

The first step is to import all the necessary tools into our working environment.
Then, we import our main work file: the **tree sequence** file from step 1. The `print(ts)` function allows us to view some information about this **tree sequence**. We find around 2.6e06 distinct genealogies ('trees') and the 2.0 Gb size of the simulated genome. The **tree sequence** contains 36,521 individuals, including all surviving individuals from the very last time step simulated in SLiM, as well as previously sampled individuals who have died, but whose information has been preserved by the `sim.treeSeqRememberIndividuals();` function.

In [1]:

```
# Import Python libraries:
import datetime, demesdraw, functools, itertools, math, msprime, os, pyslim, tskit  
import matplotlib.pyplot as plt
import numpy as np
import pandas as pd
import statistics as s

# Simulation parameters: 
str1 = "5000"
str2 = "m005"
migrate = 0.05

print('Hi! You are currently using pyslim ver ', pyslim.__version__, ', tskit ver ', tskit.__version__, ' and msprime ver ', msprime.__version__, '. See you soon.', sep='')
current_day = datetime.datetime.now().strftime("%Y%m%d")

# Import the "tree sequence" obtained from Step 1:
# os.getcwd()
# os.chdir('C:\\Users\\Sidon\\OneDrive\\Documents\\SLiM_5000_m005_runfiles')
ts = pyslim.load("POPSIZE_SLiM_Cohort%s_%s.trees" % (str1, str2))
print(ts)
```

```
Hi! You are currently using pyslim ver 0.700, tskit ver 0.4.1 and msprime ver 1.2.0. See you soon.
╔══════════════════════════╗
║TreeSequence              ║
╠═══════════════╤══════════╣
║Trees          │   2562579║
╟───────────────┼──────────╢
║Sequence Length│2000000000║
╟───────────────┼──────────╢
║Time Units     │     ticks║
╟───────────────┼──────────╢
║Sample Nodes   │     73042║
╟───────────────┼──────────╢
║Total Size     │ 209.4 MiB║
╚═══════════════╧══════════╝
╔═══════════╤═══════╤═════════╤════════════╗
║Table      │Rows   │Size     │Has Metadata║
╠═══════════╪═══════╪═════════╪════════════╣
║Edges      │5248573│160.2 MiB│          No║
╟───────────┼───────┼─────────┼────────────╢
║Individuals│  36521│  3.5 MiB│         Yes║
╟───────────┼───────┼─────────┼────────────╢
║Migrations │      0│  8 Bytes│          No║
╟───────────┼───────┼─────────┼────────────╢
║Mutations  │      0│  1.2 KiB│          No║
╟───────────┼───────┼─────────┼────────────╢
║Nodes      │ 157113│  5.7 MiB│         Yes║
╟───────────┼───────┼─────────┼────────────╢
║Populations│      3│  2.3 KiB│         Yes║
╟───────────┼───────┼─────────┼────────────╢
║Provenances│      1│ 12.3 KiB│          No║
╟───────────┼───────┼─────────┼────────────╢
║Sites      │      0│ 16 Bytes│          No║
╚═══════════╧═══════╧═════════╧════════════╝
```

Script `POPSIZE_pyslim_Cohort5000_m005.py` **(Part 2/7)**

We import the `SLiM_demo_table.tsv` file, which will enable us to extract the effective demographic size calculated per generation in step 1 for each sub-population. We check that the number of individuals still alive at the last time step simulated in SLiM (amounting to 25435 according to the file `SLiM_postkill_census.tsv` for time step 100) is indeed found in our **tree sequence** at time step 0 (`ts.individuals_alive_at(0)`), which indeed gives us a total of 12692+12743 = 25435 individuals.

Finally, we visualize the distribution of coalescence times along the simulated genome. In just 100 timesteps simulated with SLiM, it is highly unlikely that TMRCA could have been reached anywhere, and we expect the size of the genealogies, in number of timesteps, to be homogeneous and correspond to the number of timesteps simulated.

In [2]:

```
# Import file "SLiM_demo_table.tsv" from Step 1:
SLiM_demo = pd.read_table("SLiM_demo_table.tsv", sep ='\t', header = 0, index_col=False)

# Visualize present time populations (0 generations ago, corresponding to the last simulated time-step in SLiM):
alive = ts.individuals_alive_at(0)
num_alive = [0 for _ in range(ts.num_populations)]
for i in alive:
  ind = ts.individual(i)
  num_alive[ind.population] += 1

for pop, num in enumerate(num_alive):
  print(f"Nombre d'individus au temps présent dans la population {pop}: {num}") # Number of individuals per sub-population at present time.

# Calculing the size of coalescent trees (in generations) (tree heights):
def tree_heights(ts):
    heights = np.zeros(ts.num_trees + 1)
    for tree in ts.trees():
        if tree.num_roots > 1: # we did not reach MRCA yet.
            heights[tree.index] = ts.slim_generation
        else:
            children = tree.children(tree.root)
            real_root = tree.root if len(children) > 1 else children[0]
            heights[tree.index] = tree.time(real_root)
    heights[-1] = heights[-2]
    return heights

# Visualize the size of coalescent trees (in generations) before recapitation (it should be the same for all loci: y=number of time-steps simulated in SLiM):
breakpoints = list(ts.breakpoints())
heights = tree_heights(ts)
plt.step(breakpoints, heights, where='post')
plt.show()
plt.savefig('pyslim_tree_height0_Cohort%s_%s.png' % (str1, str2))
```

```
Nombre d'individus au temps présent dans la population 0: 0
Nombre d'individus au temps présent dans la population 1: 12692
Nombre d'individus au temps présent dans la population 2: 12743
```

```
/home2/datawork/cdelord/conda-env/simupop-slim/lib/python3.7/site-packages/pyslim/slim_tree_sequence.py:36: FutureWarning: The SlimTreeSequence class is being phased out, as most important functionality is provided by tskit. Please see the `documentation <https://tskit.dev/pyslim/latest/previous_versions.html>`_. Please access ts.metadata['SLiM']['generation'] instead.
  FutureWarning
```

```
<Figure size 432x288 with 0 Axes>
```

Script `POPSIZE_pyslim_Cohort5000_m005.py` **(Part 3/7)**

The recapitation phase will enable us to complete the gene genealogies present in our **tree sequence**, i.e. to ensure that TMRCA is reached throughout the simulated genome. To do this, we need to provide *pyslim* with information on the present and past demographics of the simulated sub-populations. We will do this using the module `msprime.Demography.from_tree_sequence(ts)`.

In order to maintain a constant effective size throughout the chronology (which isn't compulsory and won't always be the case, but is simpler for the present example), we will choose as our starting point (the present time), the demographic effective size we calculated over several successive cohorts in step 1. The evolutionary trajectory simulated here for the past of our two subpopulations will be very simple: the two subpopulations have always maintained a constant effective size since their divergence. They once constituted a single ancestral population, whose effective size corresponded to the sum of their local effective sizes. All this can be visualized using the representations obtained via the `demography.debug()` module.

A simple harmonic mean calculation from `SLiM_demo_table.tsv` indicates that the demographic effective size is 2772 per sub-population. Generation time is estimated at 6.989.

Before going any further, we must mention a **crucial** point concerning the combination of **nonWF** simulations with *SLiM*, and Wright-Fisher (coalescent) simulations with *pyslim*/*msprime*. This essential point of caution is underlined by the warning displayed in red at the output of this section of code. Indeed, the unit of time considered by *SLiM* and *pyslim*/*msprime* is not the same: it corresponds to a time step/reproduction cycle/"tick" in the first case, and to a Wright-Fisher generation in the second. We therefore need to re-scale some parameters (at least effective size, mutation and recombination rates) to take account of this and ensure a good combination of individual-centered and coalescent-based simulations. A detailed discussion of this is available in the Time Units section of the *pyslim* online manual. Further useful information can be found in this direct exchange with the library developers: Seeking for best practices with hybrid "backward-forward" simulation.

We can now continue:

- **Precisions on the effective size *Ne* to define in `pop.initial_size`:**

The value of 2772 corresponds to the effective size, per generation and per sub-population, calculated from demographic data simulated in *SLiM*. It takes generation time into account, and is different from the effective size calculated per reproductive cycle, which would correspond to a value of *Nb* (effective number of breeders). Insofar as the two simulated populations present an equal and constant effective size over time, and insofar as these two sub-populations exchange constant and symmetrical gene flows, we should be able to consider that the global effective size corresponds to the sum of the local effective sizes (e.g., Wang & Caballero, 1999). This is why, here, we obtain `Ne_demo` by dividing the global effective size value from `SLiM_demo_table.tsv` by 2. To re-scale the effective size to take account of the time unit difference between *SLiM* and *pyslim*/*msprime*, we need to multiply `Ne_demo` by the generation time `gen_time`. This gives us the value `Ne_pyslim` to be entered in `pop.initial_size`.

In addition, our demographic effective size `Ne_demo` (which is a type of variance effective size) is therefore used here as a proxy for eigenvalue effective size (which is an asymptotic value of effective size towards which variance and inbreeding effective sizes converge at equilibrium). Unless critical demographic events occur during the evolutionary trajectory of the subpopulations, we should be able to consider that this demographic / eigenvalue effective size is also equivalent to the coalescent effective size (Ryman et al. 2019) and therefore usable in the demographic model used here in *pyslim* once rescaled (e.g., `Ne_pyslim`). This approximation is made possible by the extreme simplicity of the evolutionary trajectory simulated here.

However, if we wished to simulate contemporary demographic events (e.g. bottleneck or overfishing-related decline in abundance), or past events (e.g. ancestral population growth), or if we wished to model distinct dynamics between the two subpopulations (e.g. decline in abundance of one subpopulation while the second remains stable), then we would have to reconsider all this. The effective demographic size would probably have to be calculated in a different way in step 1 of *SLiM*. This effective size would no longer necessarily be equivalent to the coalescence effective size, and we would need to think about how best to model the evolutionary trajectory of sub-populations in the `msprime.Demography.from_tree_sequence(ts)` module of *pyslim*, integrating this contemporary effective size, its fluctuations going back in time, and its eventual transition to the coalescence effective size.

See also: Questions about treeseq good practices

- **Precisions on migration rates *migrate* to define in `demography.set_symmetric_migration_rate`:**

In our case, where migration flows are symmetrical between two subpopulations of identical effective size and abundance, we can keep the same migration parameter value *m* and *migrate* between step 1 *SLiM* and step 2 *pyslim*. This is despite the fact that the definition of the migration parameter is not strictly identical between the two programs (in *SLiM*, it's the average proportion of individuals from one subpopulation that migrate to another, whereas in *pyslim*/*msprime*, it's the proportion of haploid individuals from one subpopulation with a parent from another subpopulation). Here again, if we wished to simulate subpopulations of different sizes and/or with asymmetrical migration flows, we would have to set the migration flows differently between the *SLiM* and *pyslim* stages respectively.

See also: Coalescence trajectory for non symmetric migration
See also: msprime doc > Demographic Models > Model > Definitions

- **Precisions on recombination rates to define in `pyslim.recapitate()`:**

Like effective size and mutation rate (c.f. Part 6/7), recombination rates must be rescaled to take account of the difference in time units considered by *SLiM* and *pyslim*. The Wright-Fisher recombination rate per generation is obtained by dividing the recombination rate set in *SLiM* (expressed per time step/breeding cycle/meiosis), by the generation time. Here, `r_chrom` = 1e-08 / 6.98 = 1.435e-9. The same calculation applies to the mutation rate (c.f. Part 6/7).

In [3]:

```
# -------------------------------------------------------------------------------------------
# RECAPITATION (here, we need to specify every demographic event that occurred during the past history of the subpopulations, as well as their effective size, migration flows and recombination rates):
# -------------------------------------------------------------------------------------------

Ne_demo = (s.harmonic_mean(SLiM_demo['ne_demo']))/2 # Effective size per sub-population.
# Demographic effective size Ne_demo will be used as a proxy of coalescence effective size.
gen_time = (s.harmonic_mean(SLiM_demo['gen_time']))
print("Mean, per-generation effective size obtained from SLiM reproductive outputs is ", Ne_demo, ". Generation time is ", gen_time, ".")

Ne_pyslim = Ne_demo*gen_time # Re-scaling of the effective size per Wright-Fisher generation per sub-population. 
t_split = 4*Ne_pyslim # The divergence time between subpopulations is set to favor migration-drift equilibrium.

# Initialization of recombinaison rates chromosome section: 
r_chrom = 1.435e-9 # Re-scaling of the recombination rate per Wright-Fisher generation. 
r_break = math.log(2)
chrom_positions = [0, 400000000, 800000000, 1200000000, 1600000000, 2000000000]
map_positions = [chrom_positions[0], chrom_positions[1], chrom_positions[1] + 1, chrom_positions[2], chrom_positions[2] + 1, chrom_positions[3], chrom_positions[3] + 1, chrom_positions[4], chrom_positions[4] + 1, chrom_positions[5]]
rates = [r_chrom, r_break, r_chrom, r_break, r_chrom, r_break, r_chrom, r_break, r_chrom]
rate_map = msprime.RateMap(position=map_positions, rate=rates)

# Definition of past evolutionary trajectory: 
demography = msprime.Demography.from_tree_sequence(ts)
for pop in demography.populations:
    if pop.name in ('p1', 'p2'):
        pop.initial_size = Ne_pyslim # Effective size of current sub-populations.
    else:
        pop.initial_size = 2*Ne_pyslim # Effective size of ancestral population "pop_0", before divergence.
# demography.add_population(name="pop_anc", initial_size= int(Ne_pyslim*2)) 
# the commented line above would generate a bug as described here : https://groups.google.com/g/slim-discuss/c/WA-c1hUWgIY.
# In reality, to insert a new, non-existent population into the tree sequence, we have to initialize its metadata manually, as explained by P.Ralph in the URL above.
# Here, we finally take advantage of the empty population "pop_0" automatically present in the tree sequence. It will become our ancestral population.
demography.add_population_split(time=t_split, derived=["p1", "p2"], ancestral="pop_0") # Divergence between sub-populations.
demography.set_symmetric_migration_rate(populations=["p1", "p2"], rate=migrate) # Setting up gene flow between subpopulations.   

debug = demography.debug() # This module allows us to visualize the evolutionary trajectory modeled above for verification.
print(debug)
mod = demography.to_demes()
ax = demesdraw.tubes(mod)

# WE CAN NOW PROCEED WITH RECAPITATION:
rts = pyslim.recapitate(ts, demography=demography, recombination_rate=rate_map, random_seed=1)
```

```
Mean, per-generation effective size obtained from SLiM reproductive outputs is  2772.0601826540315 . Generation time is  6.988647213350303 .
DemographyDebugger
╠═════════════════════════════════════╗
║ Epoch[0]: [0, 7.75e+04) generations ║
╠═════════════════════════════════════╝
╟    Populations (total=3 active=2)
║    ┌──────────────────────────────────────────────────────┐
║    │    │     start│       end│growth_rate  │  p1  │  p2  │
║    ├──────────────────────────────────────────────────────┤
║    │  p1│   19373.0│   19373.0│ 0           │  0   │ 0.05 │
║    │  p2│   19373.0│   19373.0│ 0           │ 0.05 │  0   │
║    └──────────────────────────────────────────────────────┘
╟    Events @ generation 7.75e+04
║    ┌─────────────────────────────────────────────────────────────────────────────────────┐
║    │       time│type        │parameters         │effect                                  │
║    ├─────────────────────────────────────────────────────────────────────────────────────┤
║    │  7.749e+04│Population  │derived=[p1, p2],  │Moves all lineages from derived         │
║    │           │Split       │ancestral=pop_0    │populations 'p1' and 'p2' to the        │
║    │           │            │                   │ancestral 'pop_0' population. Also set  │
║    │           │            │                   │the derived populations to inactive,    │
║    │           │            │                   │and all migration rates to and from     │
║    │           │            │                   │the derived populations to zero.        │
║    └─────────────────────────────────────────────────────────────────────────────────────┘
╠═══════════════════════════════════════╗
║ Epoch[1]: [7.75e+04, inf) generations ║
╠═══════════════════════════════════════╝
╟    Populations (total=3 active=1)
║    ┌───────────────────────────────────────────┐
║    │       │     start│       end│growth_rate  │
║    ├───────────────────────────────────────────┤
║    │  pop_0│   38745.9│   38745.9│ 0           │
║    └───────────────────────────────────────────┘
```

```
/home2/datawork/cdelord/conda-env/simupop-slim/lib/python3.7/site-packages/msprime/ancestry.py:831: TimeUnitsMismatchWarning: The initial_state has time_units=ticks but time is measured in generations in msprime. This may lead to significant discrepancies between the timescales. If you wish to suppress this warning, you can use, e.g., warnings.simplefilter('ignore', msprime.TimeUnitsMismatchWarning)
  warnings.warn(message, TimeUnitsMismatchWarning)
```

Script `POPSIZE_pyslim_Cohort5000_m005.py` **(Part 4/7)**

Following our recapitulation phase, we need to check that the gene genealogies in our **tree sequence** have, this time, found their TMRCA, and therefore that coalescence is complete throughout the genome. We can again visualize the distribution of coalescence times along the genome and note that "the average coalescence time, divided by 4, gives 39747.89704852677", which corresponds roughly to the effective size of the ancestral population (and the sum of the effective sizes of the sub-populations) as set in *pyslim*.

The `print(rts)` function allows us to visualize some information about this **tree sequence** after recapitation. Compared to the previous version, it contains denser trees (value `Edges` and `Nodes`) and more genealogies.

In [4]:

```
# Displaying tree sequence information, after recapitation.
print(rts)
assert(max([t.num_roots for t in rts.trees()]) == 1)

# Visualize the size of coalescent trees (in generations) after recapitation:
# It should be variable between loci now, because these do not have the same coalescence time (TMRCA).
breakpoints = list(rts.breakpoints())
heights = tree_heights(rts)
print('Le temps moyen de coalescence, divisé par 4, donne ', np.mean(heights)/4)
plt.step(breakpoints, heights, where='post')
plt.show()
plt.savefig('pyslim_tree_height1_Cohort%s_%s.png' % (str1, str2))

# We check that after recapitation, all trees now share a common root.
orig_max_roots = max(t.num_roots for t in ts.trees())
recap_max_roots = max(t.num_roots for t in rts.trees())
print(f"Nombre de racines distinctes avant récapitation: {orig_max_roots}\n"
      f"Nombre de racines distinctes après récapitation: {recap_max_roots}")
rts.dump("POPSIZE_pyslim_Cohort%s_%s_rts.trees" % (str1, str2))

# Visualization of coalescence rates through time:
def count_tree_coalescence_pairs(ts, epochs):
    nepochs = len(epochs)
    times = dict()
    for tr in ts.trees():
        for node in tr.nodes():
            if tr.is_leaf(node):
                continue
            nlineages = np.array([tr.num_samples(child) for child in tr.children(node)])
            ns = functools.reduce(lambda s, t: s + t, (x*y for x, y in itertools.combinations(nlineages, 2))) 
            t = tr.time(node)
            times[t] = ns + times.get(t, 0) 
    counts = np.fromiter(times.values(), dtype=int)
    times = np.fromiter(times.keys(), dtype=float)
    indices = np.digitize(times, epochs)
    coalesced = np.zeros(shape=nepochs) 
    np.add.at(coalesced, indices, counts)
    total = np.cumsum(coalesced[::-1])[::-1]    
    #counts = count_tree_coalescence_pairs(mts, bins)
    plt.figure(1)
    plt.plot(epochs[:-1], coalesced[1:])
    plt.xlabel("Nombre de générations avant présent (en bins de 100 générations)") # Number of generations before present (in bins of 100 generations)
    plt.ylabel("Nombre d'événements de coalescence") # Number of coalescence events.
    plt.savefig("Fig_noCoalescedPairsCohort%s_%s.png" % (str1, str2), bbox_inches="tight")
    plt.show()
    plt.figure(2)
    plt.plot(epochs[:-1], total[1:]-coalesced[1:])
    plt.xlabel("Nombre de générations avant présent (en bins de 100 générations)") # Number of generations before present (in bins of 100 generations)
    plt.ylabel("Proportion de lignées ancestrales restantes") # Proportion of remaining ancestral lineages
    plt.savefig("Fig_propAncestralLineages_Cohort%s_%s_simR_%s.png" % (str1, str2, rep), bbox_inches="tight")
    plt.show()
    return coalesced[1:], total[1:], epochs
```

```
╔══════════════════════════╗
║TreeSequence              ║
╠═══════════════╤══════════╣
║Trees          │   5787202║
╟───────────────┼──────────╢
║Sequence Length│2000000000║
╟───────────────┼──────────╢
║Time Units     │     ticks║
╟───────────────┼──────────╢
║Sample Nodes   │     73042║
╟───────────────┼──────────╢
║Total Size     │ 753.3 MiB║
╚═══════════════╧══════════╝
╔═══════════╤════════╤═════════╤════════════╗
║Table      │Rows    │Size     │Has Metadata║
╠═══════════╪════════╪═════════╪════════════╣
║Edges      │18083512│551.9 MiB│          No║
╟───────────┼────────┼─────────┼────────────╢
║Individuals│   36521│  3.5 MiB│         Yes║
╟───────────┼────────┼─────────┼────────────╢
║Migrations │       0│  8 Bytes│          No║
╟───────────┼────────┼─────────┼────────────╢
║Mutations  │       0│  1.2 KiB│          No║
╟───────────┼────────┼─────────┼────────────╢
║Nodes      │ 2188851│ 59.9 MiB│         Yes║
╟───────────┼────────┼─────────┼────────────╢
║Populations│       3│  2.3 KiB│         Yes║
╟───────────┼────────┼─────────┼────────────╢
║Provenances│       2│ 14.4 KiB│          No║
╟───────────┼────────┼─────────┼────────────╢
║Sites      │       0│ 16 Bytes│          No║
╚═══════════╧════════╧═════════╧════════════╝

Le temps moyen de coalescence, divisé par 4, donne  39747.89704852677
```

```
Nombre de racines distinctes avant récapitation: 1636
Nombre de racines distinctes après récapitation: 1
```

```
<Figure size 432x288 with 0 Axes>
```

Script `POPSIZE_pyslim_Cohort5000_m005.py` **(Part 5/7)**

Simplification will enable us to "clear the brush" from our **tree sequence**. After recapitation, the tree sequence is very dense but above all, we do not need to know the genetic information of all the individuals alive at the present time (which, at this stage, are still integrated into the **tree sequence**, as they are exported by default at the end of step 1). In fact, we are only interested in the genetic information of individuals sampled in *SLiM* between time steps 90 and 100. The simplification process will enable us to retain, in our **tree sequence**, only those genealogies relating to these individuals whose unique identifiers, stored in our `SLiM_samples.tsv` file, can also be found in the **tree sequence**.

The `print(sts)` function allows us to visualize some information about this **tree sequence** after simplification. Compared with the previous version, it contains less "dense" trees (value `Edges` and `Nodes`) and fewer genealogies. Above all, it contains a much smaller number of individuals, 14978 to be precise. This value is very close to the 14856 individuals sampled in step 1. The slight difference is due to the fact that some individuals present in the **tree sequence** were not part of our sample, but were retained (automatically) by *pyslim*, as they were involved in the genealogies of our sample.

In [2]:

```
# -------------------------------------------------------------------------------------------
# SIMPLIFICATION (here, we should specify the ID of the "nodes" associated to individuals memorized 
# during serial sampling in Step 1 thanks to the function "sim.treeSeqRememberIndividuals();" in SLiM.).
# -------------------------------------------------------------------------------------------

# Import of identifiers for all individuals sampled in step 1, stored in the "SLiM_samples.tsv" file. 
# We will use this information to simplify our "tree sequence" following its recapitation.
samples0 = pd.read_csv("SLiM_samples.tsv", delimiter="\t", header=0)
samples = samples0.drop_duplicates(subset='ID', keep='first', inplace=False, ignore_index=True) # We only need unique identifiers, even if some individuals may have been sampled several times.
samples['pid'] = samples.ID.str.split("_").str[2]
samp_ped = samples.pid.astype(int).tolist()

# Creation of an empty list to store the pyslim identifiers of each individual corresponding to the SLiM identifiers (pedigree_id) of the samp_ped samples.
keep_indivs = [] 
for i in rts.individuals():
    pid = i.metadata["pedigree_id"]
    if pid in samp_ped:
        keep_indivs.append(i.id)

# The number of individuals present in samp_ped and in keep_indiv must be identical:
# "Our SLiM_samples.tsv file contains ', len(samp_ped), ' samples with SLiM pedigree_IDs, and our keep_indivs list contains ', len(keep_indivs), ' samples with pyslim individual.id.', sep=''"
print('Notre fichier SLiM_samples.tsv contient ', len(samp_ped), ' échantillons avec un identifiant SLiM pedigree_IDs, et notre liste keep_indivs contient ', len(keep_indivs), ' échantillons avec un identifiant pyslim individual.id.', sep='')

# Create an empty list to store node identifiers (pyslim) associated with keep_indivs samples.
keep_nodes = [] 
for i in keep_indivs:
    keep_nodes.extend(rts.individual(i).nodes)

# WE CAN NOW PROCEED WITH SIMPLIFICATION: 
# keeping only those individuals in our "tree sequence" whose identifiers correspond to the individuals sampled in step 1 (keep_nodes).
# We eliminate the other individuals who were alive at the present time (i.e., in the last generation simulated in SLiM).
sts = rts.simplify(keep_nodes) 
print(sts)
sts.dump("POPSIZE_pyslim_Cohort%s_%s_sts.trees" % (str1, str2))
```

```
/home2/datawork/cdelord/conda-env/simupop-slim/lib/python3.7/site-packages/ipykernel/__main__.py:10: SettingWithCopyWarning: 
A value is trying to be set on a copy of a slice from a DataFrame.
Try using .loc[row_indexer,col_indexer] = value instead

See the caveats in the documentation: https://pandas.pydata.org/pandas-docs/stable/user_guide/indexing.html#returning-a-view-versus-a-copy
```

```
Notre fichier SLiM_samples.tsv contient 14856 échantillons avec un identifiant SLiM pedigree_IDs, et notre liste keep_indivs contient 14856 échantillons avec un identifiant pyslim individual.id.
╔══════════════════════════╗
║TreeSequence              ║
╠═══════════════╤══════════╣
║Trees          │   4827996║
╟───────────────┼──────────╢
║Sequence Length│2000000000║
╟───────────────┼──────────╢
║Time Units     │     ticks║
╟───────────────┼──────────╢
║Sample Nodes   │     29712║
╟───────────────┼──────────╢
║Total Size     │ 694.0 MiB║
╚═══════════════╧══════════╝
╔═══════════╤════════╤═════════╤════════════╗
║Table      │Rows    │Size     │Has Metadata║
╠═══════════╪════════╪═════════╪════════════╣
║Edges      │16636023│507.7 MiB│          No║
╟───────────┼────────┼─────────┼────────────╢
║Individuals│   14978│  1.4 MiB│         Yes║
╟───────────┼────────┼─────────┼────────────╢
║Migrations │       0│  8 Bytes│          No║
╟───────────┼────────┼─────────┼────────────╢
║Mutations  │       0│  1.2 KiB│          No║
╟───────────┼────────┼─────────┼────────────╢
║Nodes      │ 2132052│ 57.9 MiB│         Yes║
╟───────────┼────────┼─────────┼────────────╢
║Populations│       3│  2.3 KiB│         Yes║
╟───────────┼────────┼─────────┼────────────╢
║Provenances│       3│ 14.9 KiB│          No║
╟───────────┼────────┼─────────┼────────────╢
║Sites      │       0│ 16 Bytes│          No║
╚═══════════╧════════╧═════════╧════════════╝
```

Script `POPSIZE_pyslim_Cohort5000_m005.py` **(Part 6/7)**

Now we need to add genetic variability to our **tree sequence**. We can use a classic mutation model (here `msprime.JC69()`, the model of Jukes and Cantor, 1969).

The `print(mts)` function lets us visualize some information about this **tree sequence** after mutations have been added. The number of `Sites` and `Mutations`, always equal to zero in previous versions, have now increased to 5.098e06 and 5.105e06, respectively (some variable sites therefore carry more than one mutation).

In [2]:

```
# -------------------------------------------------------------------------------------------
# ADDITION OF NEUTRAL GENETIC VARIABILITY (OVERLAY/MUTATE)
# (Neutral genetic diversity is distributed, in the form of added variants, along the tree sequence).
# This will enable us to export genotypes for each individual sampled in SLiM. 
# -------------------------------------------------------------------------------------------

mts = pyslim.SlimTreeSequence(msprime.sim_mutations(sts, rate=1.435e-09, model=msprime.JC69(), keep=True))
# Re-scaling of mutation rate per Wright-Fisher generation: 'rate=1.435e-09'.
# "Our tree sequence now includes {mts.num_sites} variable sites, {mts.num_mutations} mutations, and the average nucleotide diversity is {mts.diversity()}.""
print(f"Notre tree sequence comporte maintenant {mts.num_sites} sites variables, {mts.num_mutations} mutations,\n"
      f"et la diversité nucléotidique moyenne est de {mts.diversity():0.3e}.")
print(mts)
mts.dump("POPSIZE_pyslim_Cohort%s_%s_mts.trees" % (str1, str2))
```

```
Notre tree sequence comporte maintenant 5104971 sites variables, 5111560 mutations,
et la diversité nucléotidique moyenne est de 2.221e-04.
╔══════════════════════════╗
║TreeSequence              ║
╠═══════════════╤══════════╣
║Trees          │   4827996║
╟───────────────┼──────────╢
║Sequence Length│2000000000║
╟───────────────┼──────────╢
║Time Units     │     ticks║
╟───────────────┼──────────╢
║Sample Nodes   │     29712║
╟───────────────┼──────────╢
║Total Size     │ 996.0 MiB║
╚═══════════════╧══════════╝
╔═══════════╤════════╤═════════╤════════════╗
║Table      │Rows    │Size     │Has Metadata║
╠═══════════╪════════╪═════════╪════════════╣
║Edges      │16636023│507.7 MiB│          No║
╟───────────┼────────┼─────────┼────────────╢
║Individuals│   14978│  1.4 MiB│         Yes║
╟───────────┼────────┼─────────┼────────────╢
║Migrations │       0│  8 Bytes│          No║
╟───────────┼────────┼─────────┼────────────╢
║Mutations  │ 5111560│180.4 MiB│          No║
╟───────────┼────────┼─────────┼────────────╢
║Nodes      │ 2132052│ 57.9 MiB│         Yes║
╟───────────┼────────┼─────────┼────────────╢
║Populations│       3│  2.3 KiB│         Yes║
╟───────────┼────────┼─────────┼────────────╢
║Provenances│       4│ 15.6 KiB│          No║
╟───────────┼────────┼─────────┼────────────╢
║Sites      │ 5104971│121.7 MiB│          No║
╚═══════════╧════════╧═════════╧════════════╝
```

Script `POPSIZE_pyslim_Cohort5000_m005.py` **(Part 7/7)**

We don't want to export the genetic information from all the variable sites. We'll select a small number of loci whose characteristics we like. For example, we will only export sites that are biallelic, and whose allelic frequencies are high enough to be informative. Here, we export a total of 30,000 loci. Our final genotype table, exported as a .vcf file, will therefore contain the genetic information of the 14856 individuals sampled in step 1 (and whose identifiers are still stored in our `SLiM_samples.tsv` file) at these 30000 loci.

This .vcf file therefore constitutes the main output of our *pyslim* script and will serve as the starting file for any sub-sampling of individuals (e.g. to work only with the genotypes of individuals of a certain age, captured at a certain time step, etc.) or loci (e.g. by sub-selecting 1000 loci from the 30000 available, or by specifically selecting loci from the same chromosome section). This type of post-processing of the .vcf file has been developed using the dedicated R script `POPSIZE_vcf_output_processing.R`, which is based, among other things, on the file `SLiM_samples. tsv` file and uses the *CKMRpop* and *dartR* libraries to manipulate sampled individuals according to different criteria, and generate .vcf sub-files (or other formats, e.g. PLINK) corresponding to sub-selections of individuals/loci.

In [2]:

```
# -------------------------------------------------------------------------------------------
# POST-PROCESSING OF THE TREE-SEQUENCE AND GENOTYPE EXTRACTION.
# Now that we've generated genetic diversity, we can export genotype tables for all or some of the individuals sampled in SLiM:
# -------------------------------------------------------------------------------------------

# Various functions allow us to select variable sites that may be of interest to us.

# Function for removing variable sites with more than 2 alleles (only biallelic SNP loci are kept).
def removeMultiAllelic_global(mts):
    sites_to_discard = []
    for site in mts.sites():
        if len(site.mutations) != 1:
            sites_to_discard.append(site.id)      
    mts_new = mts.delete_sites(sites_to_discard)
    print(mts_new.num_sites, " sites restants après le retrait des loci à plus de 2 allèles.")
    # print(mts_new.num_sites, " sites remaining after removal of loci with more than 2 alleles.")
    return mts_new

# Function for selecting variants according to their minority allele frequency (MAF) on all sampled individuals.
# (Note, however, that this function is not yet optimal, as it considers all samples at all time steps).
# (It should be possible to consider the sampling periods independently of each other, so that we can think carefully about which loci to select).
def removeRareVariants_global(mts, minor_frequency):
    sites_to_discard = []
    for v in mts.variants():
        if np.sum(v.genotypes[mts.samples()]) / len(mts.samples()) < minor_frequency:
            sites_to_discard.append(v.site.id)
    mts_new = mts.delete_sites(sites_to_discard)
    print(mts_new.num_sites, " sites restants après le retrait des loci de MAF < ", minor_frequency, ".")
    # print(mts_new.num_sites, " sites restants after removal of loci with MAF < ", minor_frequency, ".")
    return mts_new

# Function to sample a 'num_loc' number of variants along the genome and then export their genotypes.
def randomSitesSampler(mts, num_loc, span):
    sites_to_discard = []
    if span > chromosomeSize:
        span = chromosomeSize
        print("Warning: la valeur span a été fixée égale à chromosomeSize, car elle excédait initialement cette valeur.")
        # print("Warning: the parameter span was set equal to chromosomeSize, because it previously exceeded this value.")
    for site in mts.sites():
        if site.position > span-1:
            sites_to_discard.append(site.id)        
    mts_new = mts.delete_sites(sites_to_discard)
    sites_to_discard = []
    if num_loc > mts_new.num_sites:
        num_loc =  mts_new.num_sites
        print("Warning: la valeur num_loc value a été fixée égale à mts.num_sites, car elle excédait initialement le nombre de variants disponibles sur la longueur span.")
        # print("Warning: num_loc value has been set equal to mts.num_sites, as it initially exceeded the number of variants available on chromosome section of length 'span'.")
        print("Le nombre de sites variables disponibles est de: ", mts_new.num_sites, ".")
        # print("The number of variable sites available is: ", mts_new.num_sites, ".")
    sample_loc = np.random.choice(mts_new.sites(), size=(mts_new.num_sites-num_loc), replace=False)
    for v in sample_loc:
        sites_to_discard.append(v.id)
    mts_new = mts_new.delete_sites(sites_to_discard)
    print("-- Nombre de variants conservés: ", mts_new.num_sites)
    # print("-- Number or variants remaining: ", mts_new.num_sites)
    return mts_new

chromosomeSize = 2e09 # Size of the simulated genome, 2.0 Gb.
mts1 = removeMultiAllelic_global(mts) # Removal of multi-allelic loci.
mts1 = removeRareVariants_global(mts1, 0.005) # Removal of very low-frequency loci. Use with caution.
mts1 = randomSitesSampler(mts1, 30000, chromosomeSize) # Random sampling of 30000 loci along the genome.
print(mts1)

mts1.dump("POPSIZE_pyslim_Cohort%s_%s_mts1.trees" % (str1, str2))

indivlist = []
indivnames = [] 
for i in mts1.individuals():
    if mts1.node(i.nodes[0]).is_sample():
       indivlist.append(i.id)
       assert mts1.node(i.nodes[1]).is_sample()
       pid0 = i.metadata['pedigree_id']
       indivnames.append(samples.ID[samples.pid == str(pid0)].item())
with open("POPSIZE_pyslim_output_Cohort%s_%s.vcf" % (str1, str2), "w") as vcffile:
    mts1.write_vcf(vcffile, individuals=indivlist, individual_names=indivnames)
    
# End of Python script.
```

```
5098387  sites restants après le retrait des loci à plus de 2 allèles.
2348675  sites restants après le retrait des loci de MAF <  0.005 .
-- Nombre de variants conservés:  30000
╔══════════════════════════╗
║TreeSequence              ║
╠═══════════════╤══════════╣
║Trees          │   4827996║
╟───────────────┼──────────╢
║Sequence Length│2000000000║
╟───────────────┼──────────╢
║Time Units     │     ticks║
╟───────────────┼──────────╢
║Sample Nodes   │     29712║
╟───────────────┼──────────╢
║Total Size     │ 695.7 MiB║
╚═══════════════╧══════════╝
╔═══════════╤════════╤═════════╤════════════╗
║Table      │Rows    │Size     │Has Metadata║
╠═══════════╪════════╪═════════╪════════════╣
║Edges      │16636023│507.7 MiB│          No║
╟───────────┼────────┼─────────┼────────────╢
║Individuals│   14978│  1.4 MiB│         Yes║
╟───────────┼────────┼─────────┼────────────╢
║Migrations │       0│  8 Bytes│          No║
╟───────────┼────────┼─────────┼────────────╢
║Mutations  │   30000│  1.1 MiB│          No║
╟───────────┼────────┼─────────┼────────────╢
║Nodes      │ 2132052│ 57.9 MiB│         Yes║
╟───────────┼────────┼─────────┼────────────╢
║Populations│       3│  2.3 KiB│         Yes║
╟───────────┼────────┼─────────┼────────────╢
║Provenances│       8│ 17.5 KiB│          No║
╟───────────┼────────┼─────────┼────────────╢
║Sites      │   30000│732.4 KiB│          No║
╚═══════════╧════════╧═════════╧════════════╝
```

Our simulation procedure is now complete. To view the data post-processing procedures, please consult the document POPSIZE\_Script\_PostProcessing\_exampleEN.

End of document.
