# Supplementary material for: Effective Population Size Estimation in Large Marine Populations: Considering Current Challenges and Opportunities When Simulating Large Data Sets With High‐Density Genomic Information
Source: Evol Appl. 2025 Jul 28;18(8):e70121. doi: 10.1111/eva.70121 (PMC12304085; doi:10.1111/eva.70121)
Supplement: Supplementary file 3 — Appendix S1.–S4. [file EVA-18-e70121-s001.docx]

**Supplementary information for :**

**Effective population size estimation in large marine populations:**

**Considering current challenges and opportunities when simulating large datasets with high-density genomic information**

**Running title**: *Ne estimation of large populations*

Chrystelle Delord^1^, Sophie Arnaud-Haond^2^, Agostino Leone^,3,4^, Ekaterina Noskova^5,6^, Rémi Tournebize^7^, Patrick Jacques^8^, Francis Marsac^2^, Natacha Nikolic^8,9,10^

1. UMR MARBEC, Montpellier University, IRD, Ifremer, CNRS, La Réunion, France
2. UMR MARBEC, Montpellier University, IRD, Ifremer, CNRS, Sète, France
3. Department of Earth and Marine Sciences (DiSTeM), University of Palermo, Palermo, Italy
4. National Biodiversity Future Center, Palermo, Italy
5. Department of Biology, University, of Fribourg, 1700 Fribourg Switzerland
6. Swiss Institute of Bioinformatics, 1700 Fribourg, Switzerland
7. IRD, UMR DIADE, Montpellier, France
8. INRAE, AQUA, ECOBIOP, France
9. Centre de Recherche sur la Biodiversité et l'Environnement (CRBE), Université de Toulouse, CNRS, IRD, Toulouse INP, Université Toulouse 3 – Paul Sabatier (UT3), Toulouse, France
10. ARBRE – Agence de Recherche pour la Biodiversité à La Réunion, France

**Corresponding author:** [chrys.delord@gmail.com](mailto:chrys.delord@gmail.com)

**Appendix S1** :

**Complementary information about *Ne* estimation methods based on linkage disequilibrium**.

**A. Necessary precautions for estimating *Ne* using linkage disequilibrium-based methods :**

*General principle:*

Given that the relative influence of genetic drift can be distinguished from that of other factors, it is theoretically possible to use the observed linkage disequilibrium in a given sample to infer the *Ne* of the focal population. Weir (1979), Weir and Hill (1980), and Hill (1981) developed a mathematical formalization connecting the observed linkage disequilibrium of a sample to the *Ne* of its population of origin, provided that the assumptions of a Wright-Fisher population (i.e., an idealized population under specific assumptions of constant population size, random mating, nonoverlapping generations and no influence of mutation, selection or migration, Wright 1931) are met and that the genetic distance and recombination rates between pairs of loci are known. This method is based on the assumption that only recombination, genetic drift (related to *Ne*) and systematic sampling bias influence linkage disequilibrium, with other factors having negligible impacts. This method relies on physically linked genetic markers and is therefore more suitable for historical *Ne* estimation. England et al. (2006) and Waples (2006) demonstrated that bias occurs when using Hill's (1981) method with independent genetic markers to obtain contemporary *Ne* estimates. This bias is more significant when the studied sample is small compared with the true *Ne* value, which is a common situation. Thus, Waples (2006) proposed a new *Ne* estimator based on pairs of independent genetic markers, incorporating a systematic correction for sampling bias. This method enables the estimation of contemporary *Ne* and can be adapted to different mating systems, including population random mating and total monogamy. LDNe (Waples and Do 2008) and NeEstimator2 (Do et al. 2014) both implement this estimator and are among the most widely used software programs for estimating contemporary *Ne* for conservation research (Marandel et al. 2019).

Sved et al. (2013), following Sved (1971), proposed a modified linkage-disequilibrium estimator from Weir (1979) that incorporates highly polymorphic markers, such as microsatellites. They derived a correction for systematic sampling bias when haplotypic information is unknown (consistent with Waples 2006) and used genotype permutation to minimize systematic sampling bias as well as other sources of intralocus linkage disequilibrium, such as the presence of null alleles. Hamilton et al. (2018) integrated this estimator into a MATLAB library named SPEEDNe, which includes various options to account for null alleles, rare alleles and missing data. Applying these methods to simulated data, the authors found performance comparable to other methods, although the *Ne* estimates often ranged between those of the estimators of Sved et al. (2013) and of Waples (2006). More specifically, the Sved estimator exhibits lower variability and better approximates to the true *Ne*, although it is slightly negatively biased at low estimated *Ne* (<250) and in presence of a significant proportion of null alleles. Still, direct comparisons between LDNe (Waples and Do 2008) and SPEEDNe (Hamilton et al. 2018) remain rare (but see Lorenzana et al. 2020).

*Overlapping generations and age structure:*

The presence of multiple distinct age cohorts within a population generates a genetic structuring signal that can lead to an overestimation of linkage disequilibrium and an underestimation of *Ne* (Waples et al. 2014). The amount of bias can be difficult to quantify (Luikart et al. 2010) as it closely depends on the life cycle of the species under study and the representativeness of its sampling. In such cases, it is often recommended to sample individuals from a single cohort, as emphasized by Robinson and Moyer (2013) as well as Waples et al. (2014). Another option, potentially more challenging to apply, is to sample a number of cohorts that is at least equivalent to the generation time of the species under study to hope to obtain a reliable estimation of the generational effective size *Ne* (Waples and Do 2010; Waples et al. 2014; Saura et al. 2015). Unfortunately, it is often technically difficult to target specific age classes during a sampling campaign, especially when studying large pelagic populations subject to many logistical constraints. Sometimes, these studies have an opportunistic character, such as sampling from fishing catches. In this case, and if reliable biological information exists on the value of species life traits, it is possible to test the robustness of estimates using simulations. The NEOGEN software, for example (Blower et al. 2019), offers the possibility to visualize the variability of *Ne* estimates using the method developed by Waples (2006) and implemented in the LDNE software (Waples and Do 2008). This visualization is performed based on different sampling strategies, such as the number of individuals collected per age class. It is particularly suitable for the relatively simple case of a population with overlapping generations, for which longevity, age at maturity, age at senescence, number of offspring per reproduction, and mortality per age class (with considered constant fertility) are known. Waples et al. (2014) propose a correction of the linkage disequilibrium-based generational *Ne* estimate based on empirical observations and simulated data of different life history typologies. However, this correction requires knowing the effective size per reproductive cycle (*Nb*), as well as the longevity or age at maturity of the species. It might be advisable to consider this correction with caution as it relies on the assumption of predictability of the relationship between *Nb* and generational *Ne* (Waples et al. 2013). Thus, in the most realistic case of a random and hardly controllable collection of individuals from various age classes and in the absence of applicable correction, it remains complex to avoid underestimating the generational *Ne* of a population with overlapping generations. Marandel et al. (2019), in an application of the method implemented in the LDNE software on simulated data meant to be representative of a thornback ray population (*Raja clavata*, with a generation time of 8.97 years) using the SIMUPOP library (Peng and Amos 2008), observed a systematic underestimation of 31% of the estimated *Ne*, compared to the simulated real Ne. Robinson and Moyer (2013) obtained more optimistic results from simulations of different life history typologies, with generation times ranging from 3 to 14 years, using the SPIP software (Anderson and Dunham 2005), and report an underestimation of around 15%.

*Gene flow and spatial genetic structure:*

Spatial genetic structure, or introgression following immigration of individuals from other populations, are two factors also contributing to linkage disequilibrium. To our knowledge, there is no mathematical formalization that explicitly integrates, in the classical formalization developed by Hill (1981) and Waples (2006), the linkage disequilibrium induced by migration. The presence of genetic substructure within a population, if not taken into account, can lead to an underestimation of the *Ne* of this population via the method implemented in the LDNE software. This underestimation is stronger if gene flow is strongly limited between subgroups (Waples and England 2011; Gilbert and Whitlock 2015; Kopatz et al. 2017). The influence of immigration within a focal population from an unspecified external population is more complex: it could lead to underestimating local *Ne* in the case of weak immigration, but to overestimating *Ne* in the case of strong immigration. Indeed, in this latter case, the estimation no longer represents local *Ne* but tends more or less towards a global *Ne* (meta-*Ne*) integrating the population of interest but also the external populations from which the immigrants originate. Waples and England (2011), however, estimate that the method implemented in the LDNE software provides a good estimation of the local *Ne* of a subpopulation as long as gene flow with the outside does not exceed 5 to 10%, and provided that all considered subpopulations are in migration-drift equilibrium.

The selected genetic markers must also be sufficiently independent to not retain hints of past migration events. Gilbert and Whitlock (2015) present the method implemented in the LDNE software as one of the most robust to the presence of migration for estimating local *Ne*, as long as migration rate per generation remains constant, symmetrical, and do not exceed 1%. Larger gene flows decrease the method's ability to estimate local *Ne*, especially when its true value is high. In such a situation, it becomes extremely complex to determine whether the obtained estimation is closer to a local or global *Ne*. It often represents an intermediate value, difficult to interpret. In some cases, it may be possible to detect immigrant individuals to remove them from the dataset (Waples and England 2011). This is what Macbeth et al. (2013) did to evaluate the local *Ne* of the Indo-Pacific striped tuna off the coast of Australia. The authors show that a marked underestimation of local *Ne* can be caused even by a small number of immigrant individuals. They propose an algorithm for effectively detecting such individuals from distinct populations or even other closely related species in such cases of "pulse" immigration. However, in species where migration flows are assumed to be significant and constant, obtaining the local *Ne* of a subpopulation seems particularly challenging, if not impossible. Furthermore, obtaining the global *Ne* may risk underestimation if even a weak genetic substructure persists within the metapopulation (e.g., Leone et al. 2024; Nikolic et al. 2023). Additionally, it requires sampling covering the entire distribution area of connected subpopulations (Gilbert and Whitlock 2015). In the case of continuously distributed populations, Neel et al. (2013) report good efficiency of the method implemented in the LDNE software provided that the sampling area covers at least the average reproductive distance between individuals. Finally, from a conservation perspective, the work of Ryman et al. (2019) indicates that different types of *Ne* do not always provide the same level of information on population health. They also demonstrate, in a case of an island model with very weak to moderate gene flows between subpopulations, that the obtained estimation remains different from the inbreeding *Ne*, which is often the basis for defining a vulnerable population (e.g., the 50/500 rule, Franklin 1980). Although this aspect is less likely to concern populations of large pelagics, it is useful to remember the diversity of existing *Ne* indices and the specific information they carry to choose the one most likely to address the monitoring and conservation challenges at hand.

*Past demographic and phylogeographic history:*

Past demographic history, particularly fluctuations in *Ne* over time, can influence our ability to estimate contemporary *Ne*. In theory, estimating contemporary *Ne* of a fluctuating population using the method implemented in the LDNE software provides a value tending towards its harmonic mean over several generations preceding sampling. However, in the case of simulated populations with a small *Ne* (300 to 2400 individuals) undergoing a bottleneck to 50 individuals in a single generation, Antao et al. (2011) show the method implemented in the LDNE software to be effective in estimating *Ne* at the present time, at most 2 to 5 generations after the population size drop. This time lag varies depending on numerous parameters, including the intensity of *Ne* variation and its nature (e.g., sudden or gradual). Similarly, for a simulated population with a very small *Ne* (=100), Wang (2016) demonstrates the method's ability to correctly estimate the *Ne* of a population derived from a parental population of different size provided that this difference in population size between parental and descendant generations is significant (on the order of +/- 40%, in the case of this study). Nunziata and Weisrock (2018) obtain similar results in the case of a small *Ne* population (250 to 1000 individuals) undergoing gradual decline, with much more informative precision from 10 generations following the onset of decline (a time step that may represent a very long period in the case of large pelagic species with high generation times).

*The influence of technical artifacts during genotyping:*

Missing data, genotyping errors, and allele dropout are technical artifacts that can influence the quality of *Ne* estimation by linkage disequilibrium method. The presence of null alleles, by reducing the amount of information available in a dataset, can lead to underestimation of linkage disequilibrium intensity and thus overestimation of *Ne* (Akey et al. 2001; Russell and Fewster 2009). Wang (2016) reaches the same conclusions based on simulated microsatellite marker data, as soon as the proportion of null alleles exceeds 5%. Hamilton et al. (2018) justify their slightly different implementation of the $r^{2}$ statistic based on the same observation, compared to that proposed by Hill (1981) and derived by Waples (2006). The authors also advise eliminating any genotype that has uncertainty in the characterization of one of the alleles. However, Nunziata and Weisrock (2018) report a less pronounced effect of null alleles, based on simulated high-density SNP data. Marandel et al. (2020), nonetheless, emphasize the need for careful selection of SNPs for building such datasets, as null alleles and missing data introduce a significant bias into their *Ne* estimates, potentially even greater than that introduced by not considering overlapping generations. Generally, the aforementioned technical artifacts will have a greater impact on loci carrying rare alleles, as they are more likely to suffer from detection failures via current genotyping methods. However, rare alleles have a strong influence on *Ne* estimation. When using the LDNE software, if potential genotyping errors are suspected, it is recommended to eliminate rare alleles from the analysis and thus set the PCRIT parameter (minimum value of allelic frequency integrated into the calculation, any allele with a frequency lower being removed from the dataset) to a value between 1/2S and 1/S (S being the size of the diploid sample). This eliminates from the analysis any singleton or doubleton present in the dataset, which could lead to an artifactual overestimation of *Ne* (Waples and Do 2010). Comparing estimations obtained under different PCRIT values also helps to provide perspective during interpretations (e.g., Macbeth et al. 2013). Using methods based on full genotype likelihoods rather than called genotypes could also help mitigating the influence of genotyping error (Waples 2024).

*The influence of selection and local adaptation phenomena:*

Finally, selection and local adaptation (Hedrick et al. 1978) can also bias *Ne* estimations by acting on linkage disequilibrium as an additional factor to pure genetic drift (Lewontin and Kojima 1960). Especially when using high-density marker datasets, it is essential to verify the neutrality of all loci used during *Ne* estimation by linkage disequilibrium (Waples and Do 2010) through available methods such as BAYESCAN or F-DIST for example.

**B. Calculating confidence intervals :**

LDNE and NeEstimator2 incorporate parametric and non-parametric methods for obtaining confidence intervals around the *Ne* estimate. One of these methods relies on an expected chi-square ($\chi^{2}$) distribution for the ratio between the observed statistic $r^{2}$ and its expectation (Waples 2006). The second method is based on jackknife resampling (without replacement) of compared pairs of loci (Waples and Do 2008). Each method assumes independent comparisons between pairs of loci. However, this assumption loses validity as the number of molecular markers increases because, in reality, each locus is involved in as many comparisons as there are other markers. This leads to a phenomenon of pseudoreplication that generates an artificial increase in precision: confidence intervals are sometimes too narrow, to the extent that they exclude the estimated *Ne* value (Jones et al. 2016; Waples et al. 2016). Consequently, Jones et al. (2016) proposed a new method for calculating confidence intervals, incorporating jackknife resampling of individuals. Their approach is deemed more effective but more conservative and is also sensitive to sample size, as low sample size may lead to overestimate precision, with the lower bound of the confidence interval possibly exceeding the true *Ne* value (Waples et al. 2022). The calculation of confidence intervals around the effective population size estimator by the LDNE software represents a complex task, requiring careful interpretation. Additionally, many *Ne* estimators follow an asymmetric distribution. This implies that when these estimators are applied to multiple distinct samples from the same population to verify their variability, they generate a wider range of higher values than lower values. Consequently, it is often empirically observed that the upper bound of the confidence interval is more difficult to estimate, sometimes exhibiting arbitrarily high or even infinite values, than the lower bound. In such situations, it is sometimes recommended to study the distribution of the inverse of the estimator (1/$\hat{Ne}$) to assess biases and the precision of the estimations (Wang 2001, 2009). The confidence intervals calculated around 1/$\hat{Ne}$ may be based, for example, on a Student's t-distribution (Pudovkin et al. 1996; Luikart & Cornuet 1999). Hamilton et al. (2018) propose other approaches to obtain confidence intervals around the *Ne* estimator, based on resampling individuals or loci via jackknife or permutations, which they compare notably with the method of Jones et al. (2016) mentioned previously. The calculation of these confidence intervals can rely on a normal distribution or simply percentiles. The use of percentiles for calculating confidence intervals seems more appropriate, which aligns with the conclusions of Jones et al. (2016).

**C. Estimating *Ne* using linkage disequilibrium-based methods with recombination information :**

Several studies have attempted to mathematically formalize the relationship between linkage disequilibrium and *Ne* over time (Hayes et al. 2003; Corbin et al. 2012). Barbato et al. (2015) implemented this principle in the SNEP software, allowing for the estimation of *Ne* values at different time steps based on sets of loci characterized by different genetic distances. They present this software as a tool for standardized application of the linkage disequilibrium method to estimate a general trend of *Ne* variation since past generations, thus offering a complementary approach to Waples' method (2006) and Waples and Do's (2008) method for estimating contemporary *Ne*. Barbato et al. (2015) provide an example using real data from a sheep population, for which they estimate *Ne* values ranging from 1500 to a few tens over a duration of one thousand generations. The SNEP software can account for different recombination rates and can be applied to phased or unphased data. However, this type of approach requires a particularly high number of genetic loci (typically several tens of thousands). A too low number of loci may decrease the representativeness of certain classes of linkage distances, thus compromising the quality of *Ne* estimation for the corresponding temporal period, as well as the final estimation of demographic trends.

Hollenbeck et al. (2016) addressed a major limitation by developing an implementation focused on more recent time scales, thus making their method applicable to a more limited number of loci, starting from 1000 biallelic SNPs, accessible via the LINKNE software. Their study, illustrated on real data of the red drum (*Sciaenops ocellatus*), allowed for *Ne* estimates reaching over 4000 over the 20 generations preceding sample collection. This approach facilitates the assessment of more recent trends in *Ne*, although some researchers (Lehnert et al. 2019) recommend a cautious interpretation of the results, suggesting reporting qualitative trends rather than precise values, due to the possible influence of very recent events on the data.

Martinez et al. (2022) attempted to evaluate recent fluctuations in *Ne* in coho salmon (*Oncorhynchus kisutch*) (see Table A1.1). Using the SNEP software, they estimated an effective size of only a few hundred with a slight decline in the most recent generations. However, another method (GONE, Santiago et al. 2020) applied to the same data revealed a much more pronounced decline during the same period, with effective sizes in the range of a hundred only for the 8 to 10 most recent generations. Both the SNEP and LINKNE software correct for systematic sampling bias. Furthermore, the LINKNE software allows for easy calculation of confidence intervals, whereas these must be calculated manually for SNEP. However, like the more traditional methods mentioned earlier, the authors highlight the potential risk of introgression, genetic substructure, or limited sample size for these two approaches.

More recently, Ragsdale and Gravel (2019;2020) proposed the implementation of new linkage disequilibrium-based statistic, computable on distinct putative subpopulations. This approach allows for the first time the integration of genetic substructure information into the estimation of recent and contemporary *Ne*, while also considering systematic sampling bias. It is implemented in the moments.LD library (Jouganous et al. 2017) and allows for the reconstruction of the demographic history of one or more populations. Moreover, this implementation, by ensuring a finer consideration of systematic sampling bias, should limit the extent of bias caused by a small sample size relative to the true *Ne*. Based on a simulated example, the authors suggest relatively similar performance for the estimation of an effective size of 500 with a sample of 10 individuals (2%), and an effective size of 10,000 with a sample of 50 individuals (0.5%). To our knowledge, the three implementations presented (SNEP, LINKNE, and moments.LD) are primarily applied to vulnerable species of small sizes, but examples on pelagic fish are provided in Table A1.1.

Finally, the GONE software (Santiago et al. 2020) focuses on closed populations and therefore does not take into account the potential effects of introgression or genetic substructure. However, the authors introduce a mathematical formalization taking into account, for each time period, the cumulative and residual effects of linkage disequilibrium related to fluctuations in genetic drift over previous periods. The authors also implement a genetic algorithm which allows to assess *Ne* variations with their intensity and duration over a period covering recent generations (up to ~100 generations back). According to the authors, the GONE software seems relatively robust to gene flow within the studied population, genotyping errors, uncertainties in recombination rates, as well as selection effects (Novo et al. 2022) and the presence of overlapping generations (another example is available by Saura et al. (2015) who empirically conclude negligible influence of overlapping generations when using the recombination map-based method for a small population of Iberian pigs). However, like the SNEP software, it requires a large number of genetic markers to ensure sufficient resolution and accurately assess fluctuations in effective size up to 100 generations in the past. Recombination information or at least physical linkage information (i.e., the relative positioning of SNP loci across the genome) is necessary to avoid a significant bias according to a recent work from Gargiulo et al. (2024).

A genetic algorithm was also coupled to the library moments (Jouganous et al. 2017) within the GADMA software by Noskova et al. (2020). We can then wonder about the relative performance of using GADMA with moments, compared to using GONE (both options implementing a genetic algorithm) for the estimation of contemporary and recent effective size in large populations. It is highly likely that the use of the methods presented in this section will complement traditional approaches to effective size estimation from the perspective of large pelagic species, by allowing for the estimation of historical *Ne* and its fluctuations, in addition to contemporary *Ne* (provided that the necessary genomic information is available).

**Table A1.1.** Examples of applications of methods based on linkage disequilibrium analysis for estimating contemporary and/or historical effective population size in pelagic fish or commercially important species, or more generally species with expected high abundance. The columns with gray headers indicate precautions taken, if any, to limit the influence of several confounding factors known to affect bias or precision of estimates. **Abbreviations**: msats: microsatellite markers. L: number of loci. S: sample size. %$\hat{\mathrm{Ne}}$: Proportion represented by sample size relative to estimated effective size. na: "not applicable" (not reported or not relevant). W14: Application of the correction proposed by Waples et al. (2014) to account for age structure using known life history traits. W16: Application of the correction proposed by Waples et al. (2016) to account for effects of physical linkage between markers. INF: estimated value infinite or negative.

| Species | Region | Physical connection | Non-independence | Age structure / Overlapping generations | Introgression / Structure | $\hat{\mathrm{Ne}}$ (CI) | L | S (%$\hat{\mathrm{Ne}}$) | Reference |
| --- | --- | --- | --- | --- | --- | --- | --- | --- | --- |
| **LDNe** : Method of *Waples (2006), Waples et Do (2008)* | | | | | | | | | |
| *Aetobatus narinari* | Florida | na | na | na | No sub-structure detected | 2265.7  (243.3-INF) | 8 msats | 143 (6%) | *Newby et al. (2014)* |
| *Carcharodon carcharias* | Australia | na | na | na | Estimated Ne overall, then separately for 3 identified zones | 1512  (122–INF)  (other estimates unusable) | 6 msats | 97 (6%) | *Blower et al. (2012)* |
| *Carcharodon carcharias* | North Atlantic | na | na | na | Ne estimated separately for 2 identified zones | 32.2  (25.2–42.6)  346.6  (220.2–728.1) | 14 msats | 35 (109%)  131 (38%) | *O’Leary et al. (2014)* |
| *Carcharodon carcharias* | South Africa | na | na | Estimated Ne overall, then separately by groups of individuals classified by size (juveniles vs. adults).  Estimated number in the juvenile group, then correction (W14, eq. 8). | Ne estimated overall, then for a specific aggregation site | 333  (247–487)  351  (233−642) | 14 msats | 233 (70%)  167 (48%) | *Andreotti et al. (2016)* |

**Table A1.1.** (Continued)

| Species | Region | Physical connection | Non-independence | Age structure / Overlapping generations | Introgression / Structure | $\hat{\mathrm{Ne}}$ (CI) | L | S (%$\hat{\mathrm{Ne}}$) | Reference |
| --- | --- | --- | --- | --- | --- | --- | --- | --- | --- |
| **LDNe** : Method of *Waples (2006), Waples et Do (2008)* | | | | | | | | | |
| *Carcharhinus galapagensis* | Galapagos | na | na | na | Ne estimated separately for 2 identified zones | 171 (na - na)  205 (na - na) | 8103 SNPs | 27 (16%)  54 (26%)  Power test performed using NeOGen. | *Pazmiño et al. (2017)* |
| *Carcharias taurus* | Australia | na | na | na | No sub-structure detected | Between  274 (207-398) and  335 (319-352) according to the number of loci | ~2000 SNPs | 57 | *Reid-Anderson et al. (2019)* |
| *Electrona antarctica* | Southern ocean | na | na | na | No sub-structure detected  Ne estimated globally then for each collection site | Between 44 (29-89)  et 1940 (473-INF) per site,  INF (INF-INF) overall. | 7 msats | Between  12 et 79 per site, 400 overall | *Van de Putte et al. (2012)* |
| *Ficedula albicollis* | Baltic sea | r² Computed across all pairs of loci, then corrected (W16, eq. 1a),  vs. r² calculated only between loci on different chromosomes. | na | na | No sub-structure detected | ~38 000  (10 670-INF)  ~23 000  (8430-INF)  after correction W16 (eq. 1a) | 78 636 SNPs | 85 (45 et 40 on two periodss) | *Nadachowska-Brzyska et al. (2021)* |

**Table A1.1.** (Continued)

| Species | Region | Physical connection | Non-independence | Age structure / Overlapping generations | Introgression / Structure | $\hat{\mathrm{Ne}}$ (CI) | L | S (%$\hat{\mathrm{Ne}}$) | Reference |
| --- | --- | --- | --- | --- | --- | --- | --- | --- | --- |
| **LDNe** : Method of *Waples (2006), Waples et Do (2008)* | | | | | | | | | |
| *Prionace glauca* | Pacific North | na | na | na | No sub-structure detected. | 5468  (2802-52,352) | 14 msats | 844 (15%) | *King et al. (2015)* |
| *Scombero-morus commerson* | Australia | na | na | na | Elimination of immigrant or hybrid individuals. | - 40 163 (= INF)  (19 595-INF) | 7 msats | 5413  Power test conducted using SHAZA | *Macbeth et al. (2013)* |
| *Sphyrna lewini* | Pacific (Mexico) | na | na | Capture of yearlings exclusively | Ne estimated for each collection site and by region. | Between 34 (14-INF) and INF (INF-INF) | 11 msats | Between 20 and 63  per site. | *Rangel-Morales et al. (2022)* |
| *Sphyrna zygaena* | South Africa | na | na | na | Ne estimated globally, then separately for 3 identified zones. | 6783 (INF-INF) overall  INF (INF-INF) per region | 7 msats | Between  10 and 48 per region | *Kuguru et al. (2019)* |
| *Stegostoma fasciatum* | Australia | na | na | na | Population considered homogeneous. | 377  (274–584) | 13 msats | 114 (30%) | *Dudgeon & Ovenden (2015)* |
| *Stereolepis gigas* | Pacific North | na | na | na | No sub-structure detected.  Ne estimated globally and then for each collection site. | Between 68 (16-INF) and INF (21-INF) per site,  153 (84–539) overall | 12 msats | Between  9 and 21 per site, 61 overall (40%) | *Chabot et al. (2015)* |

**Table A1.1.** (Continued)

| Species | Region | Physical connection | Non-independence | Age structure / Overlapping generations | Introgression / Structure | $\hat{\mathrm{Ne}}$ (CI) | L | S (%$\hat{\mathrm{Ne}}$) | Reference |
| --- | --- | --- | --- | --- | --- | --- | --- | --- | --- |
| **LDNe** : Method of *Waples (2006), Waples et Do (2008)* | | | | | | | | | |
| *Thunnus maccoyii* | Indian Ocean | Correction (W16, eq. 1a) | na | Ne estimated over all adult cohorts, then corrected  based on the AL/Gen ratio  (W14, Table S1).  Nb estimated over 1 juvenile cohort, then corrected (W14, eq. 8). | na | Variable number and effective population size:  10,000-100,000,  possibly infinite.  (>10,000-INF) | 27 msats | Approximately 1000 adults and 1000 juveniles per year (1-10%), over 5 consecutive years (replicates). | *Waples et al. (2018)* |
| *Thunnus thynnus* | North Atlantic and Mediterranean | na | na | Capture of yearlings exclusively | Ne estimated separately for 2 identified zones. | 3437 (470-INF)  4682 (1841-INF) | 95 SNPs | 80 (2%)  413 (9%) | *Puncher et al. (2018)* |
| **SNeP** : Method of *Barbato et al. (2015)* | | | | | | | | | |
| *Hippoglossus hippoglossus* | North Atlantic | GR available for the species | na | na | Ne estimated separately for 2 identified zones. | Linear decline of 50% over the last 7000 years | >29 000 SNPs | 734 | *Kess et al. (2021)* |
| *Oncorhynchus kisutch* | South America (Chile) | GR available for the species | na | na | The population is considered homogeneous. | Linear decline from 543 to 10 over the last 241 generations | >93 000 SNPs | 64 | *Barria et al. (2019)* |

**Table A1.1.** (Continued)

| Species | Region | Physical connection | Non-independence | Age structure / Overlapping generations | Introgression / Structure | $\hat{\mathrm{Ne}}$ (CI) | L | S (%$\hat{\mathrm{Ne}}$) | Reference |
| --- | --- | --- | --- | --- | --- | --- | --- | --- | --- |
| **SNeP** : Method of *Barbato et al. (2015)* | | | | | | | | | |
| *Oncorhynchus kisutch* | Japan | GR available for the species | na | na | Ne estimated separately for each site | Gradual decline from ~100 or ~400 to <50 over the last 20 generations | 51 794 à  >135 000 SNPs | Between  22 and 112 per site | *Martinez et al. (2022)* |
| *Cyanistes caeruleus* | Chile, North America | GR available for *Parus major* | na | na | Ne estimated separately for each collection site | Linear decline from ~1000-2500 to <500 over the last 100 generations | >144 000 SNPs | 947 | *Perrier et al. (2020)* |
| *Lynx lynx* | France, Corsica | GR available for the species | na | na | Ne estimated separately for each region | Linear decline from ~2500 to <500 over the last 2000 years | Whole genome data | 80 | *Lucena-Perez et al. (2020)* |

**Table A1.1.** (Continued)

| Species | Region | Physical connection | Non-independence | Age structure / Overlapping generations | Introgression / Structure | $\hat{\mathrm{Ne}}$ (CI) | L | S (%$\hat{\mathrm{Ne}}$) | Reference |
| --- | --- | --- | --- | --- | --- | --- | --- | --- | --- |
| **LINKNe** : Method of *Hollenbeck et al. (2016)* | | | | | | | | | |
| *Gadus morhua* | North Atlantic | GR available for the species | na | na | Population considered homogeneous  Ne estimated separately for 3 ecotypes. | Linear decline of ~50% over 30 years  Recent estimates between 175 and 9697. | 6270 SNPs | 330 | *Kess et al. (2019)* |
| *Salmo salar* | North Atlantic | GR available for the species | na | na | Exclusion of individuals from domestic populations  Ne estimated separately for each site  Method assumed to be robust to natural sub-structuring | Stable Ne or linear decline over 40 years  Recent estimates between 10^2^ and 10^4^ | 1278 SNPs | 4493 | *Lehnert et al. (2019)* |
| *Tachypleus gigas* | Strait of Singapore | GR available for the species | na | na | Population considered homogeneous | Linear decline from ~15000 to <5000 over the past 5000 years | >108 000 SNPs | 116 | *Tang et al. (2021)* |

**Table A1.1.** (Continued)

| Species | Region | Physical connection | Non-independence | Age structure / Overlapping generations | Introgression / Structure | $\hat{\mathrm{Ne}}$ (CI) | L | S (%$\hat{\mathrm{Ne}}$) | Reference |
| --- | --- | --- | --- | --- | --- | --- | --- | --- | --- |
| **Moments.LD** : Method of *Ragsdale et al. (2020)* | | | | | | | | | |
| *Esox lucius* | China | GR available for the species | na | na | Ne estimated separately for each site | Contemporary Ne ranging from 68 (na – na) and 1102 (na - na) per site | 14 124 SNPs | Between  22 and 42 per site | *Luan et al. (2021)* |
| **GONE** : Method of *Santiago et al. (2020)* | | | | | | | | | |
| *Ficedula albicollis* | Baltic sea | GR available for the species | na | na | Pas de sous-structure détectée. | Progressive decline from ~10000 to <5000 over the last 100 generations | ≥78 636 SNPs | 85 (45 and 40 on two periods) | *Nadachowska-Brzyska et al. (2021)* |
| *Oncorhynchus kisutch* | Japan,  Chile, North America | GR available for the species | na | na | Ne estimé séparément pour chaque site | Progressive decline from ~5000 to <150 over the last 20 generations | 51 794 à  >135 000 SNPs | Entre  22 et 112 par site | *Martinez et al. (2022)* |

**Appendix S2.**

**Complementary information about *Ne* estimation methods based on the site frequency spectrum**.

SFS-based methods for short and long-term *Ne* estimation could derive the theoretical SFS using diffusion equations (e.g., Gutenkunst et al. 2009; Portik et al. 2017; Jouganous et al. 2017; Gutenkunst 2021; Huang et al. 2023), the continuous-time Moran model (e.g., Durrett 2008; Kamm et al. 2017; 2020) or coalescent-based models (e.g., Excoffier et al. 2013, 2021). Diffusion equations are the basis of widely used inference tools integrating multiple populations such as the software δaδi (Gutenkunst et al. 2009) and its more recent advancements (e.g. dadi-pipeline, Portik et al. 2017; dadi.CUDA, Gutenkunst 2021; dadi-cli, Huang et al. 2023). These, along with some other approaches (e.g., the software *moments*, that uses a linear system of ordinary linear differential equations, Jouganous et al. 2017) allow for an exact and precise calculation of the theoretical SFS but are mathematically complex to manipulate. Their implementation is therefore limited to relatively simple demographic models, mostly limited to three (max 5) populations.

Alternative models such as the continuous-time Moran model (Durrett 2008) or coalescent frameworks (Kingman 1982) provides more flexibility. The former has been used to formalize the expected SFS in the case of overlapping generations and multiple populations (up to 10), initially isolated (Kamm et al. 2017) or accounting for punctual gene flow as in the momi2 library (Kamm et al. 2020). The coalescent model relies on the genealogical relationships between the different copies of a gene in a sample up to their most recent common ancestor (MRCA), retracing across generations the demographic parameters that have affected the population over time. Correct approximation of the Wright-Fisher model can be obtained under certain conditions (Hudson 1983; Tajima 1983), and its flexibility allows simulations of populations integrating complex demographic parameters such as fluctuations in *Ne* over time (Adam and Hudson 2004; Nikolic and Chevalet 2014), gene flow between multiple distinct populations (Takahata 1988; Keinan et al. 2007; Gutenkunst et al. 2009; Excoffier and Foll 2011), recombination (Hudson 1983; Wiuf and Hein 1999; McVean and Cardin 2005), or selection (Hudson and Kaplan 1988). Some coalescent-based models can also account for specific mutation modalities as in the case of microsatellites (Excoffier and Foll 2011; Nikolic and Chevalet 2014), or even for a strong variability in reproductive success that can lead to multiple coalescence for the same generation (Eldon and Wakeley 2006; Tellier and Lemaire 2014; Montano 2016). A detailed history of the coalescent model and its successive developments has been published by Wakeley (2020).

The coalescent model is the basis of many phylogeography or demographic inference software (Bourgeois and Warren 2021). The most used coalescent-based tools fastSimcoal2 (Excoffier et al. 2013, 2021), allow testing of diverse scenarios and parameter estimation but may lack precision for small *Ne* or very recent events due to biases, particularly underestimating rare alleles (singletons). For example, its application to vulnerable populations of very small effective size must be done with caution since it then departs from the assumptions of a classic Wright-Fisher population (Montano 2016; Lauterbur 2019). Advanced models bypass the need to *a priori* define demographic scenarios but are then restricted to very specific cases. For instance, skyline plot tools (Drummond et al. 2006) and Stairway Plot (Liu and Fu 2015) allow the evaluation of effective size fluctuations over time within a homogeneous population, yet skyline plots are restricted to non-recombining markers (e.g. mitochondrial DNA) and Staiway Plot requires whole-genome sequencing data. Both methods may also suffer from unaccounted genetic structure (see Heller et al. 2013 for skyline plot).

Some tools integrate linkage disequilibrium information in addition to the SFS, such as PopSizeABC (Boitard et al. 2016), again focusing on a single population and its effective size fluctuations without considering possible effects of genetic structure and gene flow (but see also ABLE, Beeravolu et al. 2018). They allow exploring a broader parameter space and are robust to some constraints related to the selection of molecular markers used for the construction of the SFS. They can thus, for example, be used for exploratory purposes before the application of more complex methods and demographic models (Liu and Fu 2015; Salmona et al. 2017).

**Appendix S3.**

**Complementary information about the simulation framework.**

Our simulation procedure has been summarized in Figure 2 from main text, with detailed information provided here and on GitHub ([https://github.com/[author]/POPSIZE-Project-SLiM_Scripts](https://github.com/%5bauthor%5d/POPSIZE-Project-SLiM_Scripts)).

Initially, an individual-centered approach allows simulating a simple yet biologically realistic scenario using the SLiM software (Haller and Messer 2018a,b). Two subpopulations, exchanging gene flow, are structured by age with overlapping generations. Individuals may differ in their fertility and survival rates, which are identical within and between subpopulations and sexes but vary with age. This simulation spans 100 time-steps, corresponding to 100 reproductive cycles. At any point, a subset of individuals can be sampled from this simulation, such as different cohorts at different time steps.

Following this initial step, information about the sampled individuals can be exported, including their pedigree (e.g., to identify related individuals), age, or location (subpopulation) at sampling, as well as genetic information in the form of genotypes or genealogies (e.g., via tree sequence recording), if genome and genetic variation simulation were conducted in parallel. While the individual-centered approach offers biological realism, it requires significant computational time and memory, especially for simulating large populations like those of large pelagic species and genomes of substantial size (Kelleher et al. 2018). Thus, it is challenging to deploy over a sufficient number of generations for effective burn-in or to incorporate the past evolutionary trajectory of simulated samples.

Thus, it is common for a population past evolutionary trajectories or the characterization of its neutral genetic variability to be developed in parallel using coalescent-based simulations. Unlike individual-centered approaches, coalescent-based simulations do not simulate each individual, gene, and generation separately but trace genealogies within a sample or population, leading to a significant decrease in simulation duration. While simulation algorithms based on coalescent theory may be less reliable for recent time scales and large sample and genome sizes compared to individual-centered approaches or the exact Wright-Fisher model, they generally provide sufficient approximation for broader time scales and reconstructing past events. The combination of individual-centered and coalescent-based simulations is often termed a hybrid approach, sometimes referred to as a "sideways" approach. Simulators such as msms (Ewing and Hermisson 2010), discoal (Kern and Schrider 2016), or quantiNemo2 (Neuenschwander et al. 2018) exemplify hybrid simulation methods. Complementary tools like SIMUPOP (Peng and Amos 2008) and fastSimcoal2 (Excoffier et al. 2013, 2021) can also be combined. For instance, Waples et al. (2016) used this approach to generate data for evaluating the influence of physical linkage between loci on a linkage disequilibrium-based effective population size estimator. However, combining such tools can be laborious, requiring a seamless transition between the individual-centered and coalescent phases, especially when the former relies on a biologically realistic framework where the assumptions of a Wright-Fisher population, fundamental to the coalescent's approximation, do not hold (Kelleher et al. 2018).

Software like SLiM and pyslim/msprime offer features, including tree sequence recording, facilitating the integration of these two phases. In our simulation procedure, demographic and genealogical data of the sampled individuals from the first step (individual-centered simulation using SLiM) initialize a coalescent-based simulation step (pyslim/msprime). This step reconstructs historical evolutionary trajectories for simulated populations and assigns neutral genetic variability to them. At the end of the second step, additional information about the sampled individuals, such as their genotypes at a chosen number of variable genetic markers, can be exported. The SLiM and pyslim/msprime programming scripts developed for this work are accessible via the GitHub link.

- **Principle of tree-sequence recording**

The concept of tree sequence recording was first introduced by Kelleher et al. (2016) as an efficient method to simulate recombinant chromosomal segments compared to traditional coalescent approaches. A tree sequence file optimally encodes genealogies of a sample along the genome, incorporating both coalescence and recombination events. This optimized encoding describes genealogies of genes within a sample, offering a more suitable description of recombination processes and enhancing simulation speed, enabling larger sample sizes than classical approaches. Tree sequence recording was initially implemented in the msprime software.

It was later applied to individual-centered simulations to encode the pedigrees of simulated individuals (Kelleher et al. 2018), facilitating the reconstruction of complete individual genealogies throughout the genome. This process avoids the simulator's need to store genetic variability information for all individuals continuously. Recording genealogies as tree sequences preserves only the necessary information for subsequent genetic variation addition using much faster coalescent-based algorithms. This allows benefiting from the advantages of individual-centered approaches, notably precision in simulating biologically realistic populations and large samples, while reducing computational requirements. Haller et al. (2018) implemented this tree sequence recording functionality in the SLiM software, highlighting its usefulness in combining individual-centered approaches to simulate populations departing from ideal Wright-Fisher assumptions with coalescent approaches to reconstruct their past evolutionary trajectories (a process termed "recapitation") and assign genetic variability.

- **Simulation of two age-structured sub-populations using individual-based simulations**

**Table A3.1.** Vital parameter rates (mortality and fecundity) per age class in each subpopulation. These parameters are identical across each simulated subpopulation and are also identical between male and female individuals (with a balanced sex ratio).

| Age Class | 0 | 1 | 2 | 3 | 4 | 5 | 6 | 7 | 8 | 9 | 10 | 11 | 12 | 13 | 14 | 15 |
| --- | --- | --- | --- | --- | --- | --- | --- | --- | --- | --- | --- | --- | --- | --- | --- | --- |
| Mortality rate | 0,00 | 0,46 | 0,38 | 0,34 | 0,31 | 0,29 | 0,31 | 0,34 | 0,38 | 0,44 | 0,55 | 0,55 | 0,55 | 0,55 | 0,60 | 1,00 |
| Relative fecundity rate | 0,00 | 0,00 | 0,00 | 0,00 | 0,10 | 0,20 | 0,30 | 0,40 | 0,50 | 0,60 | 0,70 | 0,80 | 0,90 | 1,00 | 1,10 | 1,20 |
| Number of individuals | 5000 | 5000 | 2700 | 1674 | 1104 | 762 | 542 | 374 | 246 | 152 | 86 | 38 | 18 | 8 | 4 | 2 |

**Table A3.2.** Summary of key parameters in the simulation procedure performed using SLiM 3.7.

| Parameters | Fixed value | Definition |
| --- | --- | --- |
| K | 17710 | Total abundance of individuals (males and females combined, with balanced sex ratio) in each of the simulated sub-populations using the SLiM software. |
| final_cohort_size | 5000 | Number of newborns generated at each reproductive cycle. |
| L | Cf. Table A3.1 | Mortality rate for each age class, identical between males and females and identical between sub-populations. |
| B | Cf. Table A3.1 | Fertility rate for each age class, identical between males and females and identical between sub-populations. |
| W | Cf. Table A3.1 | Total population size, in number of individuals (males and females combined with balanced sex ratio), in each age class. |
| m | 0,05 | Migration rate between sub-populations. It is constant and symmetrical from one time step to another. |
| Genome size | 2x10^9^ pb | Size of the simulated genome for each individual. |
| Recombination rate | 1x10^-8^ | Probability of recombination per base pair of the genome and per reproductive cycle. |
| Theoretical effective size | 3314 | Theoretical demographic effective size, per sub-population and per generation, calculated using the AGENE software (Waples et al. 2011). |
| Observed effective size | 2772 | Observed demographic effective size, per sub-population and per generation, using the formula of Hill (1972) and Crow and Kimura (1970) applied to several successive cohorts (see section I.b and GitHub). |

- **Reconstructing ancestry and population genetic diversity using a coalescent-based approach**

For each individual, SLiM simulates genetic information in the form of a diploid genome, with a size fixed at 2Gb, a realistic value for a vertebrate species. This genome is subdivided into 5 recombinant segments to mimic the behavior of 5 independent "chromosomes." This number, although less realistic (as the number of chromosomes is higher in species like the albacore tuna and sharks, Stanhope et al. 2023; Uchino et al. 2016), was chosen due to computational time constraints (simulation duration increasing exponentially with the number of independent genome sections to be simulated). Within each segment, the recombination rate was set at 1x10-8 per base pair per reproductive cycle. However, at this stage of the simulation, no genetic diversity is simulated. This will be generated in the phase using the *pyslim* and *msprime* libraries.

At the end of the simulation phase conducted via the SLiM software, we have a tree sequence file containing genealogical information throughout the genome of sampled individuals between time steps 90 and 100. This serves as our starting point for the beginning of the simulation phase conducted via the *pyslim* and *msprime* libraries. The objective of this second step is, on one hand, to reconstruct the evolutionary trajectory (ancestral patterns) of the two simulated sub-populations using the SLiM software (i.e., their history prior to the last 100 time steps), and on the other hand, to attribute neutral genetic variation (distribution of genetic mutations generating different alleles along the genome) to the simulated individuals, based on the reconstructed demographic history and the topology of the tree sequence. These two processes occur in two distinct steps, recapitation, and mutation distribution ("mutation overlay"). During the recapitation phase, we simulated a maximally simplified evolutionary trajectory, consisting simply of merging the two sub-populations into a single ancestral population (with an effective size equivalent to the sum of the sizes of the two sub-populations). The recapitation process ensures the completion of the "burn-in" phase associated with the parameters set for the entire simulation, including both SLiM and pyslim/msprime steps. To ensure the consistency of the time scales considered, respectively, by the individual-centered approach of the SLiM software outside the framework of a Wright-Fisher population (where each time step represents a reproductive cycle rather than a complete generation), and by the coalescence approach of the pyslim and msprime libraries (where each time step represents a Wright-Fisher generation), it is necessary to rescale some of the demographic parameters such as effective size or recombination and mutation rates. The calculation of the generation time, which we perform during the SLiM simulation phase using a dedicated function, allows for this adjustment. During the genetic mutation distribution phase, we used the classic Jukes and Cantor mutation model (1969).

**Table A3.3.** Fixed parameters set in the *pyslim/msprime* simulation phase.

| Parameters | Fixed value | Definition |
| --- | --- | --- |
| gen_time | 6,989 | Generation time calculated based on observed demographic parameters within the populations simulated via the SLiM software. |
| ne_demo | 2772 | Demographic effective size per subpopulation, calculated based on observed demographic parameters within the populations simulated via the SLiM software and used as a proxy for coalescent effective size to initiate the simulation phase using the coalescent. |
| ne_pyslim | ne_demo x gen_time  = 19373 | Coalescent effective size per subpopulation, rescaled to ensure the transition between SLiM -> pyslim/msprime time scales. |
| Effective size of the ancestral population | 2 x ne_pyslim  = 38475 | Effective size of the ancestral population, considered equal to the sum of the effective sizes of the subpopulations which are of constant size and exchange homogeneous gene flow. |
| Divergence time in generations | 4 x ne_pyslim  = 77492 | Divergence time (in number of Wright-Fisher generations), the duration since the subdivision of the ancestral population into two subpopulations exchanging gene flow. |
| m | 0,05 | Migration rate (gene flow) between subpopulations. It is symmetrical and constant from one generation to the next. |
| Genome size | 2x10^9^ pb | Genome size simulated for each individual. |
| Recombination rate | 1x10^-8^/gen_time  = 1,435x10^-9^ | Recombination probability per base pair of the genome and per Wright-Fisher generation, rescaled to ensure the transition between SLiM -> pyslim/msprime time scales. |
| Mutation rate | 1x10^-8^/gen_time  = 1,435x10^-9^ | Mutation rate per base pair of the genome and per Wright-Fisher generation, rescaled to ensure the transition between SLiM -> pyslim/msprime time scales. |
| Number of sampled loci | 30000 | Number of loci randomly selected along the entire genome (i.e., 5 "chromosomal" sections) at the end of the simulation. |

At the end of these two processes, we can extract the genotypic information of all simulated individuals via the SLiM software, in addition to their genealogical, pedigree, and demographic information such as age or population of origin. The total number of individuals available in the present case, at the end of the simulation, is 14856. These individuals originate from each of the two subpopulations in equivalent proportions and from each age class 1 to 15 in representative proportions of the age structure. Moreover, as previously indicated, they come from staggered sampling between time steps 90 to 100, in equivalent proportions. For each individual, we extracted genotypic information on 30000 homologous SNP loci. The genotypic and demographic information of the sampled individuals will undergo post-processing described in the following section and on the GitHub link.

- **Post-processing of simulated data**

At the end of the simulation procedure described above, we obtain a variant call format (VCF) file containing genotypes for 30000 loci of all (without distinction) the 14586 simulated individuals, each with a unique identifier. Individual-specific data, on the other hand, are stored in accompanying text files, three of which are of particular interest at this stage:

- "SLiM_samples.tsv" file: For each sampled individual, it contains information about their subpopulation, sampling time step, and indirect information such as their age at sampling. For example, individual F89_1_908132, a female born at time step 89 in subpopulation 1 with unique identifier 908132, was captured at time step 90 in subpopulation 1 (indicating she was then 1 year old). Individual M92_2_944478, a male born at time step 92 in subpopulation 2 with unique identifier 944478, was captured at time step 96 in subpopulation 1 (indicating he was then 4 years old).
- "SLiM_demo_table.tsv" file: It contains calculations of generation time and demographic effective size per generation, among others, performed for several successive cohorts during the simulation via the SLiM software. It allows us to retain the average effective size observed in the simulated data, which we expect to retrieve by applying indirect estimation methods based on the genetic information of these individuals.
- "SLiM_ancestries.tsv" file: For each sampled individual, it contains the unique identifiers of their two parents and four grandparents. This file will allow deduction and analysis of different kinship relationships between individuals, using the R library CKMRpop (Anderson 2022).

With the unique identifiers of individuals, it's possible to cross-reference the sampling information from "SLiM_samples.tsv" with the genotypic information contained in the global .vcf file, which serves as our reference base. It's more optimal to have a global genotype file from which we can extract information for a small number of individuals of interest, whose unique identifiers are known (for example, all individuals sampled at time step 100), than to export multiple distinct genotype files at the end of the simulation for each combination of individuals of interest (risking many redundancies between files and forgetting to export certain interesting combinations of individuals).

The R scripts available via GitHub provide an example of sub-selection of individuals within the global genotype file. From this reference file, we generate 12 subsets of genotypic data, each containing more or fewer individuals (14, 50, 55, or 139 individuals in our example) collected at time step 100, and each containing more or fewer loci (1000, 10000, or 30000 loci in our example). Each of these datasets is converted into the necessary formats for reading by the software NEESTIMATOR2 (Do et al. 2014), GONE (Santiago et al. 2020), and GADMA (Noskova et al. 2020) for the implementation of the method of moments (Jouganous et al. 2017). The GitHub link also provides an example of using simulated data in the context of a CKMR method approach.

**Table A3.4.** Characteristics of the 108 genotypic data subsets derived from 9 independent simulations conducted with migration flows '*m*' between subpopulations set at 0.01, 0.05, or 0.10, and repeated 3 times (replicates) for each value of '*m*'. The 3 replicates for a subset of data with the same parameters are grouped into a single line.

| Abundance | Target *Ne* | Gene flow | Number of réplicates | Average number of  sampled individuals per subpopulation | Number of loci |
| --- | --- | --- | --- | --- | --- |
| 17710 | 2772 | 0,01 | 3 | 14 | 30000 |
| 17710 | 2772 | 0,01 | 3 | 50 | 30000 |
| 17710 | 2772 | 0,01 | 3 | 56 | 30000 |
| 17710 | 2772 | 0,01 | 3 | 140 | 30000 |
| 17710 | 2772 | 0,01 | 3 | 14 | 10000 |
| 17710 | 2772 | 0,01 | 3 | 50 | 10000 |
| 17710 | 2772 | 0,01 | 3 | 56 | 10000 |
| 17710 | 2772 | 0,01 | 3 | 140 | 10000 |
| 17710 | 2772 | 0,01 | 3 | 14 | 1000 |
| 17710 | 2772 | 0,01 | 3 | 50 | 1000 |
| 17710 | 2772 | 0,01 | 3 | 56 | 1000 |
| 17710 | 2772 | 0,01 | 3 | 140 | 1000 |
| 17710 | 2772 | 0,05 | 3 | 14 | 30000 |
| 17710 | 2772 | 0,05 | 3 | 50 | 30000 |
| 17710 | 2772 | 0,05 | 3 | 56 | 30000 |
| 17710 | 2772 | 0,05 | 3 | 140 | 30000 |
| 17710 | 2772 | 0,05 | 3 | 14 | 10000 |
| 17710 | 2772 | 0,05 | 3 | 50 | 10000 |
| 17710 | 2772 | 0,05 | 3 | 56 | 10000 |
| 17710 | 2772 | 0,05 | 3 | 140 | 10000 |
| 17710 | 2772 | 0,05 | 3 | 14 | 1000 |
| 17710 | 2772 | 0,05 | 3 | 50 | 1000 |
| 17710 | 2772 | 0,05 | 3 | 56 | 1000 |
| 17710 | 2772 | 0,05 | 3 | 140 | 1000 |
| 17710 | 2772 | 0,10 | 3 | 14 | 30000 |
| 17710 | 2772 | 0,10 | 3 | 50 | 30000 |
| 17710 | 2772 | 0,10 | 3 | 56 | 30000 |
| 17710 | 2772 | 0,10 | 3 | 140 | 30000 |
| 17710 | 2772 | 0,10 | 3 | 14 | 10000 |
| 17710 | 2772 | 0,10 | 3 | 50 | 10000 |
| 17710 | 2772 | 0,10 | 3 | 56 | 10000 |
| 17710 | 2772 | 0,10 | 3 | 140 | 10000 |
| 17710 | 2772 | 0,10 | 3 | 14 | 1000 |
| 17710 | 2772 | 0,10 | 3 | 50 | 1000 |
| 17710 | 2772 | 0,10 | 3 | 56 | 1000 |
| 17710 | 2772 | 0,10 | 3 | 140 | 1000 |

**Appendix S4.** Configuration of NEESTIMATOR 2, GONE and GADMA software for application to the simulated data:

**NEESTIMATOR 2**: The simulated data were converted to genepop format as described in the document ‘POPSIZE_Script_PostProcessing_exampleEN’. They were analyzed in a standardized way with NEESTIMATOR 2 using the linkage disequilibrium method, retaining only loci with MAF ≥ 0.05 (PCRIT parameter = 0.05). Effective size estimates were performed separately for each subpopulation and each simulation replicate.

**GONE**: The simulated data were converted to PLINK format (.ped and .map extensions) as described in the document ‘POPSIZE_Script_PostProcessing_exampleEN’. They were analyzed in a standardized way with GONE using the parameters recommended by the authors in the user manual (capture of the 'INPUT_PARAMETERS_FILE' configuration file below). Effective size estimates were performed separately for each subpopulation and each simulation replicate.

#INPUT_PARAMETERS_FILE:

########################################################

PHASE=2 # Phase = 0 (pseudohaploids), 1 (known phase), 2 (unknown phase)

cMMb=1 # centimorgans per megabase (if distance is not available in map file).

DIST=1 # none (0), Haldane correction (1) or Kosambi correction (2)

NGEN=500 # Number of generations for which linkage data is obtained in bins

NBIN=250 # Number of bins (e.g. if 400, each bin includes NGEN/NBIN = 2000/400 = 5 generations)

MAF=0.0 # Minor allele frequency (0-1) (recommended 0)

ZERO=1 # 0: Remove SNPs with zeroes (1: allow for them)

maxNCHROM=-99 # Maximum number of chromosomes to be analysed (-99 = all chromosomes)

maxNSNP=50000 # Maximum approx number of SNPs per chromosomes to be analysed

hc=0.05 # Maximum value of c analysed (recommended 0.05; maximum is 0.5)

REPS=40 # Number of replicates to run GONE (recommended 40)

threads=-99 # Number of threads (if -99 it uses all possible processors)

###################################################################

**GADMA (moments)**: The simulated data were converted to *variant calling format* (extension .vcf) as described in the document ‘POPSIZE_Script_PostProcessing_exampleEN’. They were analyzed in a standardized way with GADMA using the "structure" type model [1;1] (as opposed to the "custom" model, Noskova et al. 2020) (capture of the 'params_file' configuration file below). RMS size estimates were performed separately for each subpopulation and each simulation replicate, with 32 independent runs for each estimate. The generation time was set at 6.98 and the values of the Theta0 parameter were calculated, for each subset of data, using the formula 4 ×simulated mutation rate ×L (see GADMA documentation) with:

$$L=\frac{\left( Number of exported loci \times Number of simulated basepairs \right)}{Number of variable sites obtained by simulation}$$

This $L$ value could vary slightly from one simulation to another (e.g., between replicates). In the example presented here, the phase of assigning neutral genetic variability to the simulated genomes generated 5,104,971 variable sites out of the 2,000,000,000 base pairs simulated. We exported 30,000 loci. We therefore calculate:

$$L=\frac{30 000 \times2 000 000 000}{5 104 971}\approx11 753 250$$

Then *Theta0* = $4 \times1e-08 \times11 753 250\approx0,47$.

A typical example of param_file used to launch GADMA is given below.

#params_file :

# It is a parameters file for GADMA software.

Output directory:

/home2/datawork/[author]/INFERENCE/gadma/output_nonWF_5000_m005_L30000_styp

Input data:

/home2/datawork/[author]/INFERENCE/gadma/output_Cohort5000_m005.vcf,/home2/datawork/[author]/INFERENCE/gadma/pop_file_5000_m005_stypical.txt

Population labels: ['1', '2']

Projections: [50, 50]

Outgroup: False

Sequence length: Null

Linked SNP's: True

Directory with bootstrap: NULL

Engine: moments

Relative parameters: False

Theta0: 0.47

Mutation rate: Null

Time for generation: 6.98

Custom filename: Null

Lower bound: Null

Upper bound: Null

Parameter identifiers: Null

Initial structure: [1, 1]

Final structure: [1, 1]

Only sudden: False

No migrations: False

Symmetric migrations: True

Migration masks: Null

Split fractions: True

Inbreeding: False

Ancestral size as parameter: False

Upper bound of first split: Null

Upper bound of second split: Null

Local optimizer: optimize_powell

Print models' code every N iteration: 0

Model plot engine: moments

Draw models every N iteration: 0

Units of time in drawing: years

Vmin: 1

Silence: False

Verbose: 1

Number of repeats: 32

Number of processes: 16

Resume from: Null

Only models: False

**LITERATURE CITED IN THIS SUPPLEMENTARY FILE**

Adams AM, Hudson RR (2004). Maximum-likelihood estimation of demographic parameters using the frequency spectrum of unlinked single-nucleotide polymorphisms. *Genetics 168*: 1699–1712.

Akey JM, Zhang K, Xiong MM, Doris P & Jin L. (2001). The effect that genotyping errors have on the robustness of common linkage-disequilibrium measures. *American Journal of Human Genetics*, *68*(6), 1447–1456. <https://doi.org/10.1086/320607>

Anderson EC & Dunham KK. (2005). Spip 1.0: A Program for Simulating Pedigrees and Genetic Data in Age-Structured Populations. *Molecular Ecology Notes 5* (2): 459–61

Andreotti S, Rutzen M, van der Walt S, Von der Heyden S, Henriques R, Meÿer M, Oosthuizen H & Matthee C. (2016) An integrated mark-recapture and genetic approach to estimate the population size of white sharks in South Africa. *Marine Ecology Progress Series, 552*, 241–253.

Antao T, Pérez-Figueroa A, Luikart G. (2011). Early detection of population declines: high power of genetic monitoring using effective population size estimators. *Evol Appl. 4*(1):144-54.

doi: 10.1111/j.1752-4571.2010.00150.x

Barbato M, Orozco-terWengel P, Tapio M, Bruford MW. (2015). SNeP: a tool to estimate trends in recent effective population size trajectories using genome-wide SNP data. *Front Genet. 6*:109

Barría A, Christensen KA, Yoshida G, Jedlicki A, Leong JS, Rondeau EB, Lhorente JP, Koop BF, Davidson WS, Yáñez JM. (2019). Whole Genome Linkage Disequilibrium and Effective Population Size in a Coho Salmon (*Oncorhynchus kisutch*) Breeding Population Using a High-Density SNP Array. *Front Genet.10*:498. doi: 10.3389/fgene.2019.00498.

Beeravolu CR, Hickerson MJ, Frantz LAF & Lohse K. (2018). ABLE: Blockwise site frequency spectra for inferring complex population histories and recombination. *Genome Biology, 19*, 145.

<https://doi.org/10.1186/s13059-018-1517-y>

Blower DC, Pandolfi JM, Bruce BD, Gomez-Cabrera MD, Ovenden JR. (2012). Population genetics of Australian white sharks reveals fine-scale spatial structure, transoceanic dispersal events and low effective population sizes. *Mar Ecol Prog Ser 455*:229–244.

Blower DC, Riginos C & Ovenden JR. (2019) NeOGEN: A tool to predict genetic effective population size (Ne) for species with generational overlap and to assist empirical Ne study design. *Mol Ecol Resour*. 19: 260– 271. <https://doi.org/10.1111/1755-0998.12941>

Boitard S, Rodriguez W, Jay F, Mona S, Austerlitz F. (2016). Inferring population size history from large samples of genome-wide molecular data-an approximate Bayesian computation approach. *PLoS Genet. 12*:e1005877.

Bourgeois YXC & Warren BH. (2021). An overview of current population genomics methods for the analysis of whole-genome resequencing data in eukaryotes. *Molecular Ecology, 30*, 6036– 6071. <https://doi.org/10.1111/mec.15989>

Chabot C, Hawk HA, Allen LG. (2015). Low contemporary effective population size detected in the Critically Endangered giant sea bass, *Stereolepis gigas*, due to fisheries overexploitation. *Fisheries Research 172*,71-78. <https://doi.org/10.1016/j.fishres.2015.06.015>.

Corbin LJ, Liu AY, Bishop SC, Woolliams JA. (2012). Estimation of historical effective population size using linkage disequilibria with marker data. *J. Anim. Breed Genet.* 129, 257–270

Do C, Waples RS, Peel D, Macbeth G, Tillett BJ, Ovenden JR (2014). NeEstimator v2: re‐implementation of software for the estimation of contemporary effective population size (Ne) from genetic data. *Mol Ecol Resour 14*: 209–214.

Drummond AJ, Rambaut A, Shapiro B, Pybus OG. (2006) Bayesian coalescent inference of past population dynamics from molecular sequences. *Mol. Biol. Evol. 22*:1185–1192.

Dudgeon CL, Ovenden JR. (2015). The relationship between abundance and genetic effective population size in elasmobranchs: an example from the globally threatened zebra shark *Stegostoma fasciatum* within its protected range. *Conserv Genet 16,* 1443–1454.

<https://doi.org/10.1007/s10592-015-0752-y>

Durrett R. (2008). Probability Models for DNA Sequence Evolution (2nd ed.). Springer, New York.

Eldon B, Wakeley J. (2006). Coalescent processes when the distribution of offspring number among individuals is highly skewed. *Genetics 172*, 2621–2633.

England, P.R., Cornuet, J-M., Berthier, P., Tallmon, D.A., Luikart, G. (2006). Estimating effective population size from linkage disequilibrium: Severe bias using small samples. *Conservation Genetics*, 7(2), 303–308. https://doi.org/10.1007/s10592-005-9103-8

Ewing G, Hermisson J. (2010) MSMS: a coalescent simulation program including recombination, demographic structure and selection at a single locus. *Bioinformatics 26(16)*:2064-5. doi: 10.1093/bioinformatics/btq322.

Excoffier L and Foll M (2011). fastSimcoal: a continuous-time coalescent simulator of genomic diversity under arbitrarily complex evolutionary scenarios. *Bioinformatics, 27*(9):1332–1334.

doi: 10.1093/bioinformatics/btr124.

Excoffier L, Dupanloup I, Huerta-Sanchez E, Sousa VC, Foll M. (2013). Robust demographic inference from genomic and SNP data. *PloS Genet. 9*(10):e1003905.

Excoffier L, Marchi N, Marques DA, Matthey-Doret R, Gouy A, Sousa VC. (2021). fastsimcoal2: demographic inference under complex evolutionary scenarios. *Bioinformatics 37*(24):4882–5.

Fisher RA. (1930). The distribution of gene ratios for rare mutations. *Proc. R. Soc. Edinb*. 50: 205–220.

Franklin IR (1980). Evolutionary change in small populations. In M. Soulé & B. Wilcox (Eds.), Conservation biology: An evolutionary‐ecological perspective (pp. 135–149). Sunderland, MA: Sinauer Associates

Gargiulo R, Decroocq V, González-Martínez SC, Paz-Vinas I, Aury J-M, Lesur Kupin I, Plomion C, Schmitt S, Scotti I, and Heuertz M. (2024). Estimation of contemporary effective population size in plant populations: Limitations of genomic datasets. *Evolutionary Applications, 17*, e13691. <https://doi.org/10.1111/eva.13691>

Gilbert KJ, Whitlock MC. (2015). Evaluating methods for estimating local effective population size with and without migration: Estimating Ne in the presence of migration. *Evolution, 69*(8), 2154–2166. <https://doi.org/10.1111/evo.12713>.

Gutenkunst RN, Hernandez RD, Williamson SH, Bustamante CD. (2009). Inferring the joint demographic history of multiple populations from multidimensional SNP frequency data. Plos Genetics 5, e1000695.

Gutenkunst RN (2021) dadi.CUDA: Accelerating Population Genetics Inference with Graphics Processing Units, Molecular Biology and Evolution, 38(5), 2177–2178,

https://doi.org/10.1093/molbev/msaa305

Haller BC, Messer PW. (2018a). Evolutionary Modeling in SLiM 3 for Beginners. *Mol Biol Evol. 36*(5):1101-1109. doi: 10.1093/molbev/msy237.

Haller BC, Messer PW. (2018b). SLiM 3: forward genetic simulations beyond the Wright–Fisher model.
*Mol. Biol. Evol. 36*: 632–637. https://doi.org/10.1093/molbev/msy228.

Haller BC, Galloway J, Kelleher J, Messer PW, Ralph PL. (2018). Tree‐sequence recording in SliM opens new horizons for forward‐time simulation of whole genomes. *Mol Ecol Res. 19*:552–566.

<https://doi.org/10.1111/1755-0998.12968>

Hamilton MB, Tartakovsky M & Battocletti A. (2018) Speed-Ne: Software to simulate and estimate genetic effective population size (Ne) from linkage disequilibrium observed in single samples. *Mol Ecol Resour 18:*714–728. <https://doi.org/10.1111/1755-0998.12759>

Hayes BJ, Visscher PM, McPartlan HC, Goddard ME. (2003). Novel multilocus measure of linkage disequilibrium to estimate past effective population size. *Genome Res*. 13, 635–643

Hedrick P, Jain S, & Holden L (1978). Multilocus systems in evolution. *Evol Biol 11*:101–184.

Heller R, Chikhi L, & Siegismund HR. (2013). The confounding effect of population structure on Bayesian skyline plot inferences of demographic history. *PloS one, 8*(5), e62992.

Hill WG (1981). Estimation of effective population-size from data on linkage disequilibrium. *Genet Res 38*: 209–216.

Hollenbeck CM, Portnoy DS, Gold JR. (2016). A method for detecting recent changes in contemporary effective population size from linkage disequilibrium at linked and unlinked loci. *Heredity 117(4)*:207–216

Huang, X., Struck, T.J., Davey, S.W., Gutenkunst, R.N. (2023). dadi-cli: Automated and distributed population genetic model inference from allele frequency spectra. *bioRxiv*, 2023.06.15.545182. https://doi.org/10.1101/2023.06.15.545182

Hudson R.R. (1983). Properties of a neutral allele model with intragenic recombination. *Theor. Popul. Biol. 23*, 183–201.

Hudson RR, Kaplan NL. (1988). The coalescent process in models with selection and recombination. *Genetics 120*, 831–840.

Huang X, Struck TJ, Davey SW, Gutenkunst RN (2023). Dadi-cli: Automated and distributed population genetic model inference from allele frequency spectra. bioRxiv 2023.06.15.545182.

<https://doi.org/10.1101/2023.06.15.545182>

Jones AT, Ovenden JR & Wang Y-G. (2016). Improved confidence intervals for the linkage disequilibrium method for estimating effective population size. *Heredity, 117*, 217–223.

<https://doi.org/10.1038/hdy.2016.19>.

Jouganous J, Long W, Ragsdale AP, & Gravel S. (2017). Inferring the joint demographic history of multiple populations: beyond the diffusion approximation. *Genetics, 206*(3), 1549-1567

Jukes TH, Cantor CR. (1969) Evolution of Protein Molecules. In: Munro, H.N., Ed., Mammalian Protein Metabolism, Academic Press, New York, 21-132. <http://dx.doi.org/10.1016/B978-1-4832-3211-9.50009-7>.

Kamm J, Terhorst J, Song YS (2017). Efficient computation of the joint sample frequency spectra for multiple populations. *Journal of Computational and Graphical Statistics 26* (1), 182–194.

Kamm J, Terhorst J, Durbin R, Song YS (2020). Efficiently inferring the demographic history of many populations with allele count data. *Journal of the American Statistical Association, 115*(531), 1472–1487. <https://doi.org/10.1080/01621459.2019.1635482>.

Keinan A, Mullikin JC, Patterson N, Reich D (2007). Measurement of the human allele frequency spectrum demonstrates greater genetic drift in East Asians than in Europeans. *Nat Genet 39*: 1251–1255.

Kelleher J, Etheridge AM, McVean G. (2016). Efficient Coalescent Simulation and Genealogical Analysis for Large Sample Sizes. PLoS Comput Biol 12(5). <https://doi.org/10.1371/journal.pcbi.1004842>.

Kelleher J, Thornton KR, Ashander J, Ralph PL. (2018). Efficient pedigree recording for fast population genetics simulation. *PLoS Comput Biol 14*(11): e1006581.

<https://doi.org/10.1371/journal.pcbi.1006581>

Kern AD, Schrider DR. (2016). Discoal: flexible coalescent simulations with selection. *Bioinformatics 32*(24):3839-3841. doi: 10.1093/bioinformatics/btw556.

Kess T, Bentzen P, Lehnert SJ, Sylvester EVA, Lien S, Kent MP, Sinclair-Waters M, Morris CJ, Regular P, Fairweather R, Bradbury IR. (2019). A migration-associated supergene reveals loss of biocomplexity in Atlantic cod. *Science Advances 5*, eaav2461.

Kess T, Einfeldt AL, Wringe B, Lehnert SJ, Layton KKS, McBride MC, Robert D, Fisher J, Le Bris A, den Heyer C, Shackell N, Ruzzante DE, Bentzen P, Bradbury IR. (2021). A putative structural variant and environmental variation associated with genomic divergence across the Northwest Atlantic in Atlantic Halibut. *ICES Journal of Marine Science, 78*(7), 2371–2384.

<https://doi.org/10.1093/icesjms/fsab061>

King JR, Wetklo M, Supernaul J, Taguchi M, Yokawa K, Sosa-Nishizaki O, Withler RE (2015) Genetic analysis of stock structure of blue shark (*Prionace glauca*) in the north Pacific. *Fish Res 172*:181–189.

Kingman J.F.C. (1982). The coalescent. *Stochastic Process. Appl. 13*, 235–248.

Kopatz A, Eiken HG, Schregel J, Aspi J, Kojola I, Hagen SB. (2017). Genetic substructure and admixture as important factors in linkage disequilibrium-based estimation of effective number of breeders in recovering wildlife populations. *Ecol Evol.* 7:10721–10732.

<https://doi.org/10.1002/ece3.3577>

Kuguru G, Gennari E, Wintner S, Dicken ML, Klein JD, Rhode CR & Bester-van der Merwe AE. (2019) Spatio-temporal genetic variation of juvenile smooth hammerhead sharks in South Africa. *Marine Biology Research, 15*:10, 568-579, DOI: 10.1080/17451000.2019.1695058

Lauterbur ME. (2019) Coalescent models at small effective population sizes and population declines are positively misleading. bioRxiv, 705335.

Lehnert SJ, Kess T, Bentzen P. et al. (2019) Genomic signatures and correlates of widespread population declines in salmon. *Nat Commun 10*, 2996. <https://doi.org/10.1038/s41467-019-10972-w>

Leone A, Arnaud-Haond S, Babbucci M, Bargelloni L, Coscia I, Damalas D, Delord C, Franch R, Garibaldi F, Macias D, Mariani S, Martinsohn J, Megalofonou P, Micarelli P, Nikolic N, Prodöhl PA, Sperone E, Stagioni M, Zanzi A, Cariani A, Tinti F. (2024). Population genomics of the blue shark, *Prionace glauca*, reveals different populations in the Mediterranean Sea and the North East Atlantic. *Evolutionary Applications*, 17:e70005. <https://doi.org/10.1111/eva.70005>

Lewontin RC and Kojima K (1960). The evolutionary dynamics of complex polymorphisms. *Evolution 14,* 450–472.

Liu X and Fu Y-X. (2015). Exploring population size changes using SNP frequency spectra. *Nat Genet. 47*:555–9.

Luan P, Huo T, Ma B, Song D, Zhang X, Hu G. (2021). Genomic inbreeding and population structure of northern pike (*Esox lucius*) in Xinjiang, China. *Ecol Evol. 11*:5657– 5668.

<https://doi.org/10.1002/ece3.7469>

Lucena-Perez M, Marmesat E, Kleinman-Ruiz D, et al. (2020). Genomic patterns in the widespread Eurasian lynx shaped by Late Quaternary climatic fluctuations and anthropogenic impacts. *Mol Ecol. 29*: 812– 828. <https://doi.org/10.1111/mec.15366>.

Luikart G, Cornuet JM (1999) Estimating the effective number of breeders from heterozygote excess in progeny. *Genetics, 151*, 1211–1216.

Luikart G, Ryman N, Tallmon DA, Schwartz MK, Allendorf FW. (2010). Estimation of census and effective population sizes: the increasing usefulness of DNAbased approaches. *Conserv Genet. 11*:355–73

Macbeth GM, Broderick D, Buckworth RC, Ovenden JR. (2013). Linkage Disequilibrium Estimation of Effective Population Size with Immigrants from Divergent Populations: A Case Study on Spanish Mackerel (*Scomberomorus commerson*). *G3 (Bethesda) 3*(4), 709–717

Marandel, F, Lorance, P, Berthelé, O, Trenkel, VM, Waples, RS, Lamy, J-B. (2019) Estimating effective population size of large marine populations, is it feasible?. *Fish Fish*. 20: 189–198.

<https://doi.org/10.1111/faf.12338>.

Marandel, F, Charrier, G, Lamy, J-B, Le Cam, S, Lorance, P, Trenkel, VM. Estimating effective population size using RADseq: Effects of SNP selection and sample size. (2020) *Ecol Evol 10*: 1929–1937. <https://doi.org/10.1002/ece3.6016>

Martinez V, Dettleff PJ, Galarce N, Bravo C, Dorner J, Iwamoto RN, Naish K. (2022). Estimates of Effective Population Size in Commercial and Hatchery Strains of Coho Salmon (*Oncorhynchus kisutch* (Walbaum, 1792)). *Animals 12*(5):647. <https://doi.org/10.3390/ani12050647>

McVean GAT and Cardin NJ. (2005). Approximating the coalescent with recombination. *Philosophical Transactions of the Royal Society of London, Series B 360*, 1387–1393.

Montano V. (2016). Coalescent inferences in conservation genetics: should the exception become the rule? *Biol. Lett. 12*:20160211.

Nadachowska-Brzyska K, Dutoit L, Smeds L, Kardos M, Gustafsson L, Ellegren H. (2021). Genomic
inference of contemporary effective population size in a large island population of collared
flycatchers (*Ficedula albicollis*). *Molecular Ecology, 30*, 3965– 3973.

https://doi.org/10.1111/mec.16025

Neel M, McKelvey K, Ryman N, Lloyd MW, Short Bull R, Allendorf FW, Schwartz MK & Waples RS. (2013) Estimation of effective population size in continuously distributed populations: there goes the neighborhood. *Heredity 111*, 189–199 (2013). <https://doi.org/10.1038/hdy.2013.37>

Neuenschwander S, Michaud F, Goudet J. (2018). QuantiNemo 2: a Swiss knife to simulate complex demographic and genetic scenarios, forward and backward in time. *Bioinformatics 35(5):*886-888.

doi: 10.1093/bioinformatics/bty737.

Newby JN, Darden T, Shedlock AM (2014) Population genetic structure of spotted eagle rays, *Aetobatus narinari*, off Sarasota, Florida and the Southeastern United States. *Copeia 3*:503–512.

Nikolic N and Chevalet C. (2014). Detecting past changes of effective population size. *Evolutionary Applications, 7*(6), 663-681.

Nikolic N, Devloo-Delva F, Bailleul D, Noskova E, Rougeux C, Delord C, Borsa P, Liautard-Haag C, Hassan M, Marie AD, Feutry P, Grewe P, Davies C, Farley J, Fernando D, Biton-Porsmoguer S, Poisson F, Parker D, Leone A … Arnaud-Haond S. (2023). Stepping up to genome scan allows stock differentiation in the worldwide distributed blue shark *Prionace glauca*. *Molecular Ecology*, 32, 1000–1019. <https://doi.org/10.1111/mec.16822>

Noskova E, V. Ulyantsev, K. P. Koepfli, S. J. O'Brien, and P. Dobrynin. (2020.) GADMA: Genetic algorithm for inferring demographic history of multiple populations from allele frequency spectrum data. GigaScience, 9

Novo I, Santiago E, Caballero A. (2022) The estimates of effective population size based on linkage disequilibrium are virtually unaffected by natural selection. *PLoS Genet. 18*(1):e1009764. doi: 10.1371/journal.pgen.1009764

Nunziata SO and Weisrock DW. (2018) Estimation of contemporary effective population size and population declines using RAD sequence data. *Heredity 120*, 196–207.

<https://doi.org/10.1038/s41437-017-0037-y>

O’Leary SJ, Feldheim KA, Fields AT, Natanson LJ, Wintner S, Hussey N, Shivji MS, Chapman DD. (2015). Genetic diversity of white sharks, *Carcharodon carcharias*, in the Northwest Atlantic and Southern Africa. *J* *Hered 106*:1–8.

Pazmiño DA, Maes GE, Simpfendorfer CA, Salinas-de-León P, van Herwerden L. (2017) Genome-wide SNPs reveal low effective population size within confined management units of the highly vagile Galapagos shark (*Carcharhinus galapagensis*). *Conservation Genetics, 18*, 1151–1163

Peng B and Amos CI (2008) Forward-­time simulations of non-random mating populations using  
simuPOP. Bioinformatics 24(11), 1408-1409. <https://doi.org/10.1093/bioinformatics/btn179>

Perrier C, Rougemont Q, Charmantier A. (2020). Demographic history and genomics of local adaptation in blue tit populations. *Evol Appl. 13*: 1145– 1165. <https://doi.org/10.1111/eva.13035>

Portik DM, Leache AD, Rivera D, Barej MF, Burger M, Hirschfeld M, Rodel M-O, Blackburn DC, Fujita MK. (2017). Evaluating mechanisms of diversification in a Guineo-Congolian tropical forest frog using demographic model selection. *Mol Ecol. 26*(19):5245–5263

Pudovkin AI, Zaykin DV, Hedgecock D. (1996) On the potential for estimating the effective number of breeders from heterozygote-excess in progeny. *Genetics 144*:383–387.

Puncher GN, Cariani A, Maes GE, et al. (2018). Spatial dynamics and mixing of bluefin tuna in the Atlantic Ocean and Mediterranean Sea revealed using next-generation sequencing. *Mol Ecol Resour*. 18: 620– 638. <https://doi.org/10.1111/1755-0998.12764>.

Rangel-Morales, J.M., Rosales-López, L.P., Díaz-Jaimes, P. et al. (2022). Regional philopatry of scalloped hammerhead sharks (*Sphyrna lewini*) to nursery areas in the Mexican Pacific. *Hydrobiologia 849*, 3083–3099. <https://doi.org/10.1007/s10750-022-04880-2>.

Ragsdale AP, Gravel S. (2019). Models of archaic admixture and recent history from two-locus statistics.

*PLoS Genet. 15*(6):e1008204.

Ragsdale AP, Gravel S. (2020). Unbiased estimation of linkage disequilibrium from unphased data. *Mol Biol Evol. 37*(3):923–932.

Reid-Anderson S, Bilgmann K, Stow A. (2019). Effective population size of the critically endangered east Australian grey nurse shark *Carcharias taurus. Mar Ecol Prog Ser 610*:137-148.

<https://doi.org/10.3354/meps12850>

Robinson JD, Moyer GR. (2013). Linkage disequilibrium and effective population size when generations overlap. *Evol. Appl. 6*: 290–302.

Russell JC, and Fewster RM. (2009). Evaluation of the Linkage Disequilibrium method for estimating effective population size. In DL Thomson, EG Cooch & MJ. Conroy (Eds.), *Modeling Demographic Processes In Marked Populations* (pp. 291–320). Boston, MA: Springer,US

Ryman N, Laikre L & Hössjer O. (2019) Do estimates of contemporary effective population size tell us what we want to know? *Mol Ecol. 28*:1904–1918. <https://doi.org/10.1111/mec.15027>

Salmona J., Heller R., Lascoux M., Shafer A. (2017). Inferring Demographic History Using Genomic Data. In: Rajora, O. (eds) Population Genomics. Population Genomics. Springer, Cham.

https://doi.org/10.1007

Santiago E, Novo I, Pardiñas AF, Saura M, Wang J, Caballero A. (2020) Recent Demographic History Inferred by High-Resolution Analysis of Linkage Disequilibrium. *Molecular Biology and Evolution, (7)*:12, 3642-3653, <https://doi.org/10.1093/molbev/msaa169>

Saura M, Tenesa A, Woolliams JA, Fernandez A and Villanueva B. (2015). Evaluation of the linkage-disequilibrium method for the estimation of effective population size when generations overlap: an empirical case. *BMC Genomics 16*, 922. <https://doi.org/10.1186/s12864-015-2167-z>

Stanhope MJ, Ceres KM, Sun Q, Wang M, Zehr JD, Marra NJ, ... and Shivji MS. (2023). Genomes of endangered great hammerhead and shortfin mako sharks reveal historic population declines and high levels of inbreeding in great hammerhead. *Iscience, 26*(1), 105815.

Sved, J.A. (1971). Linkage disequilibrium and homozygosity of chromosome segments in finite populations. *Theoretical Population Biology*, 2(2), 125–141. https://doi.org/10.1016/0040-5809(71)90011-6

Sved, J.A., Cameron, E.C., & Gilchrist, A.S. (2013). Estimating effective population size from linkage disequilibrium between unlinked loci: Theory and application to fruit fly outbreak populations. *PLoS ONE*, 8(7), e69078. https://doi.org/10.1371/journal.pone.0069078

Tajima F. (1983). Evolutionary relationship of DNA sequences in finite populations. *Genetics 105*, 437–460.

Takahata N. (1988). The coalescent in two partially isolated diffusion populations. *Genet. Res., Camb. 53*, 213–222.

Tang Q, Shingate P, Wardiatno Y, John A, Tay BH, Tay YC, Yap L-M, Lim J, Tong HY, Tun K, Venkatesh B, Rheindt FE. (2021). The different fates of two Asian horseshoe crab species with different dispersal abilities. *Evolutionary Applications, 14*, 2124–2133. <https://doi.org/10.1111/eva.13271>.

Tellier A, Lemaire C. (2014) Coalescence 2.0: a multiple branching of recent theoretical developments and their applications. *Mol. Ecol. 23*, 2637–2652.

Uchino T, Nakamura Y, Sekino M, Kai W, Fujiwara A, Yasuike M, Sugaya T, Fukuda H, Sano M, Sakamoto T. (2016). Constructing Genetic Linkage Maps Using the Whole Genome Sequence of Pacific Bluefin Tuna (*Thunnus orientalis*) and a Comparison of Chromosome Structure among Teleost Species. *Advances in Bioscience and Biotechnology, 7*, 85-122. doi: 10.4236/abb.2016.72010.

Van de Putte AP, Van Houdt JKJ, Maes GE, Hellemans B, Collins MA, Volckaert FAM. (2012). High genetic diversity and connectivity in a common mesopelagic fish of the Southern Ocean: The myctophid *Electrona antarctica*. Deep Sea Research Part II: Topical Studies in Oceanography, Volumes 59–60, 199-207.

Wakeley J. (2020) Developments in coalescent theory from single loci to chromosomes. *Theor Popul Biol. 133*:56-64.

Wang J (2001). A pseudo-likelihood method for estimating effective population size from temporally spaced samples. *Genetical Research, 78*, 243–257.

Wang J (2009). A new method for estimating effective population size from a single sample of multilocus genotypes. *Molecular Ecology, 18*, 2148–2164.

Wang J (2016) A comparison of single-sample estimators of effective population sizes from genetic marker data. *Mol Ecol. 25*(19):4692-711. doi: 10.1111/mec.13725

Waples RS. (2006) A bias correction for estimates of effective population size based on linkage disequilibrium at unlinked gene loci. *Conserv Genet 7*, 167–184. <https://doi.org/10.1007/s10592-005-9100-y>.

Waples RS. (2024). Practical application of the linkage disequilibrium method for estimating contemporary effective population size: A review. *Molecular Ecology Resources, 24*, e13879

Waples RS and Do C (2008). LDNe: a program for estimating effective population size from data on linkage disequilibrium. *Mol Ecol Resour 8*: 753–756.

Waples RS and Do C (2010). Linkage disequilibrium estimates of contemporary *Ne* using highly variable genetic markers: a largely untapped resource for applied conservation and evolution. *Evol Appl 3:* 244–262.

Waples RS and England PR. (2011). Estimating contemporary effective population size on the basis of linkage disequilibrium in the face of migration. *Genetics, 189*, 633–644.

doi.org/10.1534/genetics.111.132233

Waples RS, Luikart G, Faulkner JR and Tallmon DA (2013). Simple life history traits explain key effective population size ratios across diverse taxa. *Proc. Biol. Sci. 280*: 20131339

Waples RS, Antao T & Luikart G (2014). Effects of overlapping generations on linkage disequilibrium estimates of effective population size. *Genetics, 197(2),* 769–780.

doi.org/10.1534/genetics.114.164822

Waples RK, Larson, WA & Waples RS. (2016). Estimating contemporary effective population size in non-model species using linkage disequilibrium across thousands of loci. *Heredity, 117*(4), 233–240. <https://doi.org/10.1038/hdy.2016.60>.

Waples RS, Grewe PM, Bravington MW, Hillary R, & Feutry P. (2018). Robust estimates of a high *Ne/N* ratio in a top marine predator, southern bluefin tuna. *Science Advances, 4*(7).

<https://doi.org/10.1126/sciadv.aar7759>

Waples RS, Waples RK & Ward EJ. (2022). Pseudoreplication in genomic-scale data sets. *Molecular Ecology Resources, 22*, 503– 518. <https://doi.org/10.1111/1755-0998.13482>

Wiuf C, and Hein J. (1999). Recombination as a point process along sequences. *Theoretical population biology, 55*(3), 248-259.

Wright S. (1931) Evolution in mendelian populations. *Genetics 16*, 97–15.
